# Bulkiness Makes the Difference: Modulating Solid-State Emission of Anticoronavirus Active Pt(tpy)C(O)NHCH_2_R Complexes Featuring Adamantyl vs Propargyl R Groups

**DOI:** 10.1021/acs.inorgchem.6c02335

**Published:** 2026-07-17

**Authors:** Mateusz Klarek, Daria Wysocka, Judith Füllborn-Ott, Damian Trzybiński, Krzysztof Woźniak, Paulina H. Marek-Urban, Piotr Pander, Ingo Ott, Rafał Nowak, Paweł Zmora, Konrad Kowalski

**Affiliations:** † 26527 University of Łódź, Faculty of Chemistry, Department of Organic Chemistry, Tamka 12, Łódź 91-403, Poland; ‡ Institute of Medicinal and Pharmaceutical Chemistry, Technische Universität Braunschweig, Beethovenstr. 55, Braunschweig 38-106, Germany; § Faculty of Chemistry, Biological and Chemical Research Centre, 49605 University of Warsaw, Żwirki i Wigury 101, Warsaw 02-089, Poland; ∥ Faculty of Chemistry, 49569 Silesian University of Technology, Strzody 9, Gliwice 44-100, Poland; ⊥ Faculty of Chemistry, Warsaw University of Technology, Noakowskiego 3, Warsaw 00-664, Poland; # Centre for Organic and Nanohybrid Electronics, Silesian University of Technology, Konarskiego 22B, Gliwice 44-100, Poland; ∇ Department of Physics, Durham University, South Road, Durham DH1 3LE, U.K.; ○ Department of Molecular Virology, Institute of Bioorganic Chemistry Polish Academy of Sciences, Noskowskiego 12/14, Poznan 61-704, Poland

## Abstract

Solid-state photoluminescence (PL) of Pt­(II) terpyridine-type (tpy) complexes relies on intermolecular Pt···Pt metallophilic interactions, and it can be tuned through bulky substituents disturbing them. We took advantage of this property by designing the Pt­(II) 2,2′:6′2”-tpy complexes **[L1PtCl]­PF**_**6**_ and **[L2PtCl]­PF**_**6**_ bearing either propargyl or 1-adamantyl connected to the tpy ligand. At 293 K, PL spectra of both complexes are characterized by broadband featureless orange-red emission (λ_PL_ ≈ 650 nm) characteristic of ^3^MMLCT excited states. This is an indication of short Pt···Pt contacts between individual molecules in powder. The PL spectra of **[L1PtCl]­PF**_**6**_ and **[L2PtCl]­PF**_**6**_ are significantly different from each other at 77 K. The former complex emits at 688 nm while the latter at 665 nm. This can be rationalized by the less sterically bulky ligand **L1** promoting the formation of larger aggregates, while the more bulky **L2** hinders this behavior. Unlike **[L2PtCl]­PF**_**6**_, the monohydrate **[L2PtCl]­PF**_**6**_·**H**_**2**_ **O** shows a vibronically resolved ^3^MMLCT+ ^3^LC emission in powder form at λ_PL_ = 567 nm at 293 K and 560 nm at 77 K. Such behavior indicates relaxation of the Pt···Pt contacts caused by trapped water molecules. **[L2PtCl]­PF**_**6**_ shows superior activity to **[L1PtCl]­PF**_**6**_ against the HCoV-OC43 and influenza A viruses.

## Graphical abstract

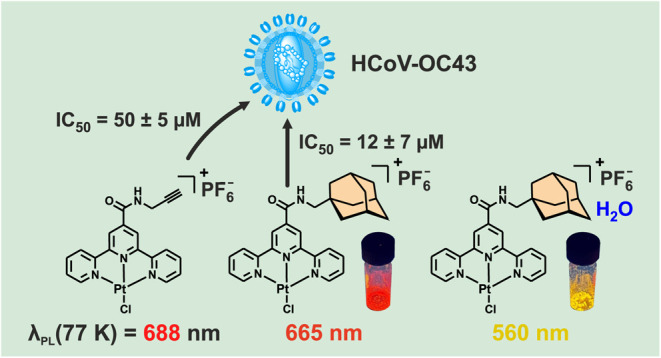

## Introduction

First reported nearly a hundred years ago, 2,2′:6′2″-terpyridine (tpy)^1^ is a cornerstone heterocyclic pincer ligand in coordination chemistry.^2–4^ Octahedral tpy complexes with transition metals have been studied for their electrochemical, magnetic, and other properties since the 1980s.^4,5^ Although the first square-planar complex [Pt­(tpy)­(Cl)]^+^Cl^–^-3H_2_O was isolated shortly after the tpy itself had been reported, it was not until the 1970s that the chemistry of square-planar tpy complexes with d^8^ late transition-metal ions, such as Pt­(II), Pd­(II), and Au­(III), raised popular interest.^6,4^ Among them, the square-planar Pt­(II) tpy complexes have received special attention due to interesting chemical, supramolecular, and photophysical properties, as well as their biological activity.^4,7–11^

Rapid deactivation *via* thermally accessible d-d states in the archetypical [Pt­(tpy)­(Cl)]^+^ complex itself and its simple congeners makes them nonemissive in solution.^12,13^ However, modification of the [Pt­(tpy)­(Cl)]^+^ core structure *via* substitution of the ancillary chloride with electron-rich ligands, introduction of π-electron-donating substituents in the 4′-tpy position, replacement of tpy with other suitably designed pincer cyclometalating ligands, or a combination thereof facilitates photoluminescence in solution.^11,12,14–19^ Unlike in solution, the [Pt­(tpy)­(Cl)]^+^-type complexes show photoluminescence in the solid state, in bulk/neat form, or sometimes in rigid matrices, making them attractive for applications in organic light-emitting diodes (OLEDs), for example.^11,12^ Solid-state emission of square-planar Pt-tpy complexes is often due to the favorable Pt···Pt metallophilic interactions between adjacent individual molecules within the network of agglomerates which they form, leading to the formation of metal-metal-to-ligand charge transfer (MMLCT) excited states. The lower-energy bi­(or poly)­molecular ^3^MMLCT excited states promote luminescence in aggregated Pt-tpy luminophores in respect to monomeric complexes due to suppression of otherwise vibrationally accessible d–d states.^20,21^ Likewise, tuning of that emission can be realized by structural modifications of the given complex or by the supramolecular alternations of the spatial structure of the agglomerates. The former approach requires ligand(s) modification,^22,23^ whereas the latter can be realized with anion exchange,^20^ solvation,^24–26^ or grinding.^27^ Hence, in this work, we present a study which sheds new light on the aggregation of [Pt­(tpy)­(Cl)]^+^-type complexes and the steric effects affecting Pt···Pt interactions in the solid state.

On the other hand, the significance of cisplatin anticancer drugs^28^ motivated continued interest across biology and medicine for the development of platinum­(II) terpyridine-type complexes.^4,7,10,29–35^ In this respect, they have shown an array of interesting biological activities, including antitrypanosomal^29,30^ and anticancer^31–33^ activities, cysteine protease inhibition,^34^ and have been utilized as luminescent bioimaging and biosensing tools.^10,35^ In comparison to that, the antiviral activity of Pt complexes is at its infancy.^36,37^ Concerning Pt­(II)­(tpy) complexes, to the best of our knowledge, there exists only one publication reporting on antiviral-related activity of the archetypal [Pt­(tpy)­(Cl)]^+^Cl^–^ complex as well as its two cyclometallated congeners against severe acute respiratory syndrome coronavirus 2 (SARS-CoV-2) protein targets.^38^ The [Pt­(tpy)­(Cl)]^+^Cl^–^ complex shows moderate (∼25%) ability for inhibition of the spike protein interaction with the ACE2 receptor, which is of relevance when the virus enters the host cell.^38^ Furthermore, it also shows inhibitory activity of SARS-CoV-2 protease PL^pro^, which is essential for replication of the virus in infected cells, with 75% inhibition at 10 μM concentration.^38^

Owing to the increasing importance of Pt­(II)-terpyridine-type complexes as solid-state photoluminescence emitters, we herein report on the synthesis, in-depth photophysical characterization, and DFT calculations of the two novel Pt­(II) tpy complexes **[L1PtCl]­PF**_**6**_ and **[L2PtCl]­PF**_**6**_ (Scheme 2). These compounds were rationally designed to have “L” ligands characterized by different bulkiness. To accomplish this task, we utilized linear propargylamide and the 3D sterically demanding adamantylamide group as structural components of **L1** and **L2** ligands, respectively. We hypothesize that alternations of ligand bulkiness will modulate the extent of Pt···Pt interactions in aggregates of **[L1PtCl]­PF**_**6**_ and **[L2PtCl]­PF**_**6**_ and therefore tune their photophysical properties like photoluminescence. It is important to mention that propargyl complex **[L1PtCl]­PF**_**6**_ represents potentially valuable material for further derivatization via cycloaddition and Pd-catalyzed cross-coupling reactions. Further, we report on a solvate **[L2PtCl]­PF**_**6**_·**H**_**2**_ **O** whose photophysical characteristics in the solid state contrast those of the anhydrous **[L2PtCl]­PF**_**6**_ complex as a result of a water-induced relaxation of aggregates. In order to acquire a more comprehensive knowledge of various kinds of activities of the **[L1PtCl]­PF**_**6**_ and **[L2PtCl]­PF**_**6**_ complexes, we have also studied them as antiviral agents against the HCoV-OC43 (a surrogate of SARS-CoV-2) and influenza virus A/Puerto Rico/8/34 (IAV). The antiviral activity examinations were further justified by scarce reports on anti-SARS-CoV-2 activity studies of platinum complexes and the fact that adamantanes such as amantadine and rimantadine are well-known antiviral drugs.^39^ It was then reasoned to verify how the combination of Pt-tpy and adamantyl subunits’ properties influences antiviral activity.

## Results and Discussion

### Synthesis

The propargylamide tpy (**L1**) and adamantylamide tpy (**L2**) ligands were synthesized by the *N,N*′-diisopropylcarbodiimide- and *N*-hydroxysuccinimide-mediated condensation of the 2,2′:6′,2″-terpyridine-4′-carboxylic acid^40^ (**tpyCOOH**) with propargylamine and 1-adamantanemethylamine according to Scheme 1. Compounds **L1** and **L2** were obtained as crystalline white solids in 91 and 83% yields, respectively.

**Scheme 1:**
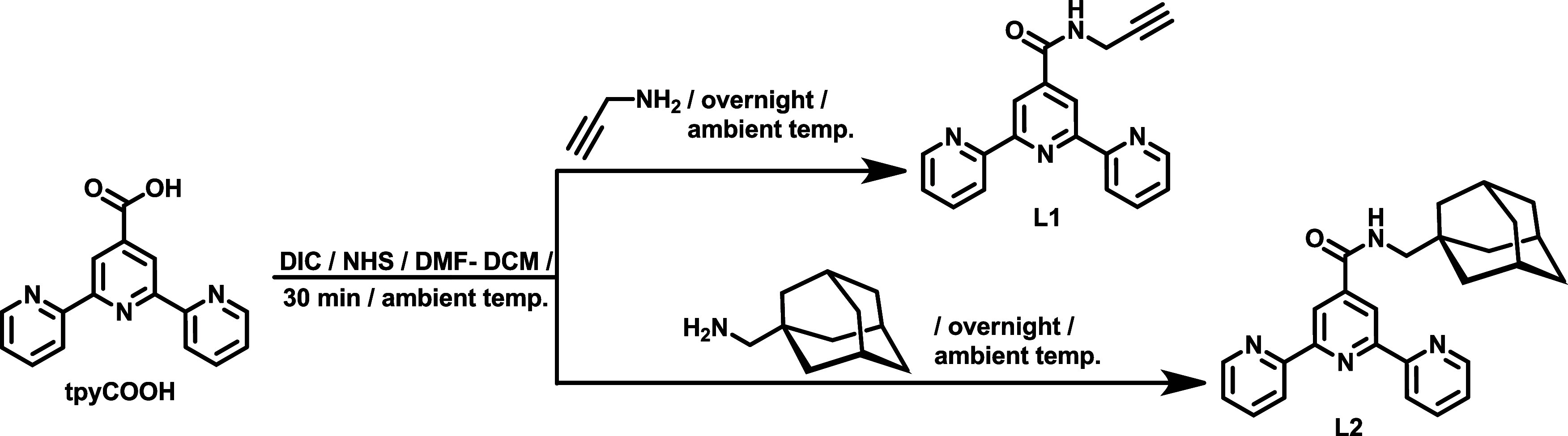
Synthesis of Ligands **L1** and **L2**

In the next step, the synthesis of complexes **[L1PtCl]­PF**_**6**_, **[L2PtCl]­PF**_**6**_ **·H**_**2**_ **O**, and **[L2PtCl]­PF**_**6**_ was carried out (Scheme 2). It utilized a thermally driven reaction of the platinum precursor complex *cis*-[PtCl_2_(COD)] with **L1** or **L2** in a H_2_O/DMF mixture. After the Pt-coordination step, the reaction mixture was cooled to ambient temperature and treated with NH_4_PF_6_. Thus formed salt suspension was left at 4 °C overnight. Then, complexes **[L1PtCl]­PF**_**6**_ and **[L2PtCl]­PF**_**6**_ **·H**_**2**_ **O** were isolated in analytically pure forms after workup comprising centrifugation, filtration, washing, and drying. The water-free compound **[L2PtCl]­PF**_**6**_ was obtained directly after precipitation with NH_4_PF_6_ (Scheme 2). Complexes **[L1PtCl]­PF**_**6**_, **[L2PtCl]­PF**_**6**_, and **[L2PtCl]­PF**_**6**_ **·H**_**2**_ **O** are red, orange, and yellow solids, respectively. They were obtained in 86, 65, and 51% yields, respectively.

**Scheme 2:**
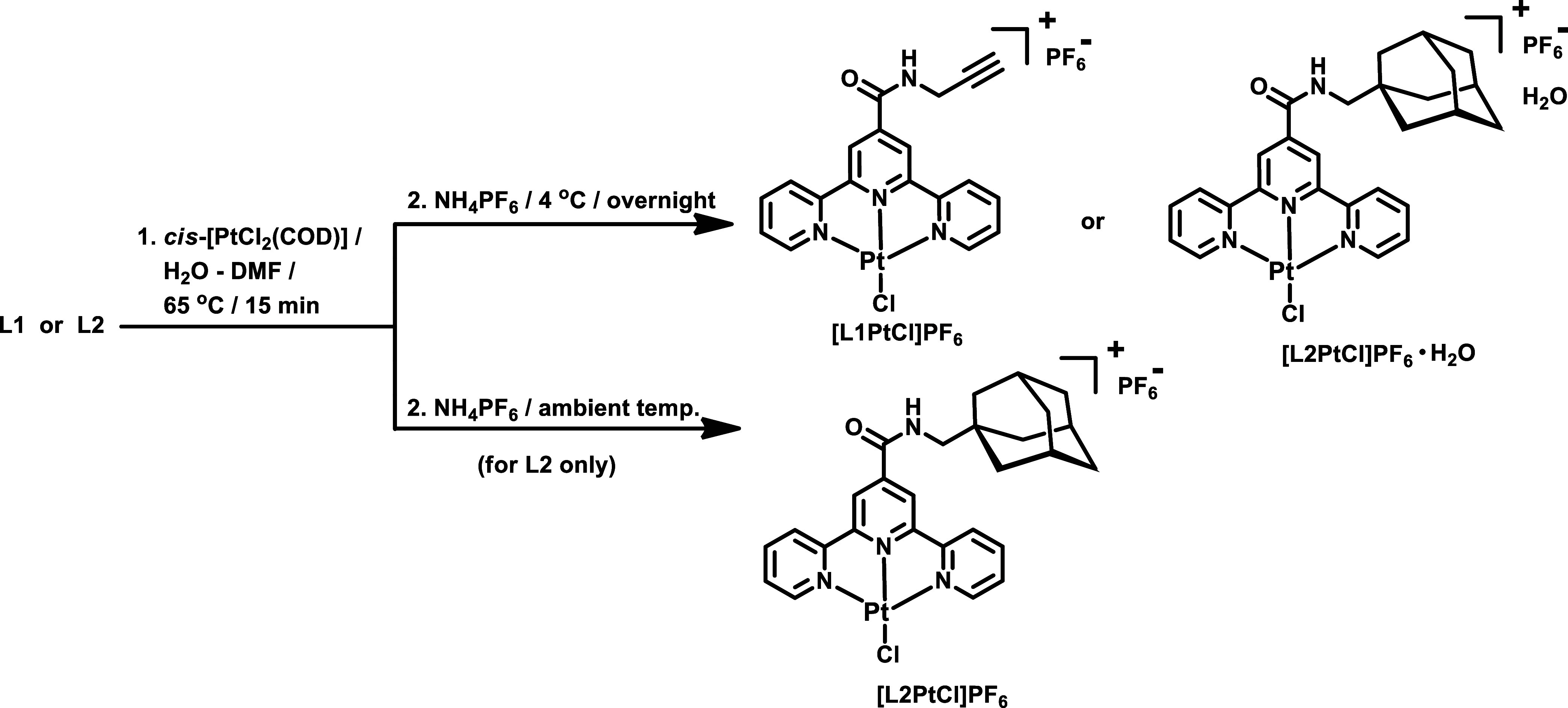
Synthesis of Complexes **[L1PtCl]­PF**_**6**_, **[L2PtCl]­PF**_**6**_ **·H**_**2**_ **O**, and **[L2PtCl]­PF**_**6**_

The structures and purity of ligands **L1** and **L2**, as well as their corresponding Pt­(II) complexes, were confirmed by ^1^H, ^13^C NMR and IR spectroscopy, high-resolution mass spectrometry (HRMS), and elemental analysis. Analytical data confirmed the postulated structures of the products. The ^1^H, ^13^C NMR, and HRMS spectra are shown in Figures S1–S30 (see the Supporting Information). Furthermore, the structures of **L1** and **L2** in the solid state were determined by single-crystal X-ray structural analysis. Water content in solvate **[L2PtCl]­PF**_**6**_ **·H**_**2**_ **O** was assessed using thermogravimetric and elemental analysis (Figures S31–S33). We note that the formation of solvates has also been observed for other square-planar Pt­(II) pincer ligand complexes.^41^ The ^1^H NMR spectra of **[L1PtCl]­PF**_**6**_ and **[L2PtCl]­PF**_**6**_ show typical signals assigned to tpy protons and the signals of protons characteristic of C­(O)­NH-CH_2_-R substituents. For example, protons of the amide NH groups in **[L1PtCl]­PF**_**6**_ and **[L2PtCl]­PF**_**6**_ resonate as triplets at 9.45 and 8.87 ppm, respectively. Protons of the methylene groups resonate at 4.24 (**[L1PtCl]­PF**_**6**_) and 3.13 (**[L2PtCl]­PF**_**6**_) ppm. The ^1^H NMR spectrum of **[L1PtCl]­PF**_**6**_ shows a characteristic one-proton resonance signal for the ≡CH propargyl proton at 3.35 ppm. The presence of the 1-adamantyl group is unambiguously manifested in the ^1^H NMR spectrum of **[L2PtCl]­PF**_**6**_ by the set of four signals at 1.98, 1.69, 1.64, and 1.58 ppm with a total proton count of fifteen. Presence of the propargyl moiety in **L1** and **[L1PtCl]­PF**_**6**_ was also confirmed through the characteristic ν_cc_ vibration bands at 2125 and 2121 cm^–1^ in the FTIR spectra.

### X-ray Structure Determination

The molecular structure of the ligands **L1** and **L2** in the solid state was determined by single-crystal X-ray diffraction analysis. Crystals suitable for this purpose were obtained by slow diffusion of *n*-pentane into saturated ethyl acetate solutions of the respective compounds at ambient temperature. Crystallographic and structure refinement details are summarized in Table S1, while comprehensive lists of bond lengths (Å) and valence and torsion angles (°) are provided in Tables S2–S7. Molecular structures of **L1** and **L2** are shown in Figures 1 and 2 together with selected bond lengths and valence and torsion angles.

**Figure 1:**
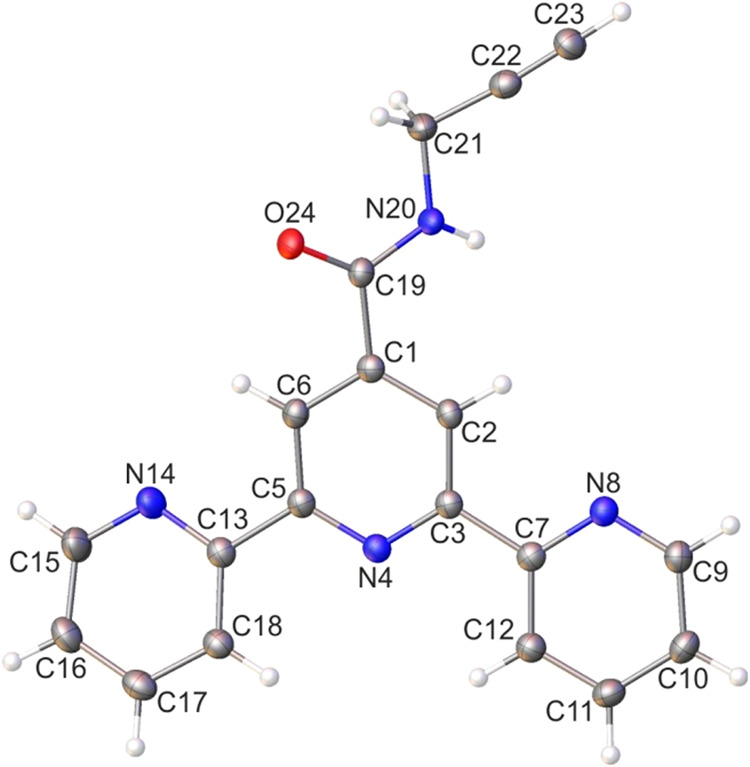
Molecular diagram of **L1** with atomic displacement ellipsoids at the 50% probability level. The H atoms are shown as small spheres of arbitrary radius. Selected bond lengths (Å) and angles (^o^): C19–024, 1.2381(14); C19–N20, 1.3343(15); C5–N4, 1.3469(16); C13–N14, 1.3468(16); C7–N8, 1.3445(15); C3–C7, 1.4944(16); C5–C13, 1.4882(16); C22–C23, 1.1865(19); C6–C1–C19, 117.45(10); C1–C19–O24, 119.96(10); C1–C19–N20, 117.52(10); C21–C22–C23, 175.43(13); C1–C19–N20–C21, 175.85(10).

**Figure 2:**
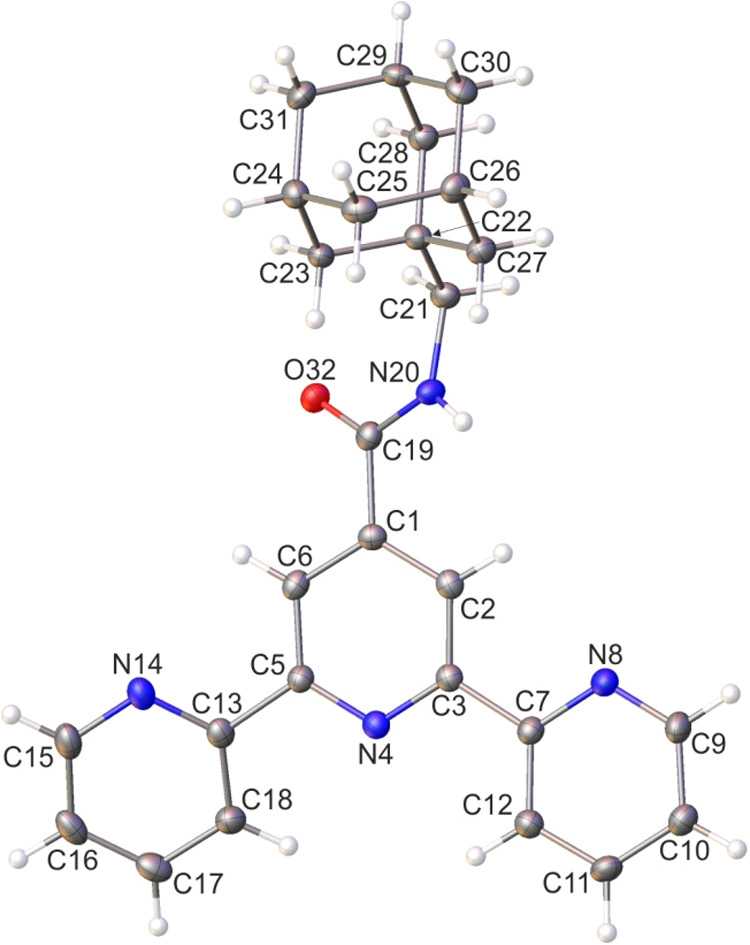
Molecular diagram of **L2** with atomic displacement ellipsoids drawn at the 50% probability level. The H atoms are shown as small spheres of arbitrary radius. Selected bond lengths (Å) and angles (^o^): C19–O23, 1.240(3); C19–N20, 1.346(3); C5–N4, 1.344(3); C21–N20, 1.461(3); C13–N14, 1.337(3); C7–N8, 1.346(3); C21–C22, 1.525(3); C3–C7, 1.488(3); C5–C13, 1.491(3); C6–C1–C19 118.3(2); C1–C19–O32, 120.1(2); C1–C19–N20, 116.3(2); C1–C19–N20–C21, 168.3(2).

Compounds **L1** and **L2** crystallize in the monoclinic space group *P*2_1_/*n* and the orthorhombic space group *Pbca*, respectively, each with one independent molecule in the asymmetric unit. The crystallographic analysis confirmed the proposed molecular structures of **L1** and **L2**, in which the respective amide–R substituents are attached to the 4′-position of the tpy scaffold. In both compounds, the tpy adopts the most energetically favored *trans,trans*-conformation. For **L1**, the dihedral angles between the central and terminal pyridine rings are 4.28(4)° and 13.07(4)°, respectively. In the molecule of **L2**, these angles are slightly larger (5.78(8)° and 22.74(8)°). Furthermore, the carbonyl groups of the amide moieties are noticeably tilted out of the central pyridine ring planes in both molecules. The angles between the mean planes defined by C1/C19–N20/O24 and C1–C6 atoms are 29.79(4)° for **L1** and 25.55(8)° for **L2**, respectively. Both compounds exhibit a similar degree of space filling in the unit cells (approximately 67%). However, due to the substitution of tpy in the 4′ position with differently sterically hindered groups (ethynyl vs 1-adamantyl), the crystal-network architecture for **L1** and **L2** varies.

In the crystal lattice of **L1**, the adjacent molecules assemble in stacks, which are stabilized by the chains of N–H···O hydrogen bonds (Figure S34 and Table S8). These chains extend along the crystallographic *a*-direction and are further stabilized by π–π stacking interactions (Table S9) involving the central pyridine and one of the peripheral pyridine rings of the tpy scaffold. Individual molecules in neighboring chains interact with one another through weak C–H···O and C–H···N contacts (Table S8), eventually giving rise to the three-dimensional supramolecular architecture shown in Figure S34. The hydrogen-bonded chains of molecules are also present in the crystal lattice of **L2** (Figure S35). However, in this case, chains propagate along the *b*-direction and adopt a distinct cross-like spatial arrangement (Figure S35, top view). This is because they lie on a glide plane (whereas in **L1**, the molecules within the chain are related only through translation). The above supramolecular framework is stabilized by bifurcated N–H···O and C–H···O interactions converging at a common acceptor (Table S10). Adjacent molecules in neighboring chains further interact via a network of weak C–H···π contacts between them (Table S11), involving a hydrogen atom of the adamantyl moiety and the central ring of the terpyridine unit, extending along the *a*-direction (Figure S35).

### Optical Spectroscopy

**[L1PtCl]­PF**_**6**_, **[L2PtCl]­PF**_**6**_, and **[L2PtCl]­PF**_**6**_ **·H**_**2**_ **O** display distinct color and photoluminescence in powder, and we decided to investigate this phenomenon further. We note that these molecules are virtually nonemissive in aerated or degassed DMSO, which is generally expected for Pt­(II) complexes of *NNN*-coordinating ligands.^42^ This is in stark contrast with their structurally similar *NCN* analogues.^43^ Absorption spectra recorded in DMSO are presented in Figure 3a, while their excitation and photoluminescence spectra in powder are shown in Figures 3b,c and S36, respectively. Although solution is likely the most popular environment for luminescence studies, solid-state photoluminescence is often the target for several applications, like OLEDs^44^ or solid-state optical sensor materials.^45^ For this reason, we decided to study the photoluminescence of powders more in-depth.

**Figure 3:**
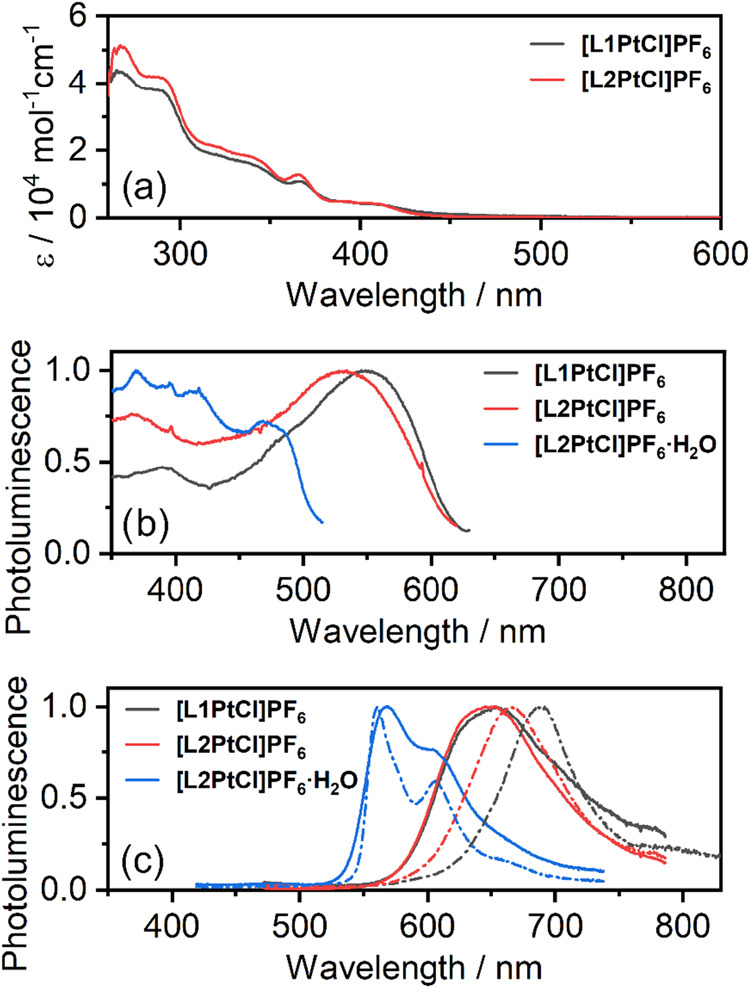
(a) Absorption spectra in DMSO. (b) Excitation spectra of **[L1PtCl]­PF**_**6**_ (λ_col_ = 650 nm), **[L2PtCl]­PF**_**6**_ (λ_col_ = 650 nm), and **[L2PtCl]­PF**_**6**_ **·H**_**2**_ **O** (λ_col_ = 570 nm) in powder form, recorded using collection wavelengths displayed in parentheses. (c) Steady-state photoluminescence spectra of **[L1PtCl]­PF**_**6**_, **[L2PtCl]­PF**_**6**_, and **[L2PtCl]­PF**_**6**_ **·H**_**2**_ **O** in powder form, recorded at 293 K (solid lines) or at 77 K (dotted and dashed lines).

The photoluminescence spectra of **[L1PtCl]­PF**_**6**_ and **[L2PtCl]­PF**_**6**_ powders recorded at 293 K are virtually the same and resemble the photoluminescence of aggregates of other Pt­(II) complexes emitting from their ^3^MMLCT states.^46,47^ The PL spectra alone are a strong indication of the existence of short (*i.e*., ∼3 Å) Pt···Pt contacts in the solid state of these complexes. This is contrasted by the photoluminescence spectrum of **[L2PtCl]­PF**_**6**_ **·H**_**2**_ **O** powder, which resembles that of an ^3^MLCT+^3^LC state, *i.e*., with a vibronically resolved structure associated with the ligand-centered character admixture. Given the formally identical nature of the luminophore in both **[L2PtCl]­PF**_**6**_ and **[L2PtCl]­PF**_**6**_ **·H**_**2**_ **O**, the difference in the PL spectrum between them must be related to the immediate surroundings of the **[L2PtCl]** ^**+**^ cation. We believe that the presence of the pendant adamantyl group in ligand **L2** relaxes aggregation between the two Pt­(*NNN*)-Cl units, however, without breaking the short Pt···Pt contacts. An additional H_2_O molecule in **[L2PtCl]­PF**_**6**_ **·H**_**2**_ **O** is likely to form hydrogen bonding interactions between the ions, acting as an intercalator and breaking the Pt···Pt interactions already weakened by the bulky adamantyl.

Excitation spectra in powder, shown in Figure 3b, are fully in line with the above interpretation. The excitation spectrum of **[L2PtCl]­PF**_**6**_ **·H**_**2**_ **O** powder is somewhat similar to the absorption spectrum in solution shown in Figure 3a, with an onset at ∼510 nm and in agreement with the ^3^MLCT photoluminescence. We observe a clear presence of MMLCT absorption bands in **[L1PtCl]­PF**_**6**_ and **[L2PtCl]­PF**_**6**_ powders, with onsets at ∼620 nm, clearly red-shifted with respect to the excitation spectrum of **[L2PtCl]­PF**_**6**_ **·H**_**2**_ **O**, and in agreement with the ^3^MMLCT photoluminescence shown in Figure 3c. Furthermore, we observe a subtle difference between the maxima of the two MMLCT absorption bands in question: λ_abs_ = 550 nm in **[L1PtCl]­PF**_**6**_ and λ_abs_ = 533 nm in **[L2PtCl]­PF**_**6**_, highlighting the electronic differences in aggregates formed by the two complexes. We have not been able to alter the photoluminescence and color of the hydrated **[L2PtCl]­PF**_**6**_ **·H**_**2**_ **O** by using a heat gun, as it retains its original optical properties after heating (Figure S36). We believe it may suggest that dehydration of the sample does not alter the original molecular arrangement of the complex in the solid and that the red-emissive **[L2PtCl]­PF**_**6**_ anhydrous form cannot be produced simply by dehydrating the yellow-emitting **[L2PtCl]­PF**_**6**_ **·H**_**2**_ **O** form.

We have performed pXRD experiments for all three samples (details are shown in the Experimental section) with the diffractograms shown in Figure 4. All of the samples exhibit moderate crystallinity with a significant amorphous component. Comparison of powder patterns of **[L2PtCl]­PF**_**6**_ and **[L2PtCl]­PF**_**6**_ **·H**_**2**_ **O** shows significant differences, indicating different phases, which aligns with the TGA-derived data. Significant peaks broadening in all samples translate to a small crystallite size. Crystal sizes estimated from the Sherrer equation for all samples are as follows: 45.3, 25.1, and 30.0 nm for **[L1PtCl]­PF**_**6**_, **[L2PtCl]­PF**_**6**_, and **[L2PtCl]­PF**_**6**_ **·H**_**2**_ **O**, respectively. Small differences in the crystallite size and partial sample amorphization might suggest that emission properties are derived from intermolecular interactions rather than macroscopic effects such as reabsorption.

**Figure 4:**
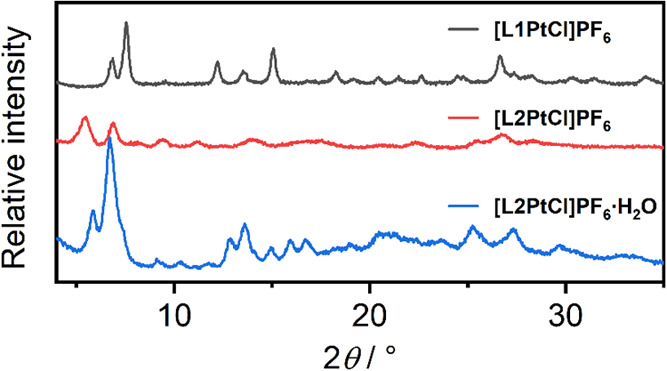
Powder XRD (pXRD) diffractograms obtained for **[L1PtCl]­PF**_**6**_, **[L2PtCl]­PF**_**6**_, and **[L2PtCl]­PF**_**6**_ **·H**_**2**_ **O**.

A further investigation into the PL of **[L1PtCl]­PF**_**6**_, **[L2PtCl]­PF**_**6**_, and **[L2PtCl]­PF**_**6**_ **·H**_**2**_ **O** in powders reveals additional details about their luminescent behavior. When investigated at 77 K, the PL of **[L2PtCl]­PF**_**6**_ **·H**_**2**_ **O** is expectedly similar to that at 293 K, but with a more resolved vibronic structure. This is in agreement with the typical behavior of the emission originating from the ^3^MLCT states. Unlike **[L2PtCl]­PF**_**6**_ **·H**_**2**_ **O**, **[L1PtCl]­PF**_**6**_ and **[L2PtCl]­PF**_**6**_ behave somewhat unexpectedly, with their PL spectra at 77 K being significantly red-shifted in relation to the spectra at ambient temperature. More so, the PL of **[L1PtCl]­PF**_**6**_ at 77 K, λ_PL_ = 688 nm, is different from that of **[L2PtCl]­PF**_**6**_, λ_PL_ = 665 nm, despite their respective PL spectra being identical at 293 K. We find that this behavior is similar to one previously observed for analogous Pt­(*NCN*)-X complexes.^22,41^ Following this earlier explanation, we believe that in the case of **[L1PtCl]­PF**_**6**_ and **[L2PtCl]­PF**_**6**_, dimers emitting at λ_PL_ = 650 nm are the dominating species, but larger structures, such as trimers or tetramers, may also be present. Larger aggregates form analogous but lower-energy ^3^MMLCT excited states, appropriately to their size, which form shallow traps in the solid state. At ambient temperature, there is sufficient thermal energy for the higher-energy and usually more luminescent dimer ^3^MMLCT states to emit, but at lower temperatures, this is no longer true, and lower-energy states dominate the emission. The distinct PL spectra of **[L1PtCl]­PF**_**6**_ and **[L2PtCl]­PF**_**6**_ originate from different sizes of aggregates existing in the solid state. It is highly likely that the bulky adamantyl group effectively suppresses aggregation, limiting the size of oligomers.

Photoluminescence decay traces of **[L1PtCl]­PF**_**6**_, **[L2PtCl]­PF**_**6**_, and **[L2PtCl]­PF**_**6**_ **·H**_**2**_ **O** powders are presented in Figure 5. PL decay on its own cannot be directly translated into radiative decay rates; however, we make several conclusions due to the similarity between the three samples. All decay traces present biexponential luminescent decay originating from the existence of more than one emitting center in the solid state. We discuss that in detail in the next paragraph. First, **[L2PtCl]­PF**_**6**_ **·H**_**2**_ **O** displays the longest decay lifetime at ambient temperature, τ_av_ = 3.8 μs, at least an order of magnitude longer than that of **[L1PtCl]­PF**_**6**_ and **[L2PtCl]­PF**_**6**_, with *k*_r_ = 4.5 × 10^4^ s^–1^, smaller than that in the anhydrous complexes by a factor of ∼20. This is in line with the usual behavior of ^3^MLCT+^3^LC vs ^3^MMLCT excited states, as the former generally displays slower luminescent decay.

**Figure 5:**
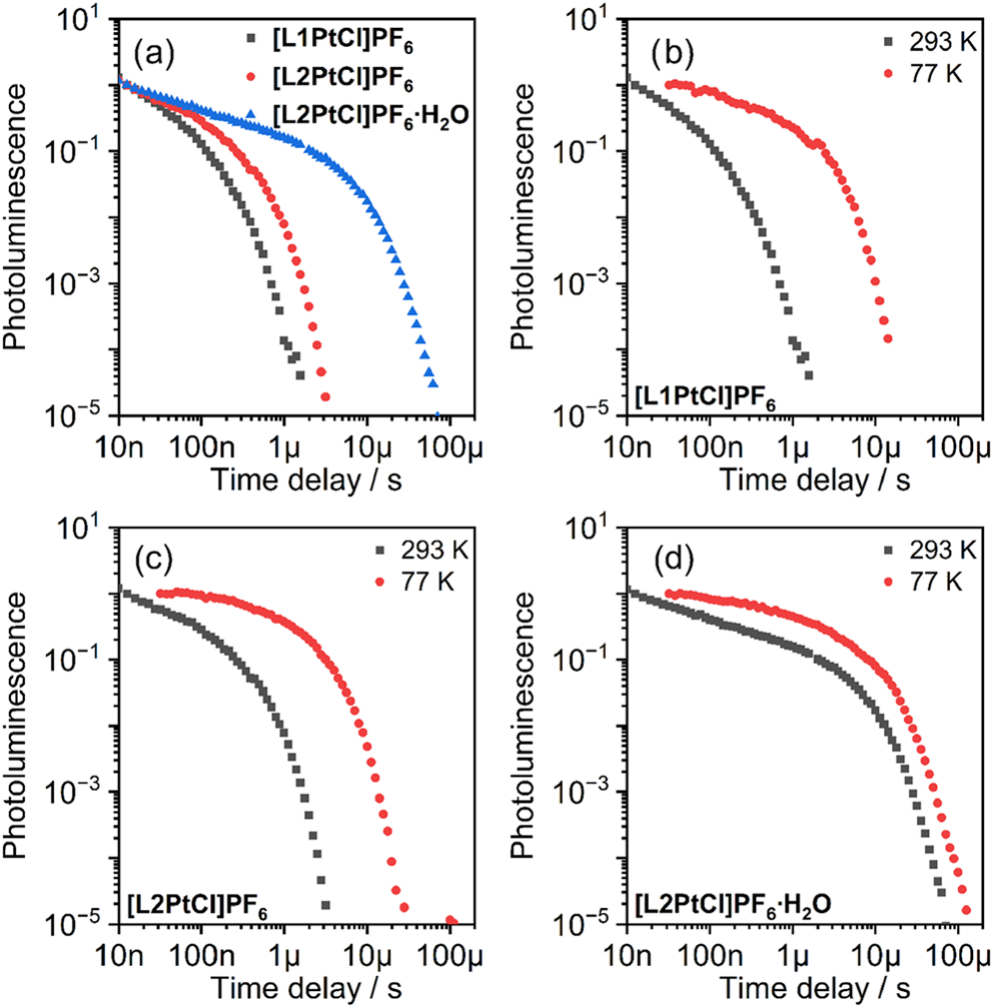
Photoluminescence decay traces of complexes **[L1PtCl]­PF**_**6**_, **[L2PtCl]­PF**_**6**_, and **[L2PtCl]­PF**_**6**_ **·H**_**2**_ **O** in powders: (a) comparison of decay traces at 293 K; (b–d) decay traces recorded at 293 and 77 K.

Anhydrous complexes **[L1PtCl]­PF**_**6**_ and **[L2PtCl]­PF**_**6**_ feature smaller τ_av_ than the hydrate **[L2PtCl]­PF**_**6**_ **·H**_**2**_ **O**, mainly due to more pronounced nonradiative decay rates of the more red-shifted ^3^MMLCT emissions, in line with the energy gap law. **[L1PtCl]­PF**_**6**_ displays a shorter τ_av_ = 0.06 μs than its counterpart, at τ_av_ = 0.20 μs, due to the presence of larger and lower-emissive aggregated species in the former.^22,41^ The photoluminescence quantum yields (Φ_PL_) are 0.09 and 0.27, respectively for **[L1PtCl]­PF**_**6**_ and **[L2PtCl]­PF**_**6**_, which aligns with the conclusions made above. These translate to *k*_r_ = 1.5 × 10^6^ s^–1^ and *k*_nr_ = 1.5 × 10^7^ s^–1^ in **[L1PtCl]­PF**_**6**_ and *k*_r_ = 1.4 × 10^6^ s^–1^ and *k*_nr_ = 3.7 × 10^6^ s^–1^ in **[L2PtCl]­PF**_**6**_. We note that while the *k*_r_ is virtually identical in both of these complexes, the *k*_nr_ is smaller in **[L2PtCl]­PF**_**6**_ by a factor of 4, thanks to the fine steric effect induced by the adamantyl group. While we have no doubt that quenching of excited states by molecular oxygen is negligible for these two samples due to the short PL lifetime, the same cannot be said about **[L2PtCl]­PF**_**6**_ **·H**_**2**_ **O**. We have recorded Φ_PL_ = 0.17 in air for the longer-lived PL in **[L2PtCl]­PF**_**6**_ **·H**_**2**_ **O**, which is likely an underestimate due to the luminescence quenching by molecular oxygen in this case. Table 1 summarizes photophysical properties of complexes **[L1PtCl]­PF**_**6**_, **[L2PtCl]­PF**_**6**_, and **[L2PtCl]­PF**_**6**_ **·H**_**2**_ **O** at 293 and 77 K, respectively.

**Table 1:** Summary of Photophysical Properties of Complexes **[L1PtCl]­PF**_**6**_, **[L2PtCl]­PF**_**6**_, and **[L2PtCl]­PF**_**6**_ **·H**_**2**_ **O**

	**293 K**							**77 K**		
**sample**	λ_abs_, nm (ε, 10^3^ M^–1^cm^–1^)^a^	λ_PL_, nm^b^	Φ_PL_ ^c^	τ, μs^d^	τ_av_, μs^e^	*k*_r_, s^–1^ ^f^	*k*_nr_, s^–1^ ^g^	λ_PL_, nm^h^	τ, μs^i^	τ_av_, μs^j^
**[L1PtCl]PF**_**6**_	410sh (4100), 366 (10700), 340sh (15500), 290sh (37500), 266 (43323)	653	0.09	0.08 (64%), 0.02 (36%)	0.06	1.5 × 10^6^	1.5 × 10^7^	688	1.9 (77%), 0.43 (23%)	1.6
**[L2PtCl]PF**_**6**_	410sh (4100), 365 (12700), 340 (17600), 290 (41500), 266 (50800)	650	0.27	0.30 (59%), 0.07 (41%)	0.20	1.4 × 10^6^	3.7 × 10^6^	665	1.8 (89%), 0.27 (11%)	1.6
**[L2PtCl]PF**_**6**_ **·H**_**2**_ **O**		567, 606sh	0.17	4.5 (85%), 0.65 (15%)	3.8	4.5 × 10^4^	2.2 × 10^5^	560, 606, 661sh	6.8 (85%), 1.0 (15%)	5.9

a Absorption maxima in DMSO.

b Photoluminescence maxima in powder at 293 K.

c Photoluminescence quantum yield of powders at 293 K, recorded in air.

d Photoluminescence decay time constants and the associated contributions, at 293 K.

e Average weighted decay lifetime at 293 K.

f Average radiative decay rate constant, *k*_r_ = Φ_PL_/ τ_av_.

g Average nonradiative decay rate constant, *k*_nr_ = (1–Φ_PL_)/ τ_av_.

h Photoluminescence maxima in powder at 77 K.

i Photoluminescence decay time constants and the associated contributions, at 293 K.

j Average weighted decay lifetime at 77 K.

Time-resolved PL spectra reveal additional complexity of the luminescent behavior in powder, which is fully in line with the discussion made so far (Figure 6). At early times, the time-resolved PL spectra of complex **[L1PtCl]­PF**_**6**_ recorded at 293 K resemble its steady-state PL, but as the delay progresses, a weaker and more red-shifted emission is seen to contribute to the PL (Figure 6a). This emission can be attributed to the ^3^MMLCT states distributed over more than two aggregated molecules. **[L2PtCl]­PF**_**6**_ displays a somewhat similar behavior, but with a significantly lesser contribution of the low-energy ^3^MMLCT emissions (Figure 6c). This demonstrates the clear effect of the adamantyl group in suppressing aggregation and restricting the number of associated molecules most likely to only two. Both of these complexes display a rather consistent picture at 77 K with minimal temporal shifts to the PL spectrum (Figure 6b,d). This behavior demonstrates that the PL in this case is dominated by the energetically lowest ^3^MMLCT, *i*.*e*., the largest aggregate. The PL of **[L2PtCl]­PF**_**6**_ **·H**_**2**_ **O** is dominated by its ^3^MLCT+^3^LC emissions evident in the steady-state spectra, but the time-resolved spectra at shorter timescales reveal a contribution from what appears to be ^3^MMLCT emission (Figure 6e,f). This suggests an inhomogeneous composition of the **[L2PtCl]­PF**_**6**_ **·H**_**2**_ **O** sample, containing a small quantity of the anhydrous **[L2PtCl]­PF**_**6**_ form.

**Figure 6:**
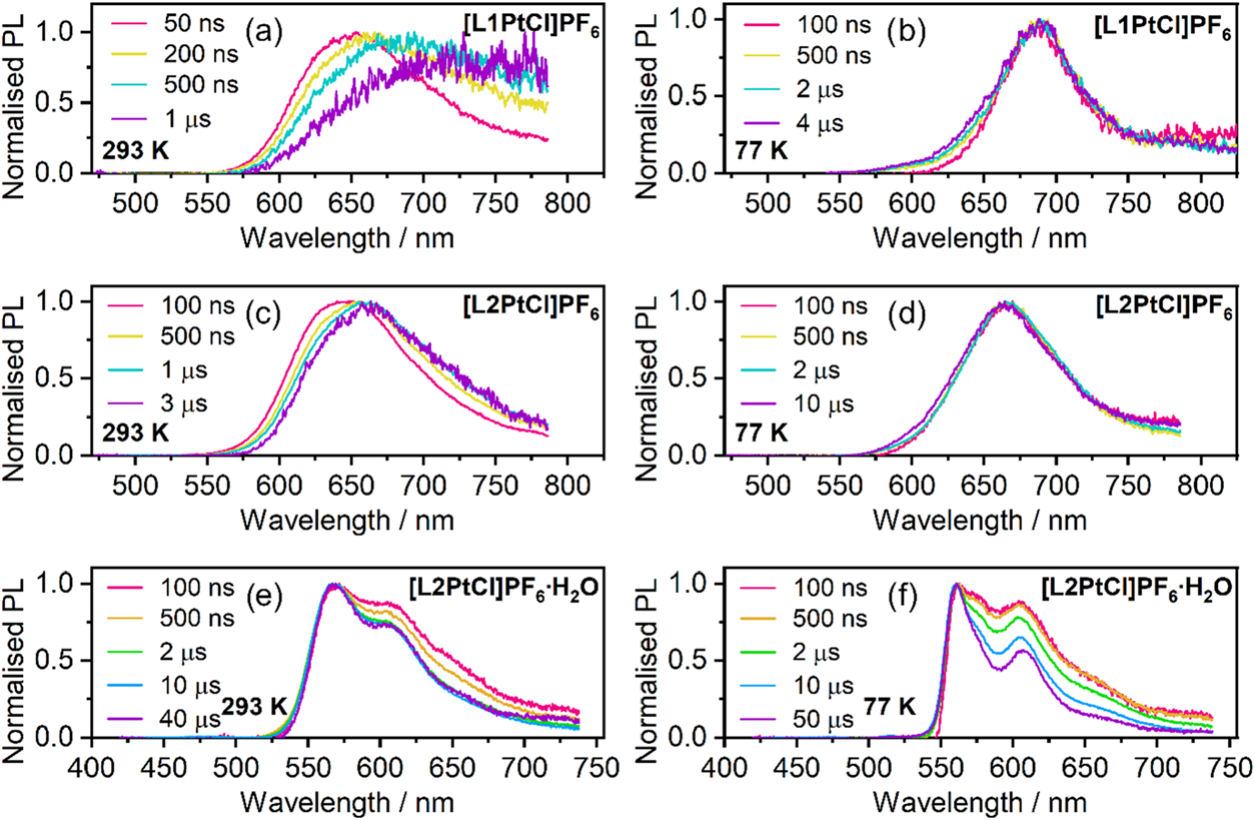
Time-resolved photoluminescence spectra of complexes **[L1PtCl]­PF**_**6**_ (a, b), **[L2PtCl]­PF**_**6**_ (c, d), and **[L2PtCl]­PF**_**6**_ **·H**_**2**_ **O** (e, f) in powders at delay times and temperatures indicated in each figure legend.

### Calculations

To gain a more comprehensive insight into the luminescent properties of monomeric and dimeric forms of **[L1PtCl]­PF**_**6**_, **[L2PtCl]­PF**_**6**_, and **[L2PtCl]­PF**_**6**_ **·H**_**2**_ **O** in the solid state, calculations were carried out using density functional theory (DFT) and time-dependent DFT (TDDFT) implemented in the Orca software package.^48–50^ The single-point energy calculations use the *quasi*-degenerate perturbation theory (QTPD) and involve spin–orbit coupling effects. In our analysis, we neglect the **PF**_**6**_^**‑**^ counterions, optimizing the **[L1PtCl]** ^**+**^ and **[L2PtCl]** ^**+**^ monomeric structures in the ground (S_0_) and triplet excited (T_1_) states. In our simplified analysis, we build a model of **[L1PtCl]**_**2**_^**2+**^ or **[L2PtCl]**_**2**_^**2+**^ dimers by replacing the amide *N*-hydrocarbon groups with R = CH_3_, and the resultant model dimer is labeled as **[L’PtCl]**_**2**_^**2+**^. In this analysis, we were guided by the methodology reported earlier for analogous neutral Pt­(NCN)-X complexes.^41^ Our model **[L’PtCl]**_**2**_^**2+**^ dimer consists of the two monomeric units stabilized through the metallophilic Pt···Pt interaction and assistant π···π interactions. Dimer **[L’PtCl]**_**2**_^**2+**^ defined in this way was optimized without any geometry constraints. Our methodology does not determine whether or not the postulated **[L1PtCl]**_**2**_^**2+**^ or **[L2PtCl]**_**2**_^**2+**^ dimers form—as this is evidenced experimentally—but rather what the excited state properties of these dimers are expected to be.

The S_0_ geometries of the two ions **[L1PtCl]** ^**+**^ and **[L2PtCl]** ^**+**^ are flat around the platinum­(II) center (Figure 7). The two monomeric ions **[L1PtCl]** ^**+**^ and **[L2PtCl]** ^**+**^ display a direct singlet-to-triplet transition (S_0_→T_1_) at 538–540 nm and 534–536 nm, respectively, due to the presence of three *quasi*-degenerate T_1_ sublevels. The transitions display oscillator strength *f*_osc_ 8 × 10^–7^ to 5 × 10^–4^ and 3 × 10^–6^ to 5 × 10^–4^, respectively, and the associated triplet sublevels present a dominating T_1_ character, associated with the HOMO→LUMO transition in each case (Figure 7). The first unequivocally singlet excitations (S_0_→S_n_) are the transitions at 458 and 455 nm, respectively for **[L1PtCl]** ^**+**^ and **[L2PtCl]** ^**+**^. They are assigned a 50.5% S_2_ character in **[L1PtCl]** ^**+**^ and a 41.4% S_2_ and 11.7% S_1_ in **[L2PtCl]** ^**+**^ with *f*_osc_ of 0.037 and 0.0059, respectively. These are associated with the HOMO→LUMO and HOMO→LUMO + HOMO-2→LUMO transitions, respectively, as shown in Figure 7. The HOMO and LUMO present a typical picture, usually seen in analogous complexes of the *NCN* coordination, where the Pt–Cl axis mainly contributes to the HOMO through its d_Pt_ and p_Cl_ orbitals, with the chelating ligand being the main contributor to the LUMO through its π* orbitals. It thus gives a metal-halogen-to-ligand charge transfer (MXLCT) nature to the lowest singlet and triplet excitations. We note that a small contribution of the chelating ligand central pyridine unit indicates an additional partial ligand-centered character.

**Figure 7:**
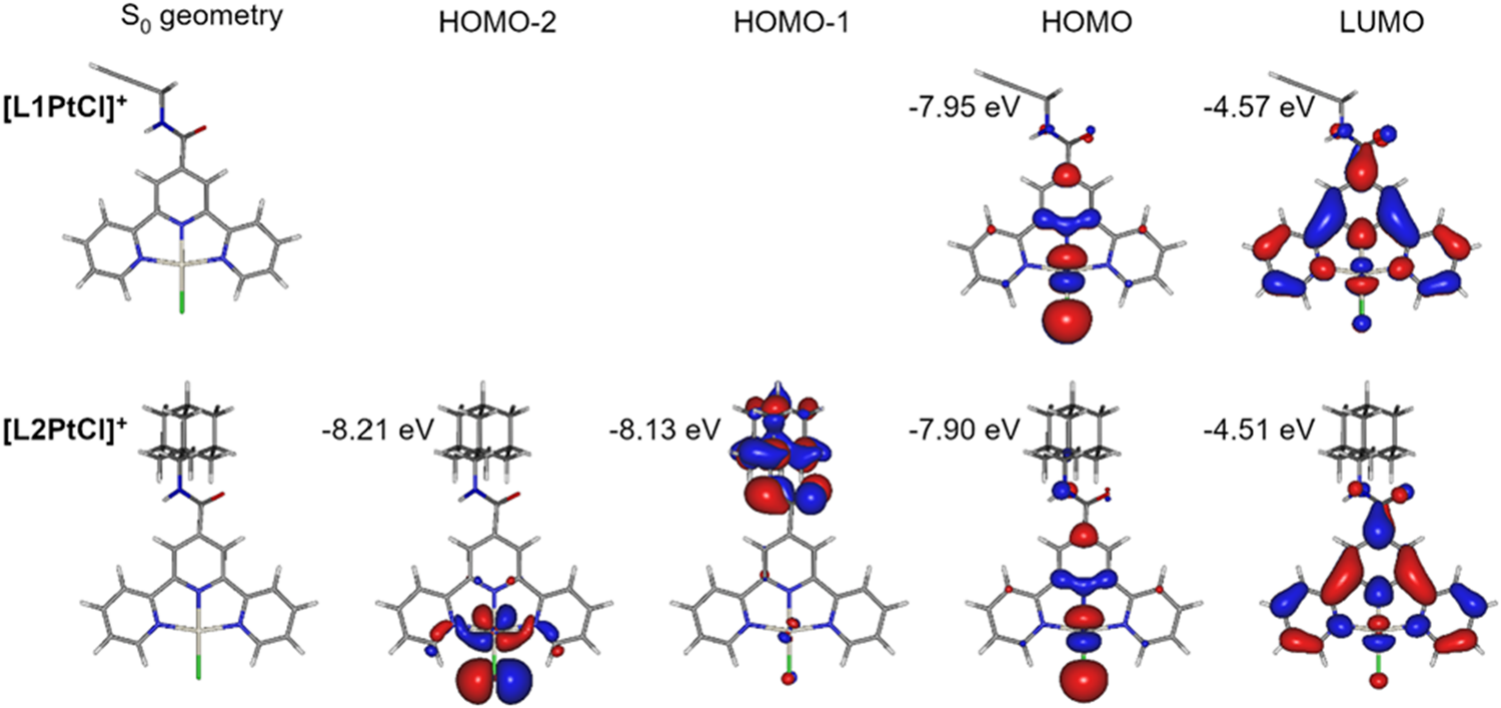
Geometry and selected molecular orbitals of **[L1PtCl]** ^**+**^ and **[L2PtCl]** ^**+**^ at S_0_ geometry rendered with an iso-value of 0.03. Note that the orbitals shown are ones relevant to the discussion.

The T_1_ geometries of the two monomeric ions are alike those of the ground state—effect of the rigidity imposed by the chelating ligand (Figure 8). The energy of the T_1_→S_0_ transitions is at 583–585 nm (17,095–17,148 cm^–1^ or 2.12–2.13 eV) and 574–576 nm (17,378–17,425 cm^–1^ or 2.15–2.16 eV), while the zero-field splitting of T_1_ is at 53.1 cm^–1^ (6.6 meV) and 47.4 cm^–1^ (5.9 meV), respectively, for **[L1PtCl]** ^**+**^ and **[L2PtCl]** ^**+**^. In both cases, the T_1_ state involves a HOMO→LUMO transition. Here, the picture of the orbitals is generally similar to that at the S_0_ state, at least in **[L1PtCl]** ^**+**^, where the nature of the T_1_ state is unequivocally of the ^3^MXLCT character with a ligand-centered contribution. Somewhat surprisingly, the adamantyl-containing fragment clearly contributes to the HOMO in **[L2PtCl]** ^**+**^ (Figure 8), but the contribution of the Pt–Cl group is seemingly not observed. A closer analysis of the same orbital, however, printed with an iso-value of 0.01 (Figure S37) reveals a small contribution of the aforementioned Pt–Cl moiety. An orbital of a similar geometry to the HOMO is also observed at the S_0_ geometry as HOMO-1 (Figure 7), and it appears that the HOMO and HOMO-1 from the said S_0_ geometry swap places at the T_1_ geometry. This orbital configuration gives a more interligand charge transfer nature of the T_1_ state in **[L2PtCl]** ^**+**^. While unexpected, it appears that the involvement of the adamantane unit as a donor is overall not entirely surprising, and earlier computational research suggests the HOMO energy of charge-neutral adamantane to be similar to the HOMO energy calculated in **[L2PtCl]** ^**+**^.^51^ We calculated the radiative decay rate constant *k*_r_ and natural decay lifetime τ_0_ = *k*_r_^–1^ of **[L1PtCl]** ^**+**^ and **[L2PtCl]** ^**+**^ at *k*_r_ = 6 × 10^4^ s^–1^, τ_0_ = 16 μs and *k*_r_ = 9 × 10^4^ s^–1^, τ_0_ = 11 μs, respectively. These are typical values for a mononuclear platinum­(II) complex. Emissions of a similar order of lifetime are observed in **[L2PtCl]­PF**_**6**_ **·H**_**2**_ **O**, with a particularly good agreement between the calculated and experimental *k*_r_ values, while the calculated energy of the T_1_→S_0_ transitions also agrees with that observed experimentally. This further confirms the emission in **[L2PtCl]­PF**_**6**_ **·H**_**2**_ **O** indeed originates from the monomeric **[L2PtCl]** ^**+**^ ions.

**Figure 8:**
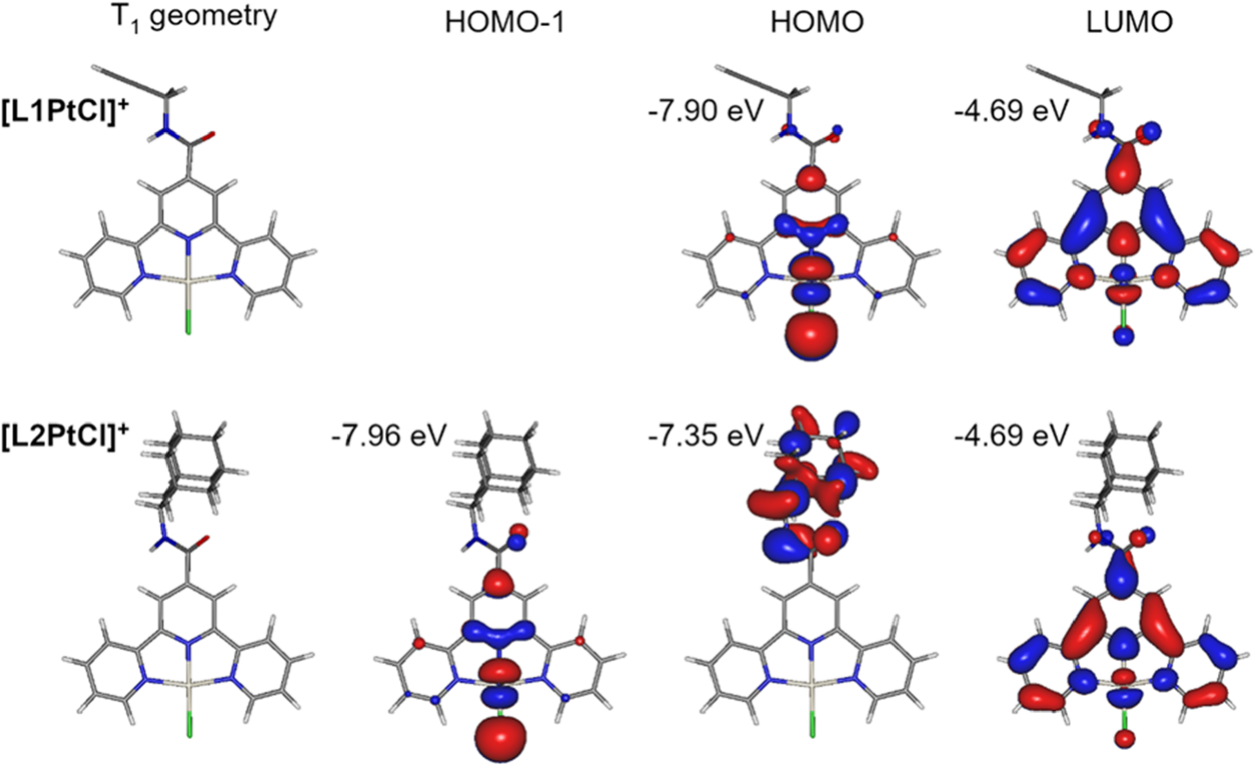
Geometry and selected molecular orbitals of **[L1PtCl]** ^**+**^ and **[L2PtCl]** ^**+**^ at T_1_ geometry rendered with an iso-value of 0.03. Note that the orbitals shown are ones relevant to the discussion.

The dimeric form of the complex (**[L’PtCl]**_**2**_^**2+**^) in the T_1_ state is composed of two coplanar **[L’PtCl]** ^**+**^ units in a head-to-tail arrangement^41^ (Figure 9), with the C­(=O)–NH–CH_3_ groups pointing away from each other. The calculated Pt···Pt distance is 3.16 Å, and the Cl–Pt–Pt’–Cl’ dihedral angle is 141.9°. The energy of the T_1_→S_0_ transition in this case is 719–728 nm (13743–13914 cm^–1^ or 1.70–1.73 eV), while the zero-field splitting of T_1_ is at 171 cm^–1^ (21 meV). T_1_ excited state is associated with the HOMO→LUMO transition. The HOMO is localized on the antibonding MO formed from mixing the d_z2_ orbitals of the Pt­(II) centers and the p_z_ orbitals of the auxiliary chlorides, while the LUMO has a mixed ligand π* + metal d_xz_ character. This picture is consistent with that observed for the related Pt­(NCN)-Cl complexes,^41^ with the T_1_ state being of the ^3^MMLCT (metal-metal-to-ligand charge transfer) nature. The calculated *k*_r_ and τ_0_ are 7 × 10^5^ s^–1^ and 1.4 μs, respectively, which agree well with the experimental data. Calculations suggest that the radiative lifetimes of the aggregates are shorter than those of the monomeric forms. This effect is likely partly due to a larger *k*_r_, as shown above, but inevitably also a larger nonradiative decay rate constant *k*_nr_ in aggregates due to the energy gap law.^52^

**Figure 9:**
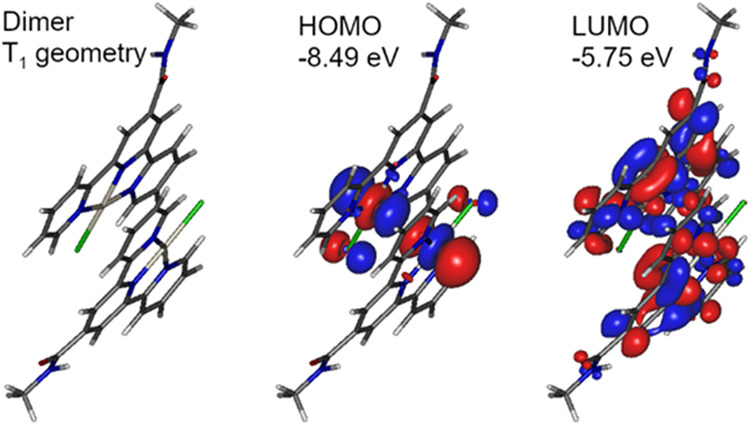
Geometry and frontier molecular orbitals of the **[L’PtCl]**_**2**_^**2+**^ dimer at T_1_ geometry. Orbitals rendered using an iso-value of 0.03.

### Antiviral Activity against HCoV-OC43 (*Coronaviridae*) and IAV (*Orthomyxoviridae*) Viruses

The antiviral activity of **[L1PtCl]­PF**_**6**_ and **[L2PtCl]­PF**_**6**_ was evaluated in rhabdomyosarcoma (RD) cells infected with the human coronavirus OC43 (HCoV-OC43), which represents a surrogate for the highly pathogenic SARS-CoV-2 coronavirus (Table 2 and Figure 10). The platinum-free **L1**, **L2**, and 1-adamantanemethylamine were used as references, and the antiviral drug remdesivir served as a positive control. The antiviral activity was expressed as the IC_50_ value, and the cytotoxicity of the compounds against the RD host cells was expressed as the CC_50_ value. Both **[L1PtCl]­PF**_**6**_ and **[L2PtCl]­PF**_**6**_ efficiently inhibit viral replication in the micromolar concentration range with **[L2PtCl]­PF**_**6**_ showing approximately 4-fold stronger activity than **[L1PtCl]­PF**_**6**_. Importantly, the CC_50_ values for the cytotoxicity of the two platinum complexes are much higher than their IC_50_ values for the antiviral activity, confirming that the antiviral effects of the lower dosages do not overlap with toxic effects against the host cells (Figure 10). However, the respective free ligands, **L1** and **L2**, triggered stronger antiviral activity than the respective platinum complexes, indicating that the observed effects are mostly related to the tpy ligand system. In this context, it is of interest to note that the tpy ligand with the adamantane moiety **L2** was significantly more active than 1-adamantanemethylamine without tpy.

**Figure 10:**
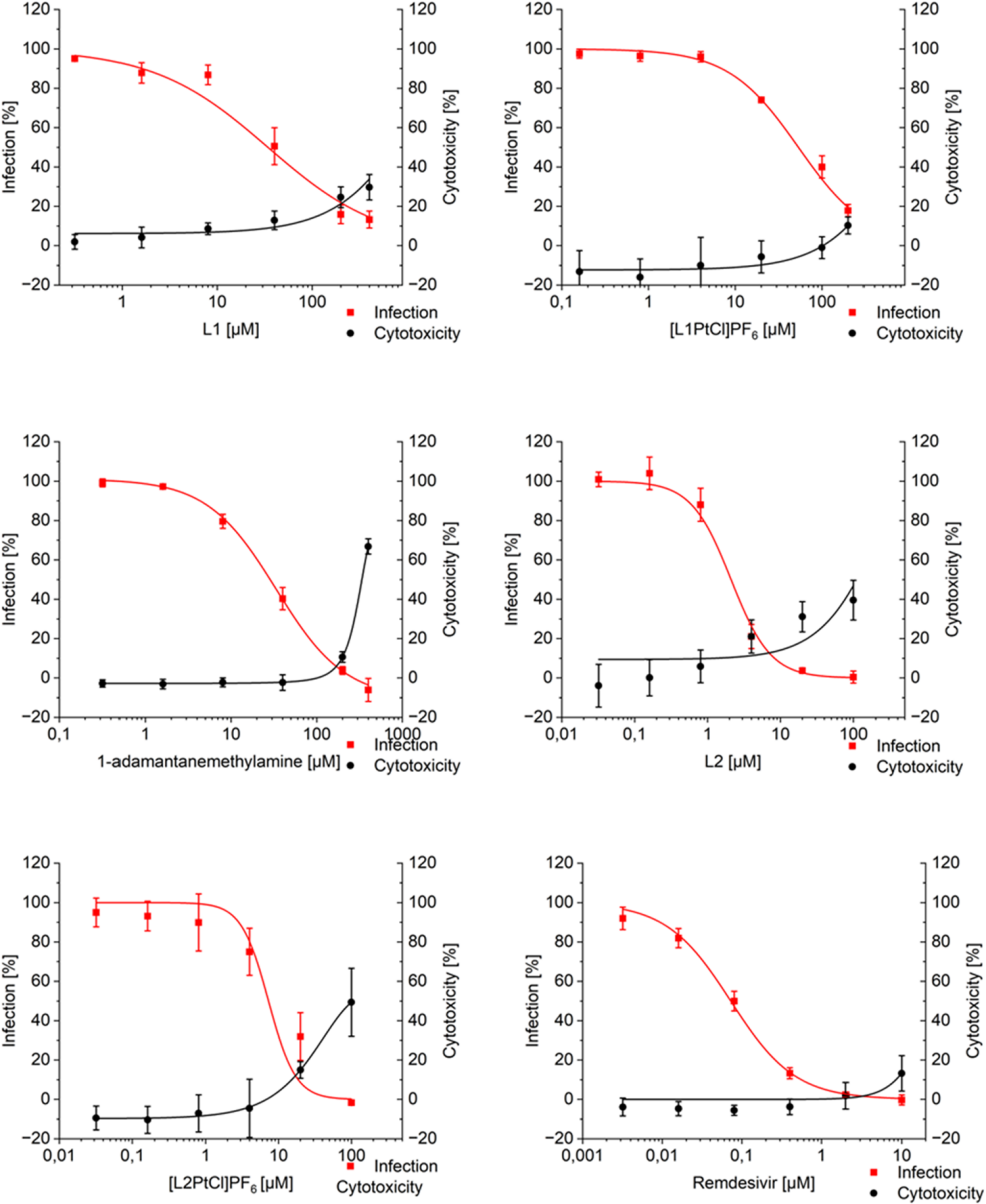
Antiviral activity and cytotoxicity of **L1**, **L2**, **[L1PtCl]­PF**_**6**_, **[L2PtCl]­PF**_**6**_, and remdesivir in HCoV–OC43-infected RD cells (MOI = 0.1, *n* = 3–4).

**Table 2:** Antiviral Activity (IC_50_) and Cytotoxic Effect (CC_50_) of Test Compounds in RD Cells Infected with HCoV–OC43 at an MOI (Multiplicity of Infection) of 0.1^a^

**compound**	IC_50_ calc. [μM]	CC_50_ calc. [μM]
**L1**	39 ± 12	>400
**[L1PtCl]PF**_**6**_	50 ± 5	>100
**1–adamantanemethylamine**	30 ± 6	332 ± 7
**L2**	2 ± 0.5	>100
**[L2PtCl]PF**_**6**_	12 ± 7	74 ± 11
**remdesivir**	0.09 ± 0.02	>10

a Results are presented as mean values ± standard deviation of IC_50_ or CC_50_ values from 3 to 4 independent experiments.

In the following, our investigation focused on the impact of **L1**, **L2**, **[L1PtCl]­PF**_**6**_, and **[L2PtCl]­PF**_**6**_ compounds and the rimantadine reference drug on the replication of the replication-competent influenza virus A/Puerto Rico/8/34 in A549 cells (Table 3 and Figure 11). The IAV belongs to the *Orthomyxoviridae* virus family and has a different replication mechanism from coronaviruses such as HCoV–OC43 and SARS–CoV–2. All studied compounds and rimantadine have shown cytotoxic activity against the tested lung adenocarcinoma cells. The most toxic were ligands **L2** and **L1**. The ligands were also potent IAV replication inhibitors. However, this desirable activity was overwhelmed by the cytotoxic effect. Coordination of the Pt center resulted in a decrease of cytotoxicity and virus replication inhibitory activity alike. Though, adamantyl complex **[L2PtCl]­PF**_**6**_ has remained a very potent IAV inhibitor with an inhibitory IC_50_ of 2 ± 1 μM. Furthermore, the selectivity index (SI) for **[L2PtCl]­PF**_**6**_ was higher than that of the rimantadine drug (Table 3). On the contrary, **[L1PtCl]­PF**_**6**_ complex failed to show any desirable anti-IAV activity and its SI was 1. This substantiates the important role of the bulky adamantyl group in **[L2PtCl]­PF**_**6**_ to exert antiviral activity.

**Figure 11:**
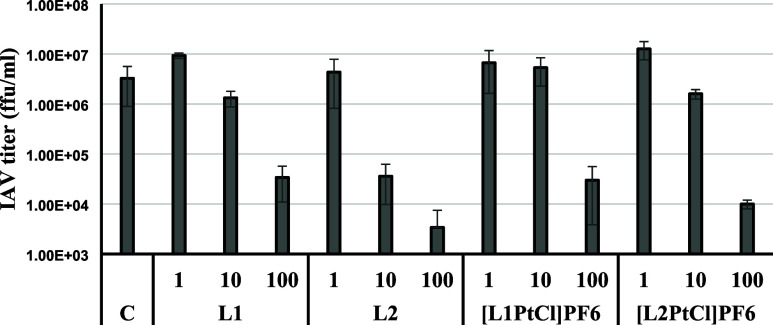
Antiviral activity of **L1**, **L2**, **[L1PtCl]­PF**_**6**_, and **[L2PtCl]­PF**_**6**_ in IAV-infected A549 cells (MOI = 0.01). The virus titers in the supernatant were analyzed at 48 h postinfection using the focus formation assay (FFU). Results are presented as mean values ± standard deviation.

**Table 3:** Antiviral Activity (IC_50_) and Cytotoxic Effect (CC_50_) of Test Compounds in A549 Cells Infected with Influenza Virus A/Puerto Rico/8/34 in A549 Cells at an MOI of 0.01 and Then Treated with Increasing Concentrations of Synthesized Compounds^a^

**compound**	IC_50_ calc. [μM]	CC_50_ calc. [μM]	**SI**
**L1**	3 ± 1	17 ± 8	5
**[L1PtCl]PF**_**6**_	37 ± 2	37 ± 6	1
**L2**	0.58 ± 0.03	5.62 ± 0.01	7
**[L2PtCl]PF**_**6**_	2 ± 1	22.3 ± 0.5	11
**rimantadine**	9.6 ± 0.2	68 ± 14	7

a Results are presented as mean values ± standard deviation.

### SARS-CoV-2 M^pro^ and Human TMPRSS2 Inhibition

The antiviral activity of ligands and platinum complexes was further studied in respect to their inhibitory activity against SARS-CoV-2 3C-like or “Main Protease” (3CL or M^Pro^ or nsp5) and a human type II transmembrane serine protease TMPRSS2. Both enzymes are important for the replication cycle of the SARS-CoV-2 particles in human cells.^53^ We note that inhibitory activity studies of Pt­(II)­(tpy) complexes toward SARS-CoV-2 proteases (PL^pro^ and M^pro^) are rather scarce.^38^ Inhibitory activity of **L1**, **L2**, and complexes **[L1PtCl]­PF**_**6**_ and **[L2PtCl]­PF**_**6**_ toward M^pro^ and TMPRSS2 is shown in Figure 12. Ligands **L1** and **L2** were weaker inhibitors than their corresponding complexes **[L1PtCl]­PF**_**6**_ and **[L2PtCl]­PF**_**6**_. The inhibitory activity of **L1** was the weakest of all compounds with the IC_50_ > 100 μM, while **L2** showed modest activity (IC_50_ ≈ 25 μM). The complexation of platinum to ligands **L1** and **L2** results in an increase of M^pro^ inhibitory activity. The dose-dependent inhibition of M^pro^ activity was observed for complex **[L1PtCl]­PF**_**6**_, which is characterized by an IC_50_ of 8 μM. Inhibitory activity of complex **[L2PtCl]­PF**_**6**_ was comparable to that of **[L1PtCl]­PF**_**6**_, with the IC_50_ ≈ 1 μM. On a separate note, ligands **L1** and **L2** did not have any effect on the human serine protease TMPRSS2 activity. Only the highest doses of complexes **[L1PtCl]­PF**_**6**_ and **[L2PtCl]­PF**_**6**_ slightly decreased the TMPRSS2 activity. In conclusion, this suggests that the observed inhibitory activity of these complexes is specific to the cysteine protease M^pro^.

**Figure 12:**
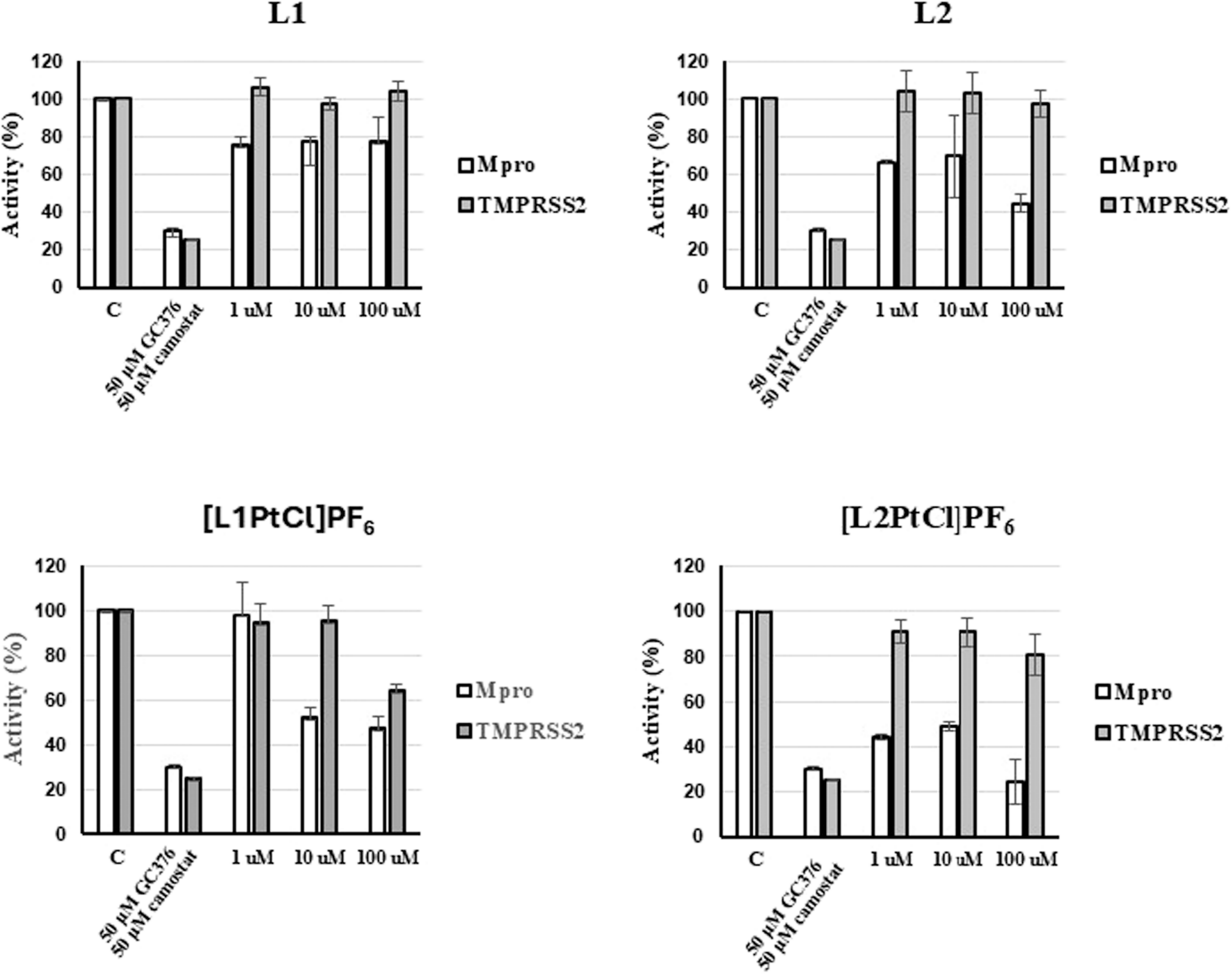
Inhibitory activity of **L1**, **L2**, **[L1PtCl]­PF**_**6**_, and **[L2PtCl]­PF**_**6**_ toward M^pro^ and TMPRSS2. C refers to negative control (DMSO). 50 μM GC376 and 50 μM camostat refer to the positive control for M^pro^ and TMPRSS2, respectively. The enzymatic activities were determined after 2 h of incubation.

## Conclusions

In conclusion, two novel Pt­(II) tpy complexes **[L1PtCl]­PF**_**6**_ and **[L2PtCl]­PF**_**6**_, photoluminescent in the solid state, were obtained and characterized. These complexes differ in the bulkiness of substituents in position 4′ of the tpy pincer ligand L: **L1**—propargyl; **L2**—adamantyl. Photoluminescent properties of **[L1PtCl]­PF**_**6**_ and **[L2PtCl]­PF**_**6**_ in powders were examined with steady-state and time-resolved spectroscopy at 293 and 77 K. Measurements were supported by (TD)­DFT calculations. At 293 K, photoluminescence spectra of both complexes are virtually the same and characterized by the broadband unstructured orange-red ^3^MMLCT emission (λ_PL_ ≈ 650 nm). This is an indication of short Pt···Pt contacts between individual molecules of studied complexes in their powder samples. Contrary to 293 K, at 77 K, the photoluminescence spectra of **[L1PtCl]­PF**_**6**_ and **[L2PtCl]­PF**_**6**_ are significantly different. The former complex emits at 688 nm while the latter at 665 nm. This can be rationalized by the presence of larger-than-dimer aggregates in the powder sample of **[L1PtCl]­PF**_**6**_, whereas the bulky adamantyl group in **[L2PtCl]­PF**_**6**_ is found to prevent the formation of these multimolecular species. At lower temperatures, these larger aggregates act as traps, leading to them dominating the PL in **[L1PtCl]­PF**_**6**_, instead of the dimers, and leading to the observed visibly red-shifted photoluminescence.

Herein, we also report on the **[L2PtCl]­PF**_**6**_ **·H**_**2**_ **O** solvate. Notably, a similar solvate was not obtained for the complex with the less bulky **L1** ligand. Contrary to the behavior of the anhydrous **[L2PtCl]­PF**_**6**_, the photoluminescence spectra of **[L2PtCl]­PF**_**6**_ **·H**_**2**_ **O** powder, both at 293 and 77 K, feature a vibronically resolved yellow emission, λ_PL_ = 567 and 560 nm, respectively, with an ^3^MLCT+ ^3^LC character. Such photoluminescence characteristics indicate significant relaxation of the Pt···Pt contacts in **[L2PtCl]­PF**_**6**_ **·H**_**2**_ **O** caused by water molecules within the crystal structure.

In this account, we also report on **[L1PtCl]­PF**_**6**_ and **[L2PtCl]­PF**_**6**_ activity against HCoV-OC43 (surrogate of SARS-CoV-2) and IAVs. Adamantyl complex **[L2PtCl]­PF**_**6**_ shows superior HCoV-OC43 and IAV antiviral activity with IC_50_ values of 12 ± 7 and 2 ± 1 μM to its propargyl congener **[L1PtCl]­PF**_**6**_, which showed the IC_50_ of 50 ± 5 and 37 ± 2 μM, respectively. Unfortunately, both platinum complexes were also toxic in the A549 cancer cells. Despite that undesirable effect, the selectivity index of **[L2PtCl]­PF**_**6**_ toward influenza virus inhibition was higher than that of the rimantadine reference drug. We further assessed the inhibitory activity of both ligands and complexes against M^pro^ cysteine protease of SARS-CoV-2 and the human TMPRSS2 serine protease, both of which are key for the replication of coronavirus in human cells. These assays showed the ligands are weak M^pro^ inhibitors. In contrast, both the complexes have shown inhibitory activity characterized by a low (IC_50_ < 10 μM) inhibitory concentration. Collectively, antiviral and enzymatic studies point toward that mechanism of action for the studied compounds is virus-type dependent. In respect to HCoV–OC43 coronavirus antiviral activity, **[L1PtCl]­PF**_**6**_ and **[L2PtCl]­PF**_**6**_ complexes act as M^pro^ inhibitors. The mechanism of the HCoV–OC43 antiviral activity of **L1** nad **L2** remains elusive although we hypothesized that it can be linked to Zn^2+^ chelation in PL^pro^. On the other hand, the IAV replication inhibition by **[L2PtCl]­PF**_**6**_ is likely due to the inhibition of IAV matrix 2 (M2) protein by the adamantyl portion of the complex.

To sum up, our results emphasize the crucial role of ligand bulkiness in the design of novel solid-state photoluminescent Pt­(II) tpy emitters and the intricate interplay between different antiviral mechanisms of antiviral action that are observed for these complexes. Further and more in-depth studies are required to fully decipher these antiviral activity mechanisms.

## Experimental

### General Considerations

All reactions were carried out using standard Schlenk techniques. Solvents were of reagent grade and were used without prior purification. Propargylamine, 1-adamantanemethylamine, *N,N*′-diisopropylcarbodiimide, *N*-hydroxysuccinimide, *cis*-[PtCl_2_(COD)], NH_4_PF_6_, and triethylamine were purchased from commercial suppliers and used without further purification. 2,2′:6′,2″-Terpyridine-4′-carboxylic acid was synthesized according to the literature.^40^ Yields refer to the isolated products. The NMR spectra were recorded on a Bruker AV600 Kryo (600 MHz) spectrometer. Chemical shifts δ are reported in ppm using residual DMSO (^1^H δ = 2.50 ppm and ^13^C δ = 39.52 ppm) as reference. Mass spectra were recorded using the electrospray ionization MS method on a Synapt G2-Si mass spectrometer (Waters). High-resolution mass spectrometry (HRMS) measurements were carried out using a Synapt G2-Si mass spectrometer (Waters) equipped with an ESI source and quadrupole-time-of-flight mass analyzer. The mass spectrometer was operated in the positive ion detection mode. The measurements were performed with a capillary voltage set to 2.7 kV and a sampling cone to 20 V. The source temperature was 110 °C. The results of the measurements were processed using the MassLynx 4.1 software (Waters) incorporated in the instrument. TGA measurements were performed with a Discovery TGA 5500 (TA Instruments) under N_2_ atmosphere (gas flow: 10 mL/min) up to 600 °C (temp. gradient: 10 °C/min). IR spectra were recorded on an FTIR Nexus Nicolet apparatus. Microanalyses were performed by the Analytical Services of the Polish Academy of Sciences (Łódź).

#### Synthesis of Terpyridine Ligands **L1** and **L2**

*N,N*′-Diisopropylcarbodiimide (139 mg, 170 μL, 1.1 mmol) was added dropwise to a stirred solution of 2,2′:6′2″-terpyridine-4′-carboxylic acid (**tpyCOOH**) (250 mg, 0.9 mmol) and *N*-hydroxysuccinimide (127 mg, 1.1 mmol) in a 15 mL DMF/DCM (4:1, v/v) mixture. The resulting reaction mixture was stirred for 30 min at ambient temperature. Then, the propargylamine (50 mg, 58 μL, 0.9 mmol) or 1-adamantanemethylamine (149 mg, 0.9 mmol) followed by triethylamine (111 mg, 153 μL, 1.1 mmol) were added. The thus formed reaction mixture was kept stirred at ambient temperature overnight. Then, it was poured into 1 M HCl_(aq)_ and extracted with DCM. The water layer was treated with saturated NaHCO_3(aq)_. Precipitated material was filtered, washed with water, and dried overnight under vacuum. Ligand **L1** was obtained as a white solid in 91% (257 mg) yield. ^1^H NMR (600 MHz, DMSO-d_6_): δ = 9.56 (t, *J*_HH_ = 5.4 Hz, 1H, NH), 8.83 (s, 2H, H_3′,5′_ tpy), 8.76 (d, *J*_HH_ = 4.2 Hz, 2H, H_6,6″_ tpy), 8.64 (d, *J*_HH_ = 7.8 Hz, 2H, H_3,3″_ tpy), 8.03 (td, *J*_HH_ = 7.8, 1.8 Hz, 2H, H_4,4″_ tpy), 7.53 (ddd, *J*_HH_ = 7.2, 5.0, 0.5 Hz, 2H, H_5,5″_ tpy), 4.13 (dd, *J*_HH_ = 5.4, 2.4 Hz, 2H, CH_2_), 3.17 (t, *J*_HH_ = 2.4 Hz, 1H, C≡CH) ppm. ^13^C­{^1^H} NMR (150 MHz, DMSO-d_6_): δ = 164.4, 155.7, 154.4, 149.3, 143.4, 137.4, 124.6, 120.9, 118.2, 80.8, 73.0, 28.7 ppm. MS (TOF ESI+): *m*/*z* = 315.1251 (M + H^+^) (calcd for C_19_H_15_N_4_O: 315.1246). FTIR (KBr ν [cm^–1^]): 3051, 3015, 2945, 2894, 2853, 2125 (C≡CH), 1635 (C = O), 1586, 1536, 1467, 1395, 1283, 1079, 993, 895. Anal. calcd for C_19_H_14_N_4_O: C, 72.60; H, 4.49; N, 17.82%. Found: C, 72.35; H, 4.46; N, 17.81%.

Ligand **L2** was obtained as a white solid in 83% (317 mg) yield. ^1^H NMR (600 MHz, DMSO-d_6_): δ = 8.92 (bt, *J*_HH_ = 6.0 Hz, 1H, NH), 8.81 (s, 2H, H_3′,5′_ tpy), 8.77 (dm, *J*_HH_ = 4.2 Hz, 2H, H_6,6″_ tpy), 8.65 (bd, *J*_HH_ = 7.8 Hz, 2H, H_3,3″_ tpy), 8.04 (td, *J*_HH_ = 7.8, 1.8 Hz, 2H, H_4,4″_ tpy), 7.54 (ddd, *J*_HH_ = 7.2, 4.8, 1.2 Hz, 2H, H_5,5″_ tpy), 3.06 (d, *J*_HH_ = 6.6 Hz, 2H, CH_2_), 1.95 (bs, 3H, 1-ada), 1.67 (d, *J*_HH_ = 12.0 Hz, 3H, 1-ada), 1.61 (d, *J*_HH_ = 12.0 Hz, 3H, 1-ada), 1.54 (d, *J*_HH_ = 2.4 Hz, 6H, 1-ada) ppm. ^13^C­{^1^H} NMR (150 MHz, DMSO-d_6_): δ = 165.2, 155.5, 154.6, 149.3, 144.6, 137.4, 124.6, 120.9, 119.4, 50.8, 36.5, 34.4, 27.7 ppm. MS (TOF ESI+): *m*/*z* = 425.2340 (M+H^+^) (calcd for C_27_H_29_N_4_O: 425.2341). FTIR (KBr ν [cm^–1^]): 3318, 3087, 3064, 2899, 2847, 1643, 1586, 1543, 14657, 1395, 1282. Anal. calcd for C_27_H_28_N_4_O: C, 76.39; H, 6.65; N, 13.20%. Found: C, 76.41; H, 6.63; N, 13.22%.

#### Synthesis of Complexes **[L1PtCl]­PF**_**6**_, **[L2PtCl]­PF**_**6**_, and **[L2PtCl]­PF**_**6**_ **·H**_**2**_ **O**

A suspension of *cis*-[PtCl_2_(COD)] (251 mg, 0.67 mmol) and **L1** (210 mg, 0.67 mmol) or **L2** (284 mg, 0.67 mmol) in a water/DMF (3 mL/17 mL) mixture was heated at 65 °C for 15 min with stirring. Then, the reaction mixture was poured into water and extracted with DCM. Resulted layers were separated, and the water layer was treated with NH_4_PF_6_, agitated, and kept at 4 °C overnight. Resulted dark red (**[L1PtCl]­PF**_**6**_) or yellow (**[L2PtCl]­PF**_**6**_ **·H**_**2**_ **O**) precipitates were centrifuged, filtered, washed with water, and dried under vacuum. Complex **[L2PtCl]­PF**_**6**_ was isolated from the water layer at ambient temperature immediately after its precipitation after the addition of NH_4_PF_6_ and centrifugation. It forms an orange crystalline solid.

Complex **[L1PtCl]­PF**_**6**_ was obtained as a red solid in 86% (397 mg) yield. ^1^H NMR (600 MHz, DMSO-d_6_): δ = 9.45 (t, *J*_HH_ = 5.4 Hz, 1H, NH), 8.75 (s, 2H, H_3′,5′_ tpy), 8.49 (d, *J*_HH_ = 8.1 Hz, 2H, H_3,3″_ tpy), 8.45 (d, *J*_HH_ = 4.8 Hz, 2H, H_6,6″_ tpy), 8.40 (t, *J*_HH_ = 7.8 Hz, 2H, H_4,4″_ tpy), 7.75 (t, *J*_HH_ = 6.6 Hz, 2H, H_5,5″_ tpy), 4.24 (dd, *J*_HH_ = 5.4, 2.4 Hz, 2H, CH_2_), 3.35 (bs, 1H, C≡CH) ppm. ^13^C­{^1^H} NMR (150 MHz, DMSO-d_6_): δ = 162.0, 157.3, 154.4, 150.9, 145.3, 142.7, 129.4, 126.1, 122.5, 79.9, 74.1, 29.1 ppm. MS (TOF ESI+): *m*/*z* = 544.0507 (M^+^) (calcd for C_19_H_14_N_4_OClPt: 544.0504). FTIR (KBr ν [cm^–1^]): 3107, 3062, 2939, 2888, 2121­(C≡CH), 1671­(C = O), 1610, 1536, 1478, 1423, 1296, 845. Anal. calcd for C_19_H_14_ClF_6_N_4_OPPt: C, 33.08; H, 2.05; N, 8.12%. Found: C, 33.19; H, 2.18; N, 8.17%.

Complex **[L2PtCl]­PF**_**6**_ was obtained as an orange solid in 65% (348 mg) yield. Solvate **[L2PtCl]­PF**_**6**_ **·H**_**2**_ **O** was obtained as a yellow solid in 51% (279 mg) yield. Complex **[L2PtCl]­PF**_**6**_ and solvate **[L2PtCl]­PF**_**6**_ **·H**_**2**_ **O** gave virtually the same NMR and HRMS spectra. To illustrate that, Figures S29 and S30 show HRMS spectra of **[L2PtCl]­PF**_**6**_ and **[L2PtCl]­PF**_**6**_ **·H**_**2**_ **O**, respectively. ^1^H NMR (600 MHz, DMSO-d_6_): δ = 8.91­(bd, *J*_HH_ = 5.4 Hz, 2H, H_6,6″_ tpy), 8.80 (s, 2H, H_3′,5′_ tpy), 8.87­(t, *J*_HH_ = 6.0 Hz, 1H, NH), 8.69 (d, *J*_HH_ = 7.8 Hz, 2H, H_3,3″_ tpy), 8.53 (t, *J*_HH_ = 7.8 Hz, 2H, H_4,4″_ tpy), 7.97 (t, *J*_HH_ = 6.6 Hz, 2H, H_5,5″_ tpy), 3.13 (d, *J*_HH_ = 6.0 Hz, 2H, CH_2_), 1.98 (bs, 3H, 1-ada), 1.69 (d, *J*_HH_ = 12.0 Hz, 3H, 1-ada), 1.64 (d, *J*_HH_ = 12.0 Hz, 3H, 1-ada), 1.58 (s, 6H, 1-ada) ppm. ^13^C­{^1^H} NMR (150 MHz, DMSO-d_6_): δ = 163.1, 157.8, 154.6, 151.3, 146.5, 142.7, 129.4, 126.1, 122.5, 51.2, 36.4, 34.3, 27.7 ppm. MS (TOF ESI+): *m*/*z* = 654.1598 (M^+^) (calcd for C_27_H_28_N_4_OClPt: 654.1599). FTIR (KBr ν [cm^–1^]): 3081, 2902, 2847, 1666, 1616, 1538, 1479, 1424, 1294, 847. Anal. Calcd for C_27_H_28_ClF_6_N_4_OPPt: C, 40.53; H, 3.53; N, 7.00%. Found: C, 40.66; H, 3.72; N, 7.09%. Anal. calcd for C_27_H_28_ClF_6_N_4_OPPt + H_2_O: C, 39.64; H, 3.70; N, 6.85%. Found: C, 39.61; H, 3.68; N, 6.83%.

### TG Analysis

The TG curves of **[L2PtCl]­PF**_**6**_ **·H**_**2**_ **O** are shown in Figures S31 and S32. Hydrate loses water in the temperature range of 25–75 °C (the maximum weight loss was at *ca*. 33 °C). At this temperature range, weight loss of 1.912% (calcd 2.188%) occurred, which corresponds to water release. The final thermal decomposition of the product occurred at 357–407 °C.

### X-Ray Structure Analysis

Good quality single crystals of **L1** and **L2** were selected for the X-ray diffraction experiments at *T* = 100(2) K. Crystals were mounted with Paratone-N oil using the Hampton Research CryoLoops. Diffraction data were collected on the Agilent Technologies SuperNova Dual Source diffractometer with Cu Kα radiation (λ = 1.54184 Å) using CrysAlis Pro software.^54^ In both cases, the multiscan empirical absorption correction using spherical harmonics, implemented in SCALE3 ABSPACK scaling algorithm, was applied. The structural determination procedure was carried out using the SHELX package. The structures were solved with an intrinsic phasing method using the SHELXT program,^55^ and then successive least-squares refinement was carried out based on the full-matrix least-squares method on *F* ^2^ using the SHELXL program.^56^

The positions of amine hydrogen atoms were located on a difference Fourier map. The remaining H atoms were positioned geometrically and refined using fixed *U*_iso_(H) parameters. All molecular interactions in crystals of the investigated compounds were identified using the PLATON program.^57^ The figures for this publication were prepared using the Olex2 and Mercury programs.^58,59^

### Optical Spectroscopy

Absorption spectra in solution were recorded with a UV-3600 (Shimadzu) double-beam spectrophotometer, while excitation spectra of powders were recorded using a Horiba Fluoromax spectrofluorometer. Photoluminescence quantum yields in powders were obtained by using an integrating sphere integrated with the FS5 Edinburgh Instruments spectrofluorometer. Photoluminescence decays and spectra in powder were recorded using nanosecond-gated luminescence and lifetime measurements (from 400 ps to 1 s) using the third harmonic (355 nm) of a high-energy pulsed Nd:YAG laser (EKSPLA). The emitted light was focused onto a spectrograph and detected with a sensitive gated iCCD camera (Stanford Computer Optics) having subnanosecond resolution. Time-resolved measurements were performed by exponentially increasing gate and integration times. Further details are available in an earlier work.^60^ Temperature-dependent experiments were conducted using a liquid nitrogen cryostat VNF-100 (sample in flowing vapor, Janis Research) under a nitrogen atmosphere.

### PXRD

Patterns were recorded at room temperature on a Bruker D8 Advance diffractometer equipped with a LYNXEYE detector using Cu Kα radiation (λ = 1.5418 Å) in the Bragg–Brentano (θ/2θ) horizontal geometry (flat reflection mode) in continuous scan mode with 0.025° steps. For each sample, the Sherrer equation was applied to 5 isolated reflexes, with the *K* value set to 0.94.

### Calculations

To assist in the interpretation of the experimental results, we have performed density functional theory (DFT) and time-dependent density functional theory (TDDFT) simulations using the ORCA 5.0.3 quantum chemistry software.^48–50,61^ All molecular orbital (MO) iso surfaces were visualized using Gabedit 2.5.0 ^62^ or Avogadro 1.2.0.^63,64^ Geometry optimizations in the ground state (S_0_) and triplet excited state (T_1_) were performed at the BP86/def2-SVP^65,66^ level of theory for dimeric forms (only T_1_) and BP86/def2-TZVP^66^ for monomeric forms, and respective def2-SVP/C,^67^ def2-TZVP/C,^68^ and def2/J^68^ auxiliary basis sets were used in each case. We also used atom-pairwise dispersion correction with the Becke–Johnson damping scheme (D3BJ).^69,70^

All optimizations were performed with tight SCF and geometry convergence criteria. Frequency calculations found all respective optimized geometries to be local minima. Single-point energy calculations used quasi-degenerate perturbation theory (QDPT)^71,72^ with zeroth-order regular approximation (ZORA)^66,73,74^ as implemented in ORCA 5.0.3 in order to gain insights into the luminescence mechanisms in the studied complexes. Single-point calculations using the resultant geometries were performed at the B3LYP/def2-TZVP level of theory using CPCM for toluene. In this case, relativistically corrected triple-ζ basis sets with the zeroth-order regular approximation (ZORA)^73,74^ were used: ZORA-def2-TZVP^66^ with the SARC/J^75^ auxiliary basis for all atoms except Pt for which a segmented all-electron relativistically contracted (SARC) SARC-ZORA-TZVP^75^ basis set was used. Spin–orbit coupling (SOC) calculations were performed as implemented in the ORCA software. SOC matrix elements (SOCMEs) and SOC-corrected excitations (SOC states) were computed using the same settings as above. The RI-SOMF­(1X) setting was used to accelerate the SOC calculations.

#### Calculation of Singlet and Triplet Radiative Rates Using Simulated Parameters

The relationship between the transition oscillator strength and the radiative rate constant is described by the following eq ([Disp-formula eq1])^76^

1 $$k_{r}=\frac{n^{2}v^{2}f}{1.5}$$

where *k*_r_—radiative rate constant of a given transition, s^–1^; *n*—refractive index of the medium; *ν*—energy of the excited state, cm^–1^; *f*—transition oscillator strength. In order to obtain radiative decay rates involving 1, 2, 3 SOC states (*quasi*-degenerate T_1_ sublevels), we consider the thermal equilibrium between them as set out earlier by Mori and others^77^ for calculating phosphorescence rates according to eq ([Disp-formula eq2])

2 $$k_{r}^{1-3}=\frac{k_{r}^{1}+{k_{r}^{2}e}^{-{\Delta E}_{1,2}/k_{b}T}+{k_{r}^{3}e}^{-{\Delta E}_{1,3}/k_{b}T}}{1+e^{-{\Delta E}_{1,2}/k_{b}T}+e^{-{\Delta E}_{1,3}/k_{b}T}}$$

where *k*_r_^1–3^—average T_1_ radiative rate constant, s^–1^; *k*_r_^1^, *k*_r_^2^, *k*_r_^3^—radiative rate constants of the SOC-TDDFT states 1–3, s^–1^; *k*_b_—Boltzmann constant, 8.617 × 10^–5^ eV K^–1^; Δ*E*_1,2_—energy difference between SOC-TDDFT states 1 and 2, eV; Δ*E*_1,3_—energy difference between SOC-TDDFT states 1 and 3, eV; *T*—temperature, *K*. For the purpose of this work, we assume *T* = 295 K unless stated otherwise.

### Antiviral Activity against HCoV-OC43

The antiviral activity of the test compounds against HCoV-OC43 was determined according to a recently reported protocol.^78^ In short, a virus dilution at an MOI (multiplicity of infection) of 0.1 was simultaneously added with the test compounds to an almost confluent layer of RD cells. After 24 h, the incubation was stopped and the viral load was quantified by ELISA using a N-protein primary antibody (N-protein HCoV-OC43, Sino Biological) and an HRP-labeled secondary antibody (Sino Biological). Following the determination of N-positive cells, the plates were washed twice with phosphate-buffered saline, and a crystal violet counter screen was performed to determine cytotoxicity.

### Antiviral Activity against Influenza A Virus

To analyze the effect of the tested compounds on the replication-competent influenza virus A/Puerto Rico/8/34, we infected human lung cancer A549 cells with the virus at an MOI of 0.01, as described previously.^79^ At 48 h postinfection, the virus titers in supernatants were determined with a focus formation assay, as described previously.^79^

### SARS-CoV-2 M^pro^ and Human Serine Protease TMPRSS2 Inhibition

Complexes were tested for their inhibitory activities toward SARS-CoV-2 cysteine-like protease M^pro/^3CL^pro^ and human serine protease TMPRSS2. We used a commercially available SARS-CoV-2 main protease inhibitor screening assay kit (Cayman Chemical, Ann Arbor, MI, USA) and a previously published protocol for TMPRSS2 inhibitors.^80^ Briefly, recombinant enzymes at a final concentration of 0.5 μM were incubated with complexes at different concentrations for 30 min at room temperature. Next, the fluorogenic substrates at a concentration of 20 nM were added and incubated for the next 2 h. The GC376 and camostat at a concentration of 50 μM were used as positive controls for M^pro^ and TMPRSS2, respectively. The fluorescence was measured using Hidex Sense (Hidex, Finland). The assay was performed in triplicate.

## Supplementary Material





## Notes

Dataset xyz coordinates ESI for publication (ZIP)
1H, ^13^C NMR, MS, TGA, elemental analysis, calculations, and crystal data (PDF)

## References

[1] Morgan G. Y., Burstall F. H. (1932). Dehydrogenation of pyridine by anhydrous ferric chloride. J. Chem. Soc..

[2] Taniya O. S., Kopchuk D. S., Khasanov A. F., Kovalev I. S., Santra S., Zyryanow G. V., Majee A., Charushin V. N., Chupakhin O. N. (2021). Synthetic approaches and supramolecular properties
of 2,2′:n’,m”-terpyridine domains (n = 3,4,5,6;
m = 2,3,4) based on the 2,2′-bipyridine core as ligands with *κ*
^2^
*N*-bidentate coordination
mode. Coord. Chem. Rev..

[3] Winter A., Newkome G. R., Schubert U. S. (2024). The chemistry of
the s- and p-block
elements with 2,2′:6′2”-terpyridine ligands. Inorg. Chem. Front..

[4] Eryazici I., Moorefield C. N., Newkome G. R. (2008). Square-planar Pd­(II), Pt­(II), and
Au­(III) terpyridine complexes: their syntheses, physical properties,
supramolecular constructs, and biomedical activities. Chem. Rev..

[5] Schubert, U. S. ; Hofmeier, H. ; Newkome, G. R. Modern Terpyridine Chemistry; Wiley-VCH: Weinheim, Germany, 2006.

[6] Morgan G. T., Burstall F. H. (1934). 323 Researches on residual affinity and co-ordination.
Part XXXV. 2: 2′: 2″-Tripyridylplatinum salts. J. Chem. Soc..

[7] Aguilar
Rico F., Derogar M., Cubo L., Quiroga A. G. (2024). Synthetic routes
and chemical structural analysis for guiding the strategies on new
Pt­(II) metallodrug design. Dalton Trans..

[8] Gao Z., Han Y., Gao Z., Wang F. (2018). Multicomponent assembled systems
based on platinum (II) terpyridine complexes. Acc. Chem. Res..

[9] Mauro M., Aliprandi A., Septiadi D., Kehr N. S., Cola L. D. (2014). When self-assembly
meets biology: luminescent platinum complexes for imaging applications. Chem. Soc. Rev..

[10] Lee L. C.-C., Lo K. K.-W. (2024). Shining new light
on biological systems: luminescent
transition metal complexes for bioimaging and biosensing applications. Chem. Rev..

[11] dos
Santos P. L., Stachelek P., Takeda Y., Pander P. (2024). Recent advances
in highly-efficient near infrared OLED emitters. Mater. Chem. Front..

[12] McMillin D. R., Moore J. J. (2002). Luminescence that lasts from Pt­(trpy)­Cl+ derivatives
(trpy = 2,2′;6′,2″-terpyridine). Coord. Chem. Rev..

[13] Aldridge T. K., Stacy E. M., McMillin D. R. (1994). Studies of the Room-Temperature
Absorption
and Emission Spectra of [Pt­(trpy)­X]+ Systems. Inorg. Chem..

[14] Crites D. K., Cunningham C. T., McMillin D. R. (1998). Remarkable substituent effects on
the photophysics of Pt­(4′-X-trpy)­Cl^+^ systems (trpy
= 2,2′; 6′,2″-terpyridine). Inorg. Chim. Acta.

[15] Michalec J. F., Bejune S. A., McMillin D. R. (2000). Multiple ligand-based
emissions from
a platinum­(II) terpyridine complex attached to pyrene. Inorg. Chem..

[16] Hight L. M., McGuire M. C., Zhang Y., Bork M. A., Fanwick P. E., Wasserman A., McMillin D. R. (2013). *π* Donation
and its effects on the excited-state lifetimes of luminescent platinum­(II)
terpyridine complexes in solution. Inorg. Chem..

[17] Tarran W. A., Freeman G. R., Murphy L., Benham A. M., Kataky R., Williams J. A. G. (2014). Platinum­(II)
complexes of N^Λ^C^Λ^N-coordinating
1,3-Bis­(2-pyridyl)­benzene ligands: thiolate
coligands lead to strong red luminescence from charge-transfer states. Inorg. Chem..

[18] Salthouse R. J., Sil A., Gildea L. F., Yufit D. S., Williams J. A. G. (2023). Platinum­(II)
complexes of nonsymmetrical *NCN*-coordinating ligands:
unimolecular and excimeric luminescence properties and comparison
with symmetrical analogues. Inorg. Chem..

[19] Salthouse R. J., Sil A., Pander P., Dias F. B., Williams J. A. G. (2025). Molecular rectangles
featuring two parallel *NCN*-coordinated platinum units:
enhancing near-infrared emission through excimer formation. Chem. - Eur. J..

[20] Paderina A., Slavova S., Tupikina E., Snetkov D., Grachova E. (2024). Aggregation
game: changing solid-state emission using different counterions in
monoalkynylphosphonium Pt­(II) complexes. Inorg.
Chem..

[21] Hattori S., Kawajiri S., Sekine A., Sumi K., Narumiya H., Sato K., Shinozaki K. (2023). Kinetic formation of Pt-Pt dimers
of cationic and neutral platinum­(II) complexes under rapid freeze
conditions in solution. Inorg. Chem..

[22] Salthouse R. J., Pander P., Yufit D. S., Dias F. B., Williams J. A. G. (2022). Near-infrared
electroluminescence beyond 940 nm in Pt­(N^Λ^C^Λ^N)­X complexes: influencing aggregation with the ancillary ligand
X. Chem. Sci..

[23] Pander P., Zaytsev A. V., Sil A., Williams J. A. G., Kozhevnikov V. N., Dias F. B. (2022). Enhancement of thermally activated delayed fluorescence
properties by substitution of ancillary halogen in a multiple resonance-like
diplatinum­(II) complex. J. Mat. Chem. C.

[24] Wadas T. J., Wang Q.-M., Kim Y.-j., Flaschenreim C., Blanton T. N., Eisenberg R. (2004). Vapochromism
and its structural basis
in a luminescent Pt­(II) terpyridine-nicotinamide complex. J. Am. Chem. Soc..

[25] Tashiro K., Ohtsu H., Kawano M., Kojima T., Kato T. (2018). Platinum­(II)
terpyridine complex that switches its photochemical reactivity to
its chromic behavior in the crystalline state. Inorg. Chem..

[26] Bryant M. J., Skelton J. M., Hatcher L. E., Stubbs C., Madrid E., Pallipurath A. R., Thomas L. H., Woodall C. H., Christensen J., Fuertes S., Robinson T. P., Beavers C. M., Teat S. J., Warren M. R., Pradaux-Caggiano F., Walsh A., Marken F., Carbery D. R., Parker S. C., McKeown N. B., Malpass-Evans R., Carta M., Raithby P. R. (2017). A rapidly-reversible absorptive and
emissive vapochromic Pt­(II) pincer-based chemical sensor. Nat. Commun..

[27] Nishiuchi Y., Takayama A., Suzuki T., Shinozaki K. (2011). A polymorphic
platinum­(II) complex: yellow, red, and green polymorphs and X-ray
crystallography of [Pt­(fdpb)­Cl] [Hfdpb = 1,3-bis­(5-trifluoromethyl-2-pyridyl)­benzene]. Eur. J. Inorg. Chem..

[28] Muggia F. M., Bonetti A., Hoeschele J. D., Rozencweig M., Howell S. B. (2015). Platinum antitumor complexes: 50 years since Barnett
Rosenberg’s discovery. J. Clinic. Oncol..

[29] Lowe G., Droz A. S., Vilaivan T., Weaver G. W., Tweedale L., Pratt J. M., Rock P., Yardley V., Croft S. L. (1999). Cytotoxicity
of (2,2′:6′,2”-terpyridine)­platinum­(II) complexes
to *Leishmania donovani, Trypanosoma cruzi, and Trypanosoma
brucei*. J. Med. Chem..

[30] Bonse S., Richards J. M., Ross S. A., Lowe G., Krauth-Siegel R. L. (2000). (2,2′:6′,2”-terpyridine)­platinum­(II)
complexes are irreversible inhibitors of *Trypanosoma cruzi* trypanothione reductase but not of human glutathione reductase. J. Med. Chem..

[31] Becker K., Herold-Mende C., Park J. J., Lowe G., Schirmer R. H. (2001). Human Thioredoxin
Reductase Is Efficiently Inhibited by (2,2′:6′,2‘
‘-Terpyridine)­platinum­(II) Complexes. Possible Implications
for a Novel Antitumor Strategy. J. Med. Chem..

[32] McFadyen W. D., Wakelin L. P., Roos I. A., Leopold V. A. (1985). Activity of platinum­(II)
intercalating agents against murine leukemia L1210. J. Med. Chem..

[33] Tong K.-C., Wan P.-K., Lok C.-N., Che C.-M. (2021). Dynamic supramolecular
self-assembly of platinum­(II) complexes perturbs an autophagy–lysosomal
system and triggers cancer cell death. Chem.
Sci..

[34] Lo Y.-C., Su W.-C., Ko T.-P., Wang N.-C., Wang A. H.-J. (2011). Terpyridine
platinum­(II) complexes inhibit cysteine proteases by binding to active-site
cysteine. J. Biomol. Struct. Dyn..

[35] Chung C. Y.-S., Li S. P.-Y., Lo K. K.-W., Yam V. W.-W. (2016). Synthesis and
electrochemical, photophysical, and self-assembly studies on water-soluble
pH-responsive alkynylplatinum­(II) terpyridine complexes. Inorg. Chem..

[36] De
Castro F., De Luca E., Benedetti M., Fanizzi F. P. (2022). Platinum compounds as potential antiviral agents. Coord. Chem. Rev..

[37] Bailly B., Gorle A. K., Dirr L., Malde A. K., Farrell N. P., Berners-Price S. J., von Itzstein M. (2021). Platinum complexes
act as shielding
agents against virus infection. Chem. Commun..

[38] Gil-Moles M., Türck S., Basu U., Pettenuzzo A., Bhattacharya S., Rajan A., Ma X., Büssing R., Wölker J., Burmeister H., Hoffmeister H., Schneeberg P., Prause A., Lippmann P., Kusi-Nimarko J., Hassell-Hart S., McGown A., Guest D., Lin Y., Notaro A., Vinck R., Karges J., Cariou K., Peng K., Qin X., Wang X., Skiba J., Szczupak Ł., Kowalski K., Schatzschneider U., Hemmert C., Gornitzka H., Milaeva E. R., Nazarov A. A., Gasser G., Spencer J., Ronconi L., Kortz U., Cinatl J., Bojkova D., Ott I. (2021). Metallodrug profiling
against SARS-CoV-2 target proteins identifies highly potent inhibitors
of the S/ACE2 interactions and the papain-like protease PL^pro^. Chem.–Eur. J..

[39] De
Clercq E., Li G. (2016). Approved antiviral drugs over the
past 50 years. Clin. Microbiol. Rev..

[40] Janzen L., Miller R. G., Metzler-Nolte N. (2024). Synthesis,
characterisation and antimicrobial
activity of supramolecular cobalt-peptide conjugates. Dalton Trans..

[41] Pander P., Sil A., Salthouse R. J., Harris C. W., Walden M. T., Yufit D. S., Williams J. A. G., Dias F. B. (2022). Excimer
or aggregate? Near infrared electro- and photoluminescence
from multimolecular excited states of N^C^N-coordinated platinum­(II)
complexes. J. Mater. Chem. C Mater..

[42] Williams J. A. G. (2009). The
coordination chemistry of dipyridylbenzene: N-deficient terpyridine
or panacea for brightly luminescent metal complexes?. Chem. Soc. Rev..

[43] Williams J. A. G., Beeby A., Davies E. S., Weinstein J. A., Wilson C. (2003). An Alternative Route to Highly Luminescent Platinum­(II)
Complexes: Cyclometalation with N/\C/\N-Coordinating Dipyridylbenzene
Ligands. Inorg. Chem..

[44] Wei Y.-C., Wang S. F., Hu Y., Liao L.-S., Chen D.-G., Chang K.-H., Wang C.-W., Liu S.-H., Chan W.-H., Liao J.-L., Hung W.-Y., Wang T.-H., Chen P.-T., Hsu H.-F., Chi Y., Chou P.-T. (2020). Overcoming the energy
gap law in near-infrared OLEDs by exciton–vibration decoupling. Nat Photonics.

[45] Zieger S. E., Steinegger A., Klimant I., Borisov S. M. (2020). TADF-Emitting
Zn­(II)-Benzoporphyrin:
An Indicator for Simultaneous Sensing of Oxygen and Temperature. ACS Sens..

[46] Cocchi M., Kalinowski J., Virgili D., Williams J. A. G. (2008). Excimer-based
red/near-infrared organic light-emitting diodes with very high quantum
efficiency. Appl. Phys. Lett..

[47] Kim D., Brédas J. L. (2009). Triplet excimer formation in platinum-based phosphors:
A theoretical study of the roles of Pt-Pt bimetallic interactions
and interligand π-π interactions. J. Am. Chem. Soc..

[48] Neese F. (2012). The ORCA program
system. Wiley Interdiscip. Rev.:Comput. Mol.
Sci..

[49] Neese F. (2022). Software update:
The ORCA program system—Version. Wiley
Interdiscip. Rev.:Comput. Mol. Sci..

[50] Neese F. (2018). Software update:
the ORCA program system, version 4.0. Wiley
Interdiscip. Rev.:Comput. Mol. Sci..

[51] Teunissen J. L., De Proft F., De Vleeschouwer F. (2017). Tuning the HOMO–LUMO Energy
Gap of Small Diamondoids Using Inverse Molecular Design. J. Chem. Theory. Comput..

[52] Englman R., Jortner J. (1970). The energy gap law
for radiationless transitions in
large molecules. Mol. Phys..

[53] V’kovski P., Kratzel A., Steiner S., Stadler H., Thiel V. (2021). Coronavirus
biology and replication: implications for SARS-CoV-2. Nat. Rev..

[54] CrysAlis CCD and CrysAlis RED, Oxford Diffraction;; Oxford Diffraction Ltd.: Yarnton, 2008.

[55] Sheldrick G. M. (2015). SHELXT
- Integrated space-group and crystal-structure determination. Acta Crystallogr., Sect. A:Found. Adv..

[56] Sheldrick G. M. (2015). Crystal
structure refinement with SHELXL. Acta Crystallogr.,
Sect. C:Struct. Chem..

[57] Spek A. L. (2009). Structure
validation in chemical crystallography. Acta
Crystallogr.,Sect. D:Biol. Cryst..

[58] Dolomanov O. V., Bourhis L. J., Gildea R. J., Howard J. A. K., Puschmann H. (2009). OLEX2: a complete
structure solution, refinement and analysis program. J. Appl. Crystallogr..

[59] Macrae C. F., Bruno I. J., Chisholm J. A., Edgington P. R., McCabe P., Pidcock E., Rodriguez-Monge L., Taylor R., van de Streek J., Wood P. A. (2008). Mercury CSD 2.0–
new features for the visualization and investigation of crystal structures. J. Appl. Crystallogr..

[60] Pander P., Data P., Dias F. B. (2018). Time-resolved Photophysical
Characterization
of Triplet-harvesting Organic Compounds at an Oxygen-free Environment
Using an iCCD Camera. J. Vis. Exp..

[61] Lehtola S., Steigemann C., Oliveira M. J. T., Marques M. A. L. (2018). Recent developments
in libxc—A comprehensive library of functionals for density
functional theory. SoftwareX.

[62] Allouche A.-R. (2011). Gabedit-A
graphical user interface for computational chemistry softwares. J. Comput. Chem..

[63] Avogadro: an open-source molecular builder and visualization tool. Version 1.2.0. https://avogadro.cc.

[64] Hanwell M. D., Curtis D. E., Lonie D. C., Vandermeersch T., Zurek E., Hutchison G. R. (2012). Avogadro: an advanced semantic chemical
editor, visualization, and analysis platform. J Cheminform..

[65] Becke A. D. (1988). Density-functional
exchange-energy approximation with correct asymptotic behavior. Phys. Rev. A.

[66] Weigend F., Ahlrichs R. (2005). Balanced basis sets of split valence,
triple zeta valence
and quadruple zeta valence quality for H to Rn: Design and assessment
of accuracy. Phys. Chem. Chem. Phys..

[67] Hellweg A., Hättig C., Höfener S., W Klopper W. (2007). Optimized
accurate auxiliary basis sets for RI-MP2 and RI-CC2 calculations for
the atoms Rb to Rn. Theor. Chem. Acc..

[68] Weigend F. (2006). Accurate Coulomb-fitting
basis sets for H to Rn. Phys. Chem. Chem. Phys..

[69] Grimme S., Ehrlich S., Goerigk L. (2011). Effect of
the damping function in
dispersion corrected density functional theory. J. Comput. Chem..

[70] Grimme S., Antony J., Ehrlich S., Krieg H. (2010). A consistent and accurate
ab initio parametrization of density functional dispersion correction
(DFT-D) for the 94 elements H-Pu. J. Chem. Phys..

[71] Roemelt M., Maganas D., DeBeer S., Neese F. (2013). A combined DFT and
restricted open-shell configuration interaction method including spin-orbit
coupling: Application to transition metal L-edge X-ray absorption
spectroscopy. J. Chem. Phys..

[72] de
Souza B., Farias G., Neese F., Izsák R. (2019). Predicting
Phosphorescence Rates of Light Organic Molecules Using Time-Dependent
Density Functional Theory and the Path Integral Approach to Dynamics. J. Chem. Theory. Comput..

[73] Lenthe E. v., Baerends E. J., Snijders J. G. (1993). Relativistic
regular two-component
Hamiltonians. J. Chem. Phys..

[74] van
Lenthe E., Baerends E. J., Snijders J. G. (1994). Relativistic total
energy using regular approximations. J. Chem.
Phys..

[75] Pantazis D.
A., Chen X. Y., Landis C. R., Neese F. (2008). All-electron scalar
relativistic basis sets for third-row transition metal atoms. J. Chem. Theory. Comput..

[76] Nozaki K. (2006). Theoretical
Studies on Photophysical Properties and Mechanism of Phosphorescence
in [*fac*-Ir­(2-phenylpyridine)­3]. J. Chin. Chem. Soc..

[77] Mori K., Goumans T. P. M., van Lenthe E., Wang F. (2014). Predicting phosphorescent
lifetimes and zero-field splitting of organometallic complexes with
time-dependent density functional theory including spin–orbit
coupling. Phys. Chem. Chem. Phys..

[78] Gil-Moles M., J Füllborn-Ott, Brescia F., Ronconi L., Ott I. (2025). Potent inhibition of
SARS-CoV-2 proteases PL^pro^ and 3CL^pro^ by selected
gold­(III)-dithiocarbamato complexes showing strong and selective antiviral
activity against HCoV-OC43. J. Inorg. Biochem..

[79] Zmora P., Molau-Blazejewska P., Bertram S., Walendy-Gnirß K., Nehlmeier I., Hartleib A., Moldenhauer A.-S., Konzok S., Dehmel S., Sewald K., Brinkmann C., Curths C., Knauf S., Gruber J., Mätz-Rensing K., Dahlmann F., Braun A., Pöhlmann S. (2017). Non-human
primate orthologues of TMPRSS2 cleave and activate the influenza virus
hemagglutinin. PLoS One.

[80] Mahoney M., Damalanka V. C., Tartell M. A., Chung Dh., Lourenço A. L., Pwee D., Bridwell A. E. M., Hoffmann M., Voss J., Karmakar P., Azouz N. P., Klingler A. M., Rothlauf P. W., Thompson C. E., Lee M., Klampfer L., Stallings C. L., Rothenberg M. E., Pöhlmann S., Whelan S. P. J., O’Donoghue A. J., Craik C. S., Janetka J. W. (2021). A novel class of TMPRSS2 inhibitors
potently block SARS-CoV-2 and MERS-CoV viral entry and protect human
epithelial lung cells. Proc. Nat. Acad. Sci.
U. S. A..

