# Supplementary material for: Bulkiness Makes the Difference: Modulating Solid-State Emission of Anticoronavirus Active Pt(tpy)C(O)NHCH2R Complexes Featuring Adamantyl vs Propargyl R Groups
Source: Inorg Chem. 2026 Jul 17;65(30):17630–44. doi: 10.1021/acs.inorgchem.6c02335 (PMC13439668; doi:10.1021/acs.inorgchem.6c02335)
Supplement: Supplementary file 1 [file ic6c02335_si_002.pdf]

Supporting Information

Bulkiness makes the difference: modulating solid-state emission of anti-coronavirus active $Pt(tpy)C(O)NHCH_2R$ complexes featuring adamantyl *vs* propargyl R groups

Mateusz Klarek,[a] Daria Wysocka,[a] Judith Füllborn-Ott,[b] Damian Trzybiński,[c] Krzysztof Woźniak,[c] Paulina H. Marek-Urban,[d,e] Piotr Pander,[d,f,g] Ingo Ott,[b] Rafał Nowak,[h] Paweł Zmora,[h] and Konrad Kowalski*[a]

[a]*University of Łódź, Faculty of Chemistry, Department of Organic Chemistry, Tamka 12, 91-403 Łódź, Poland;*

[b]*Institute of Medicinal and Pharmaceutical Chemistry, Technische Universität Braunschweig, Beethovenstr. 55, 38-106 Braunschweig, Germany;*

[c]*Faculty of Chemistry, Biological and Chemical Research Centre, University of Warsaw, Żwirki i Wigury 101, 02-089 Warsaw, Poland;*

[d]*Faculty of Chemistry, Silesian University of Technology, Strzody 9, 44-100 Gliwice, Poland;*

[e]*Faculty of Chemistry, Warsaw University of Technology, Noakowskiego 3, 00-664 Warsaw, Poland;*

[f]*Centre for Organic and nanohybrid Electronics, Silesian University of Technology, Konarskiego 22B, 44-100 Gliwice, Poland;*

[g]*Department of Physics, Durham University, South Road, DH1 3LE Durham, UK;*

[h]*Department of Molecular Virology, Institute of Bioorganic Chemistry Polish Academy of Sciences, Noskowskiego 12/14, 61-704 Poznan, Poland.*

Correspondence should be addressed to: konrad.kowalski@chemia.uni.lodz.pl (Konrad Kowalski)

Table of Contents

Fig. S1-S4 ^{1}H-NMR spectrum of **L1** in DMSO-d6 (600 MHz) **S3-S6**

Fig. S5-S7 ^{1}H-NMR spectrum of **L2** in DMSO-d6 (600 MHz) **S7-S9**

Fig. S8-S10 ^{1}H-NMR spectrum of **[L1PtCl]PF$_6$** in DMSO-d6 (600 MHz) **S10-S12**

Fig. S11-S13 ^{1}H-NMR spectrum of **[L2PtCl]PF$_6$** in DMSO-d6 (600 MHz) **S13-S15**

Fig. S14-S16 ^{13}C -NMR spectrum of **L1** in DMSO-d6 (600 MHz) **S16-S18**

Fig. S17-S19 ^{13}C-NMR spectrum of **L2** in DMSO-d6 (600 MHz) **S19-S21**

Fig. S20-S22 ^{13}C-NMR spectrum of **[L1PtCl]PF$_6$** in DMSO-d6 (600 MHz**)** **S22-S24**

Fig. S23-S25 ^{13}C-NMR spectrum of **[L2PtCl]PF$_6$** in DMSO-d6 (600 MHz) **S25-S27**

Fig. S26 HRMS of **L1** **S28**

Fig. S27 HRMS of **L2** **S29**

Fig. S28 HRMS of **[L1PtCl]PF$_6$** **S30**

Fig. S29 HRMS of **[L2PtCl]PF$_6$** **S31**

Fig. S30 HRMS of **[L2PtCl]PF$_6$·H$_2$O** **S32**

Fig. S31 TGA of **[L2PtCl]PF$_6$·H$_2$O** **S33**

Fig. S32 TGA of **[L2PtCl]PF$_6$·H$_2$O** **S34**

Fig. S33 Elemental analysis results for **[L2PtCl]PF$_6$·H$_2$O** **S35**

Fig. S34 Supramolecular architecture in the crystal of **L1** **S36**

Fig. S35 Supramolecular architecture in the crystal of **L2** **S37**

Fig. S36 Photoluminescence before and after heating a sample of **[L2PtCl]PF$_6$·H$_2$O** **S38**

Fig. S37 HOMO of **[L2PtCl]PF$_6$** at the T_1 geometry with an iso-value of 0.01 **S39**

Table S1 Crystal data and structure refinement for **L1** and **L2** **S40**

Table S2 Bond lengths for **L1** [Å] **S41**

Table S3 Values of valence angles for **L1** [°] **S42**

Table S4 Values of torsion angles for **L1** [°] **S43**

Table S5 Bond lengths for **L2** [Å] **S44**

Table S6 Values of valence angles for **L2** [°] **S45**

Table S7 Values of torsion angles for **L2** [°] **S46**

Table S8 Intermolecular hydrogen-bond geometry in the crystal of **L1** **S47**

Table S9. The geometry of π–π interactions in the crystal of **L1** **S47**

Table S10. Intermolecular hydrogen-bond geometry in the crystal of **L2** **S47**

Table S11. The geometry of the C−H···π contacts in the crystal of **L2** **S47**

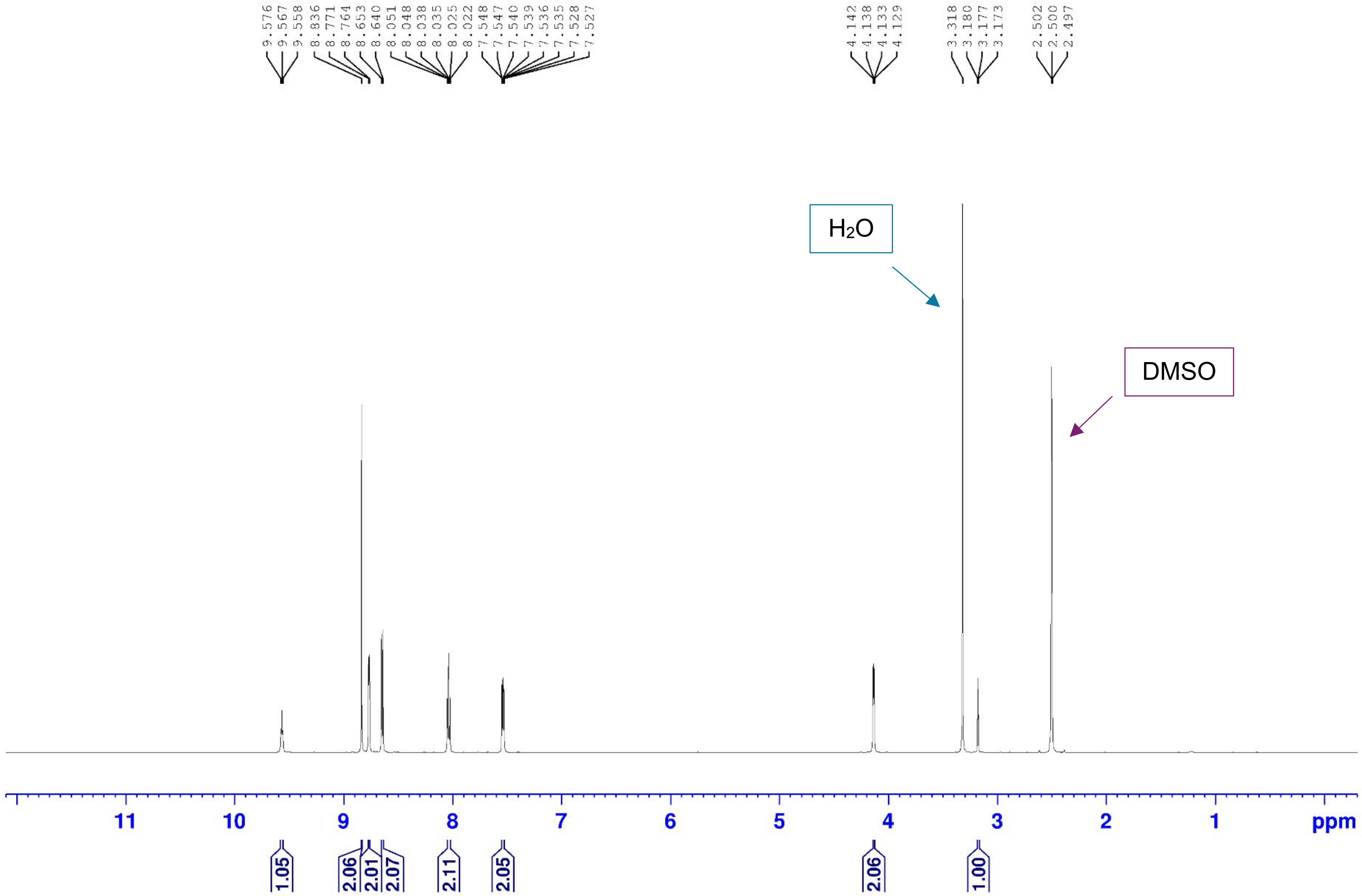

Fig. S1 ^{1}H-NMR spectrum of **L1** in DMSO-d_6 (600 MHz)

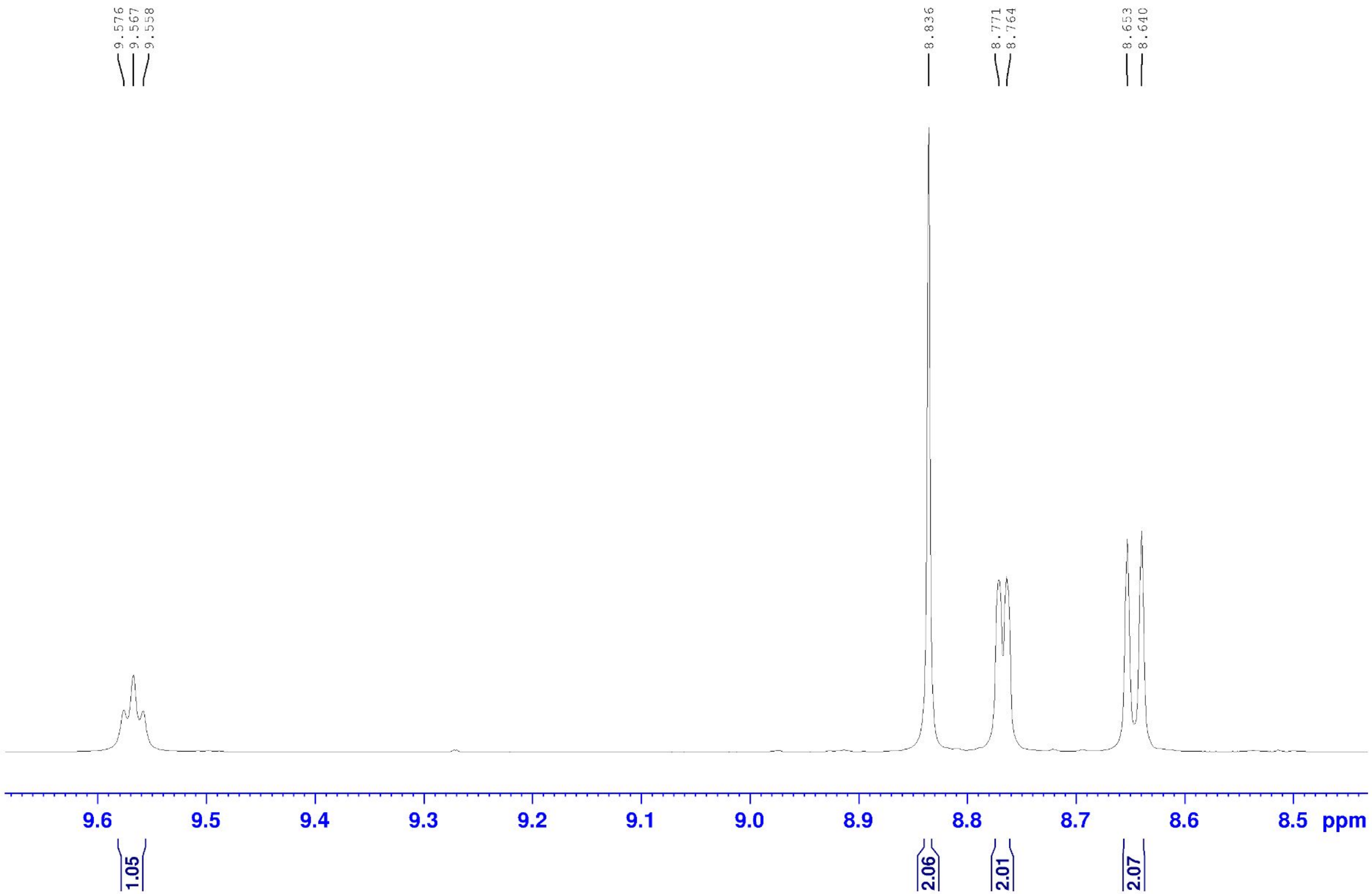

Fig. S2 ^{1}H-NMR spectrum of **L1** in DMSO-d_6 (600 MHz)

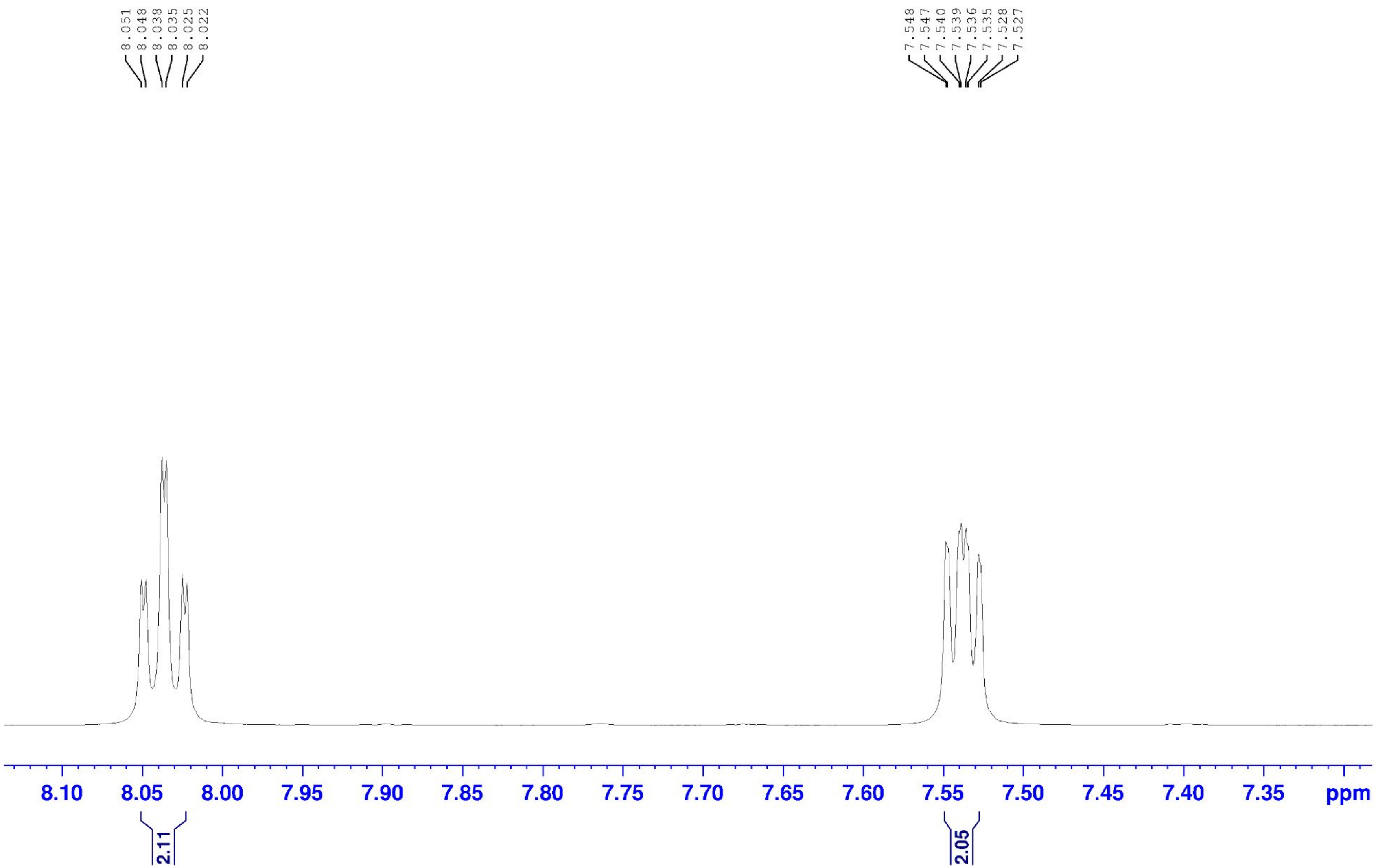

Fig. S3 ^{1}H-NMR spectrum of **L1** in DMSO-d_6 (600 MHz)

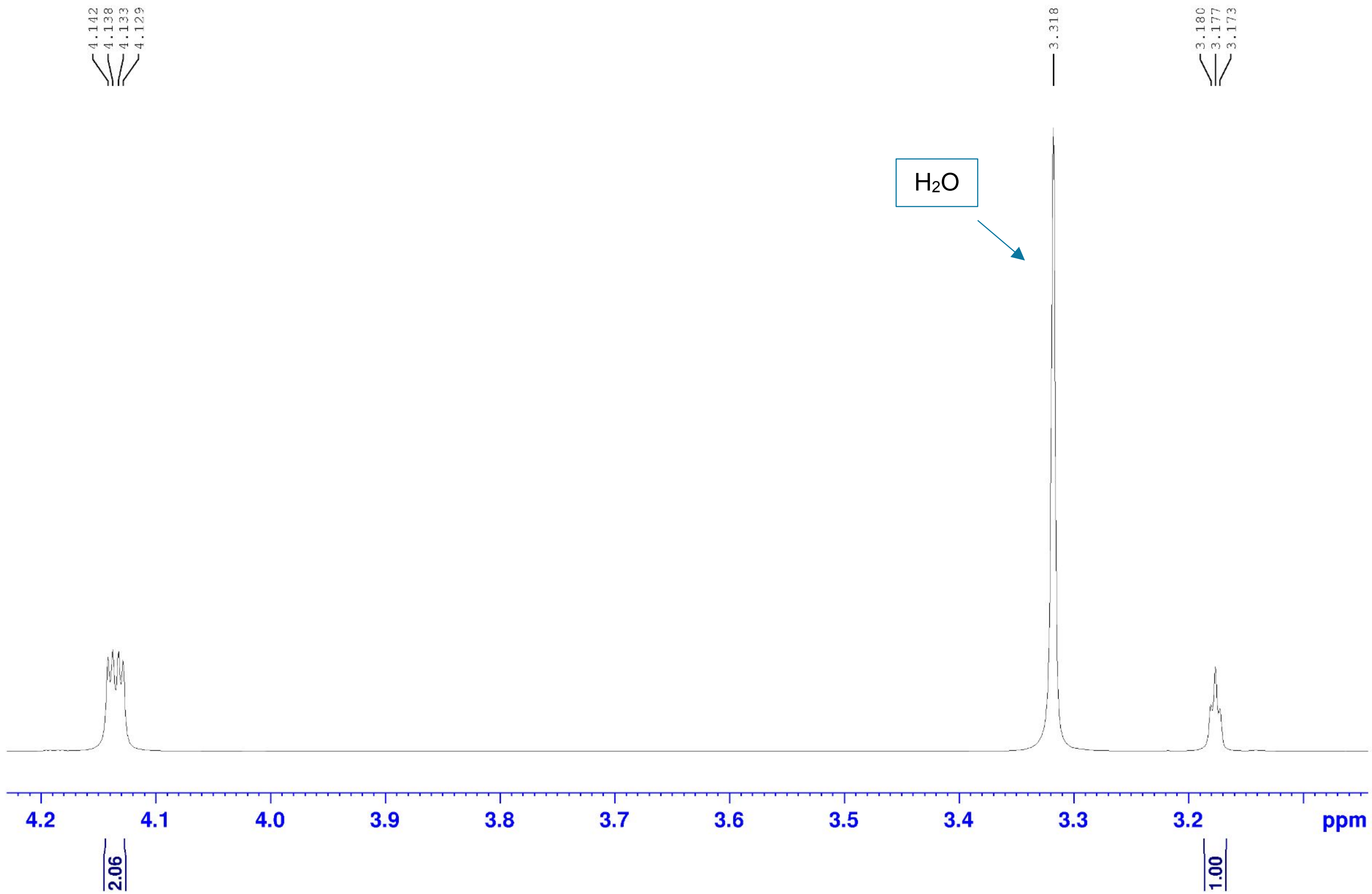

Fig. S4 ^{1}H-NMR spectrum of **L1** in DMSO-d_6 (600 MHz)

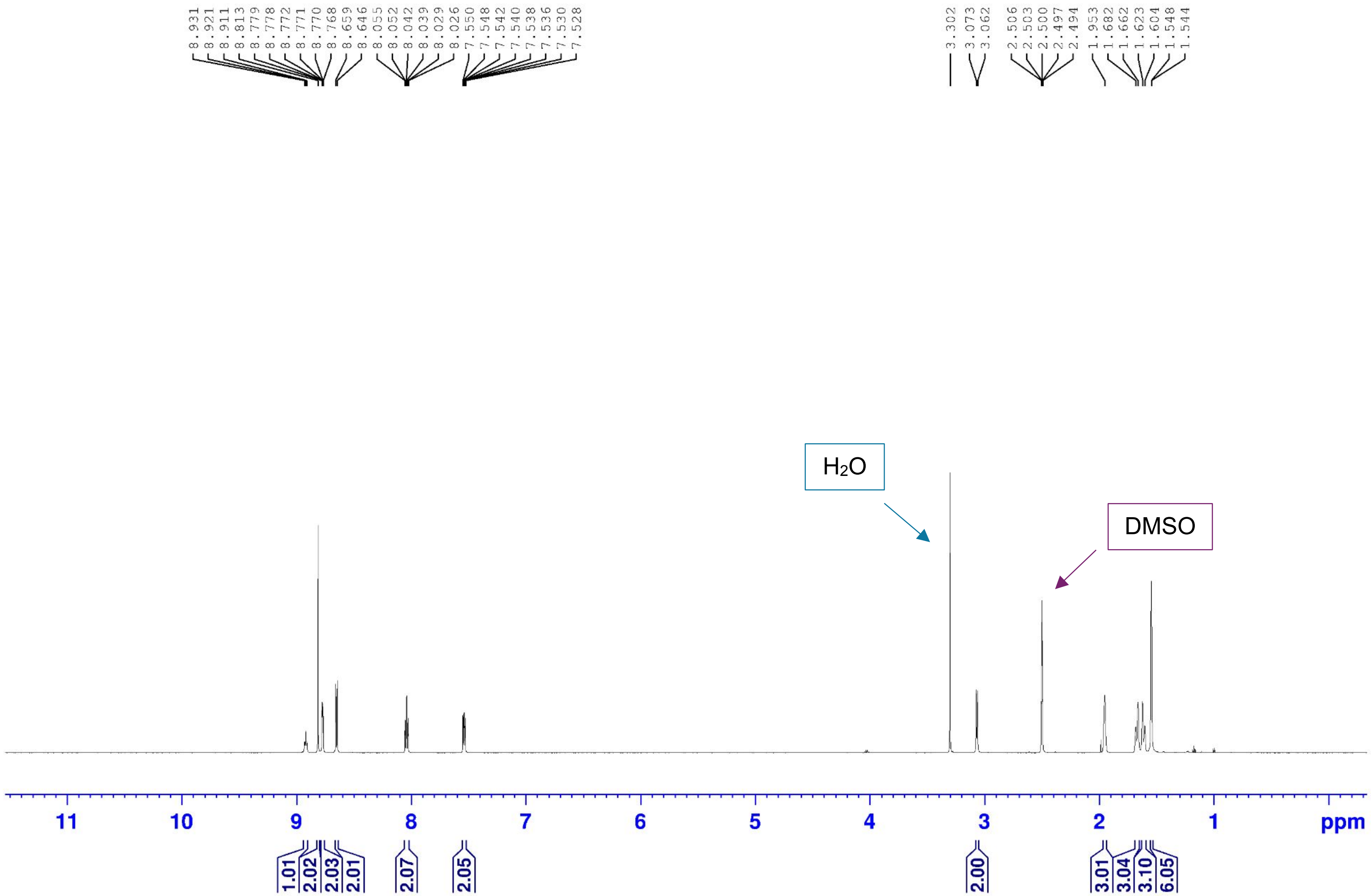

Fig. S5 ^{1}H-NMR spectrum of **L2** in DMSO-d_6 (600 MHz)

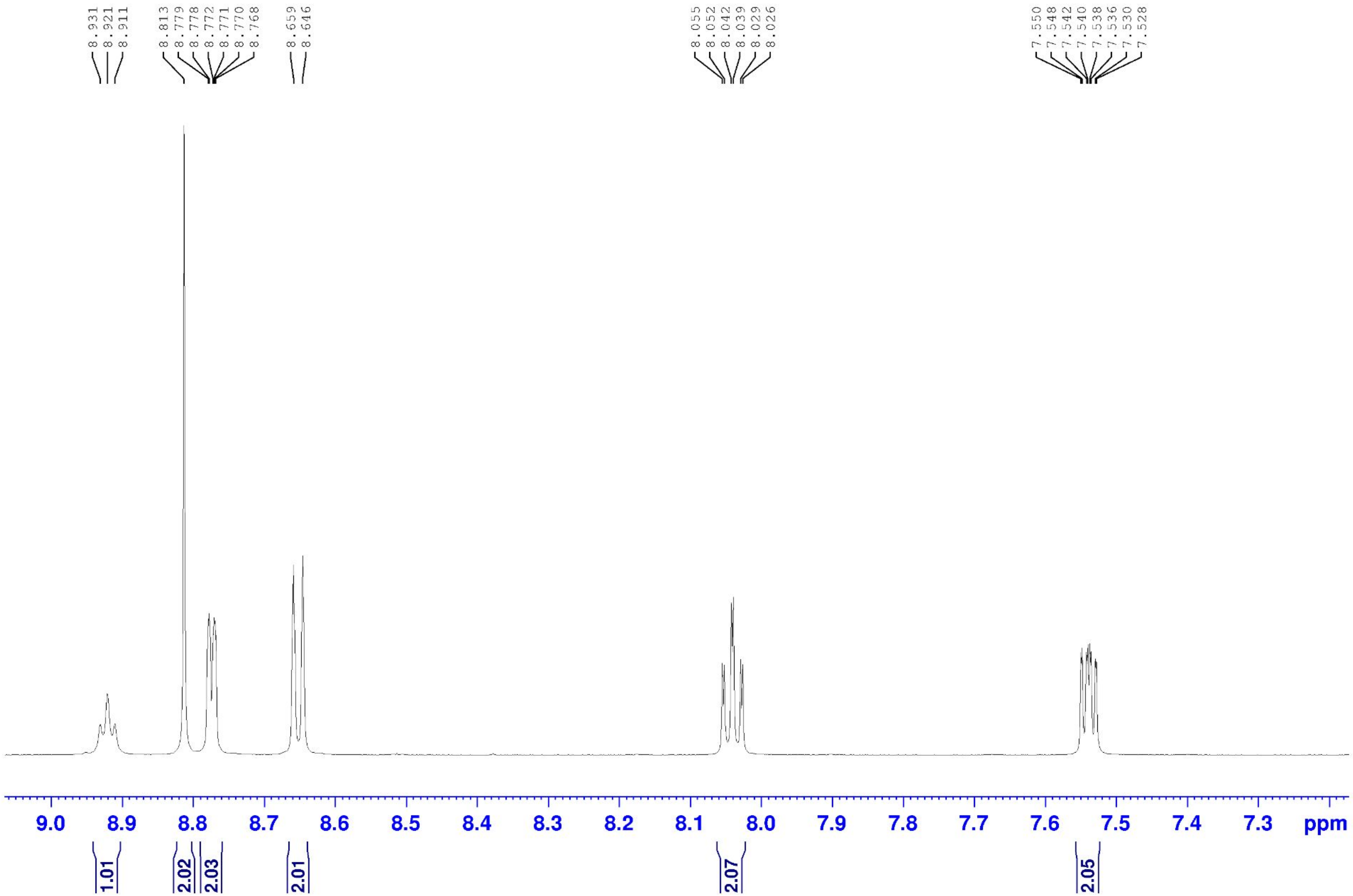

Fig. S6 ^{1}H-NMR spectrum of **L2** in DMSO-d_6 (600 MHz)

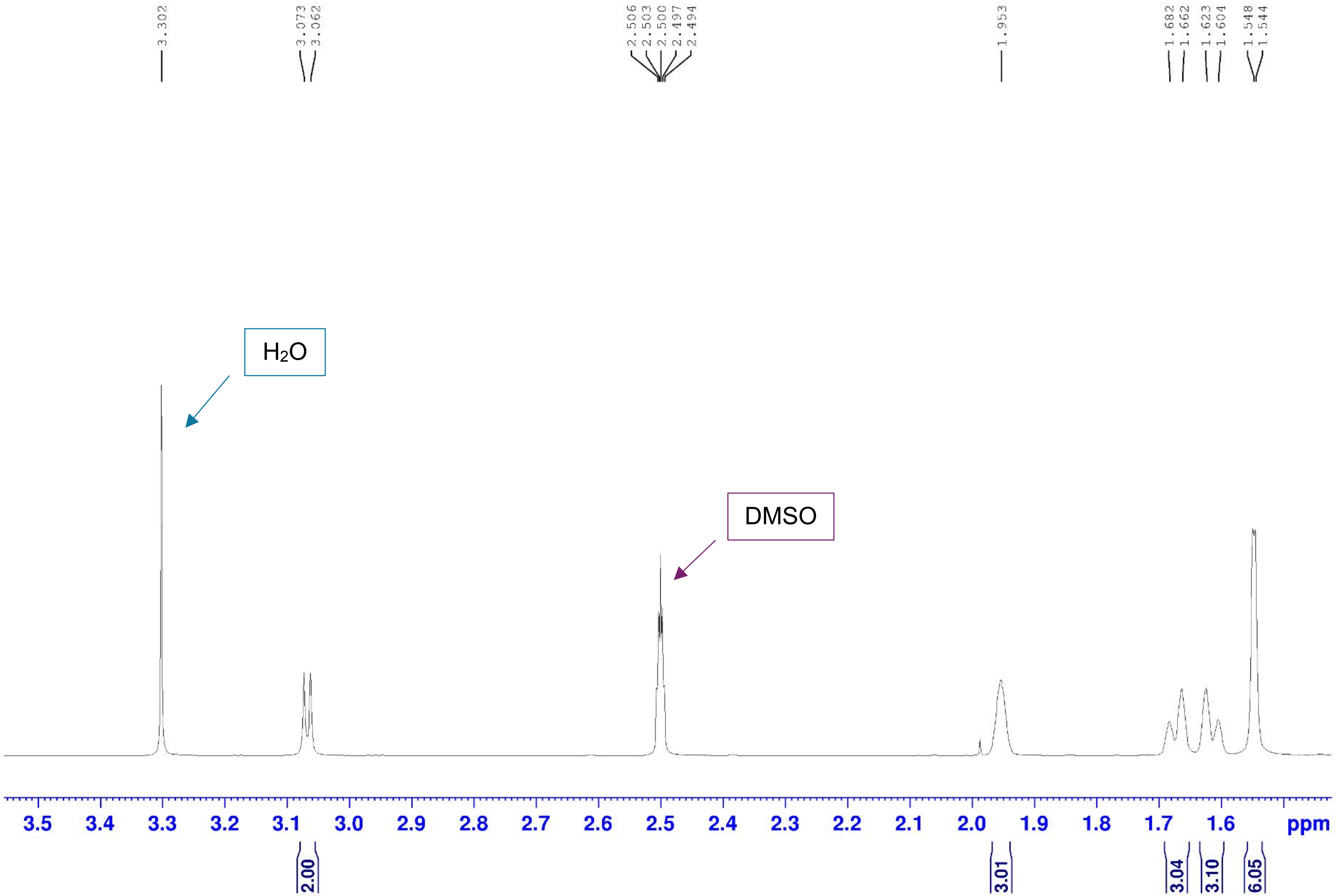

Fig. S7 ^{1}H-NMR spectrum of **L2** in DMSO-d_6 (600 MHz)

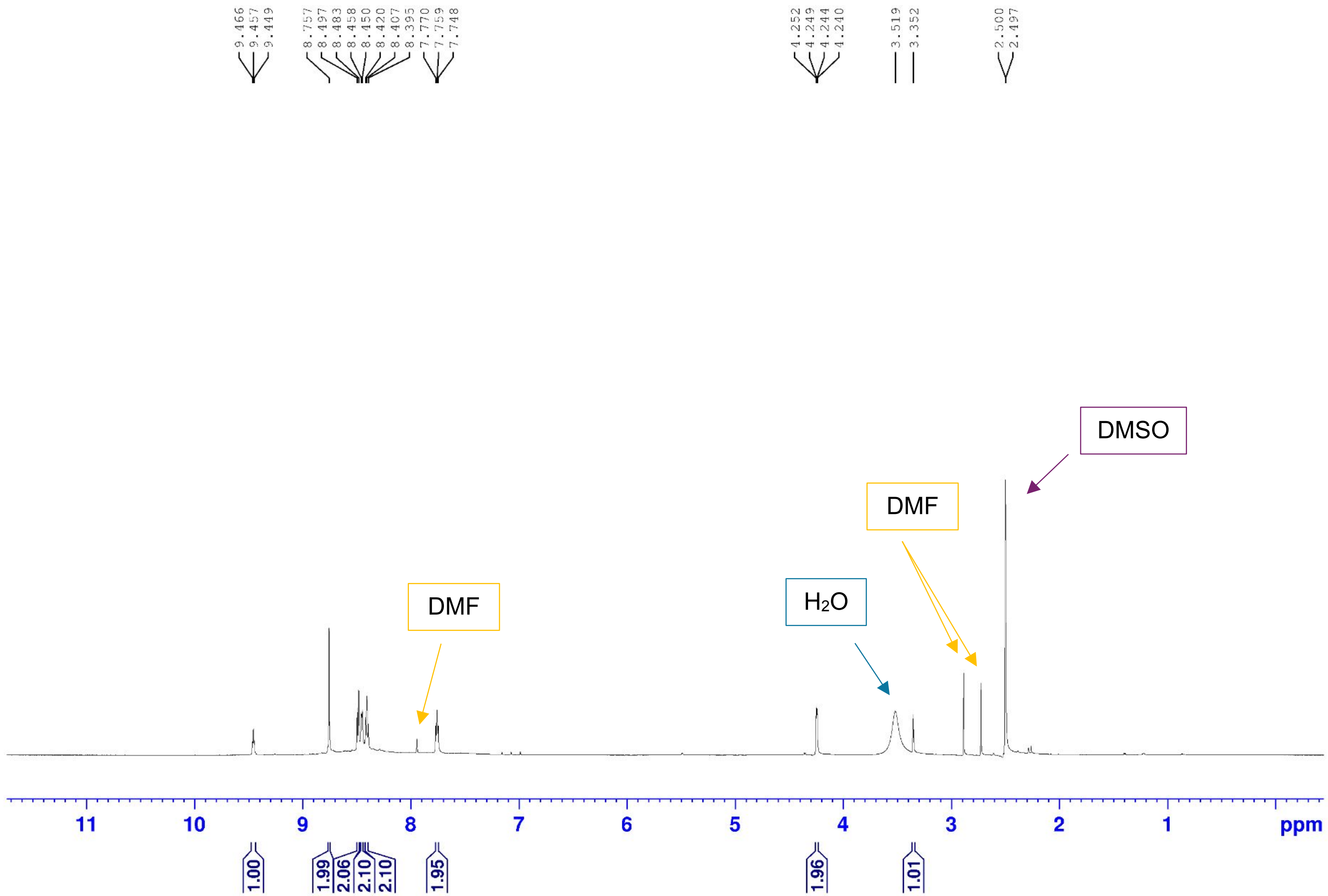

Fig. S8 ^{1}H-NMR spectrum of **[L1PtCl]PF$_6$** in DMSO-d$_6$ (600 MHz)

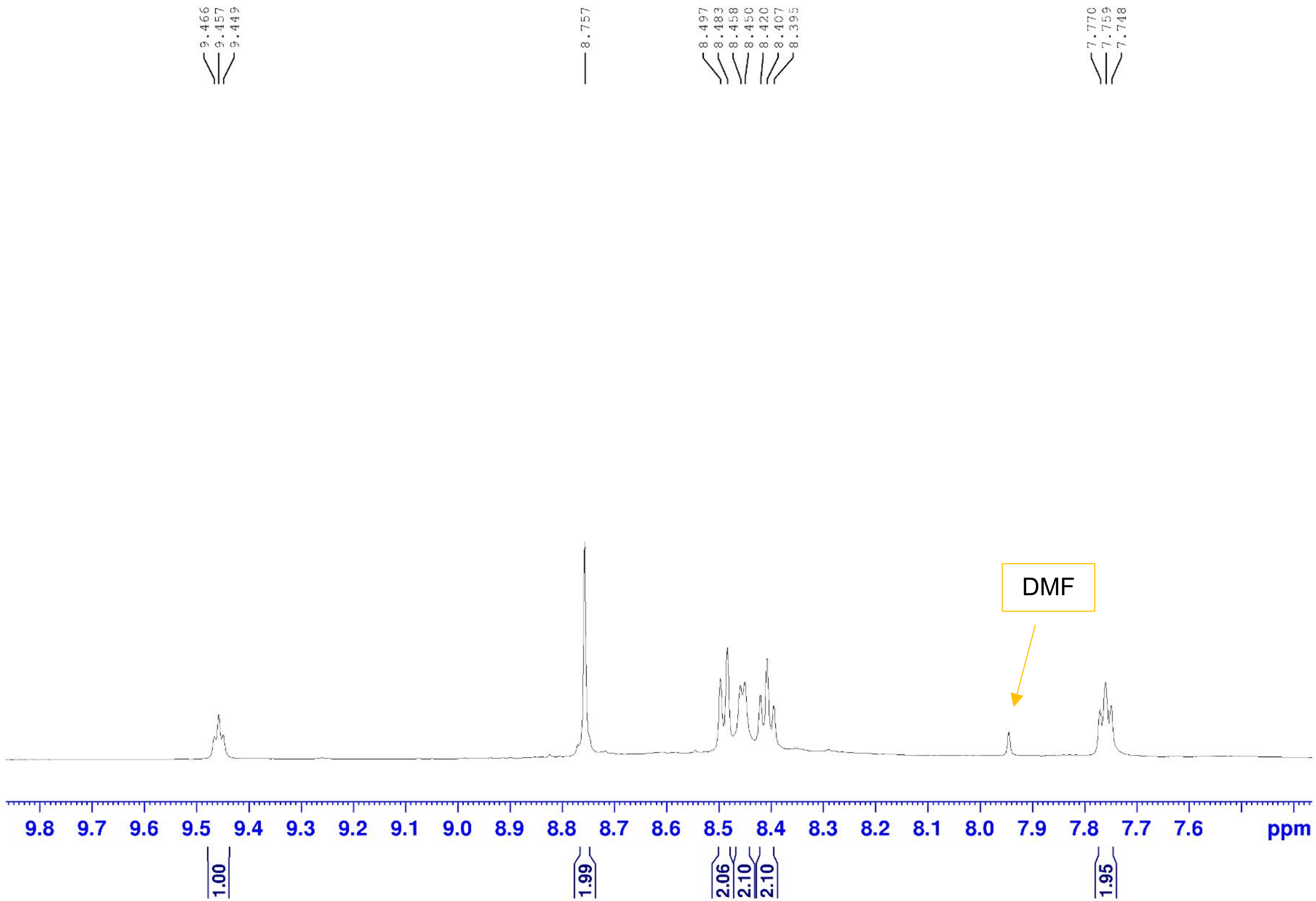

Fig. S9 ^{1}H-NMR spectrum of **[L1PtCl]PF$_6$** in DMSO-d$_6$ (600 MHz)

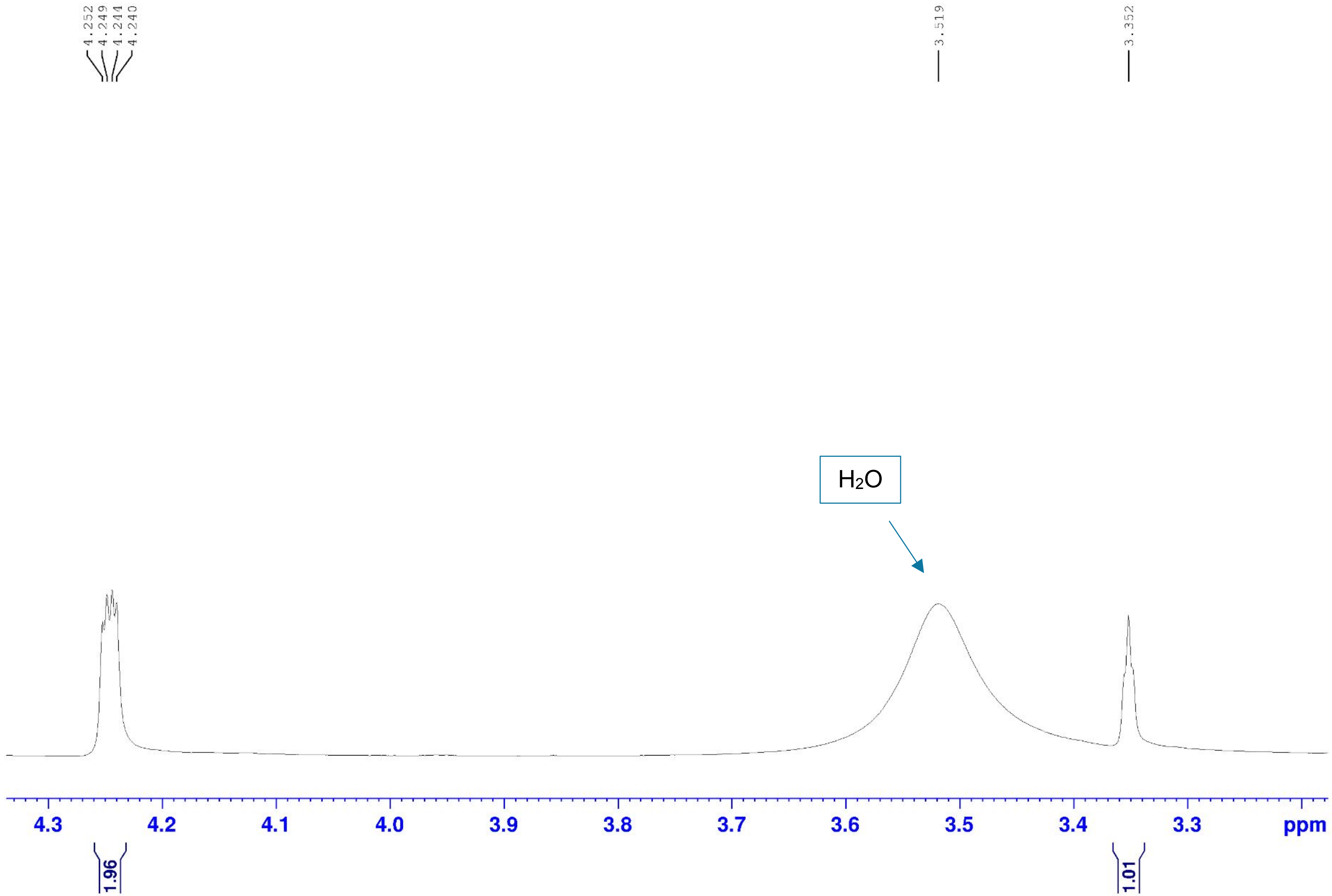

Fig. S10 ^{1}H-NMR spectrum of **[L1PtCl]PF$_6$** in DMSO-d$_6$ (600 MHz)

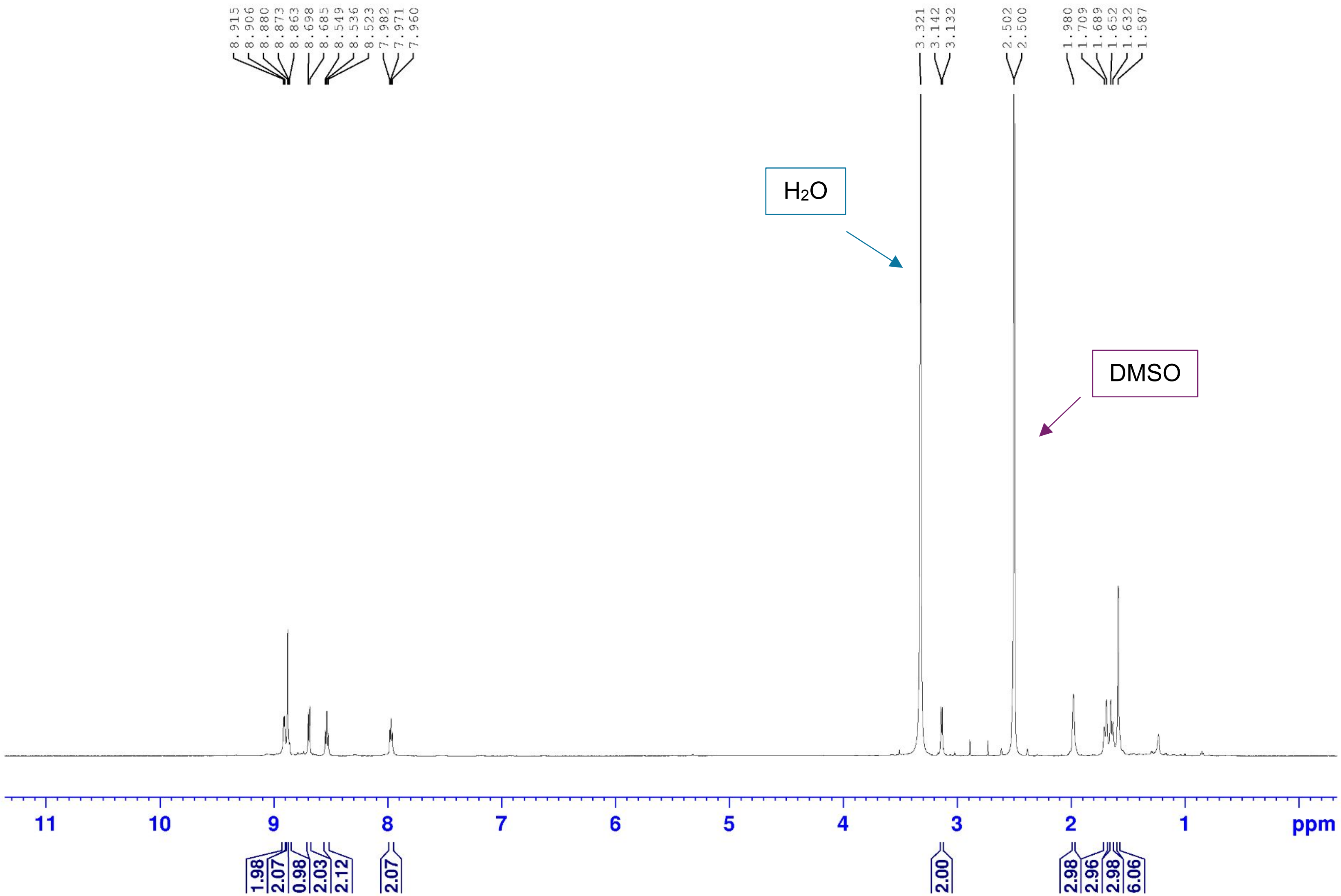

Fig. S11 ^{1}H-NMR spectrum of **[L2PtCl]PF$_6$** in DMSO-d$_6$ (600 MHz)

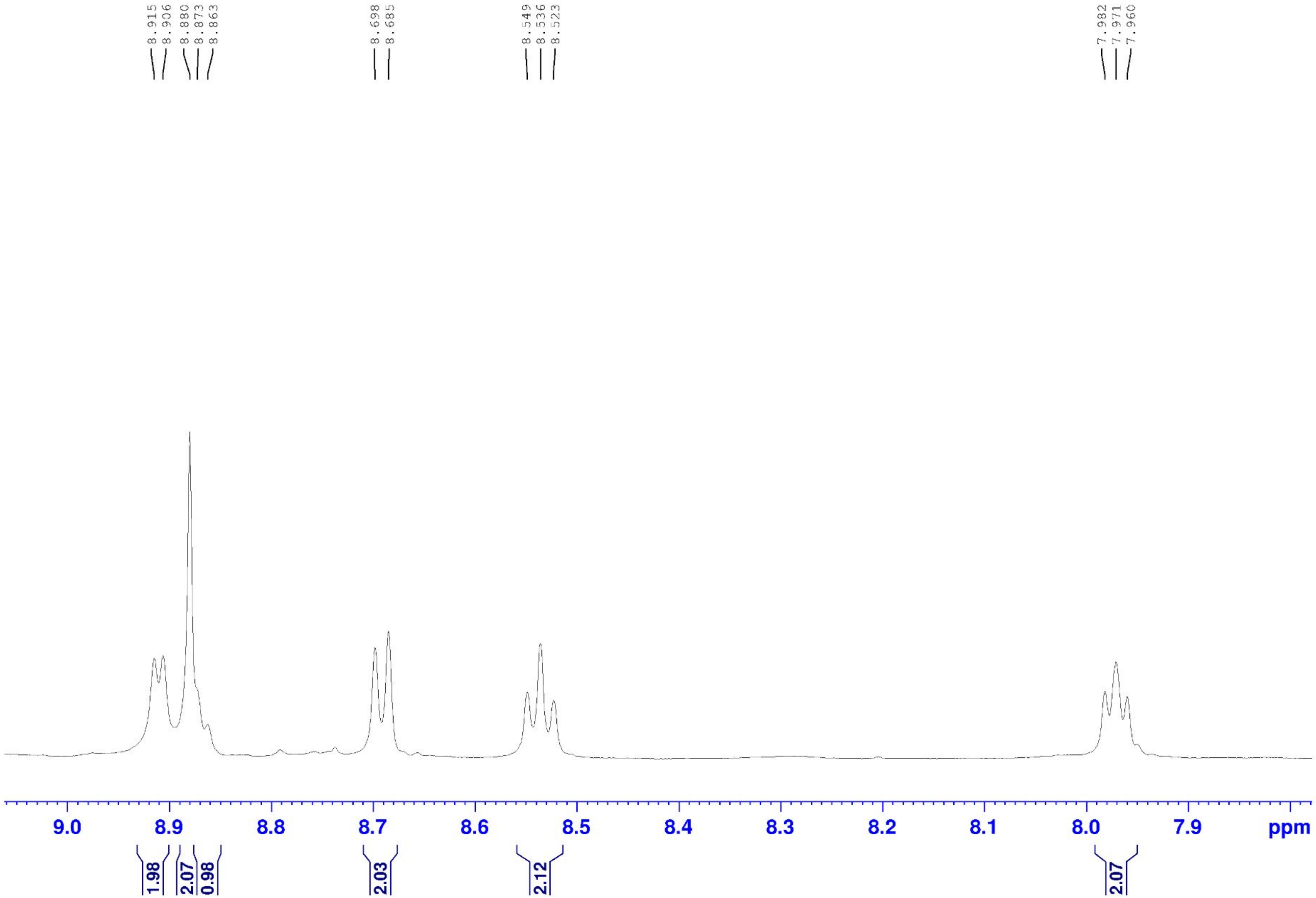

Fig. S12 ^{1}H-NMR spectrum of **[L2PtCl]PF$_6$** in DMSO-d$_6$ (600 MHz)

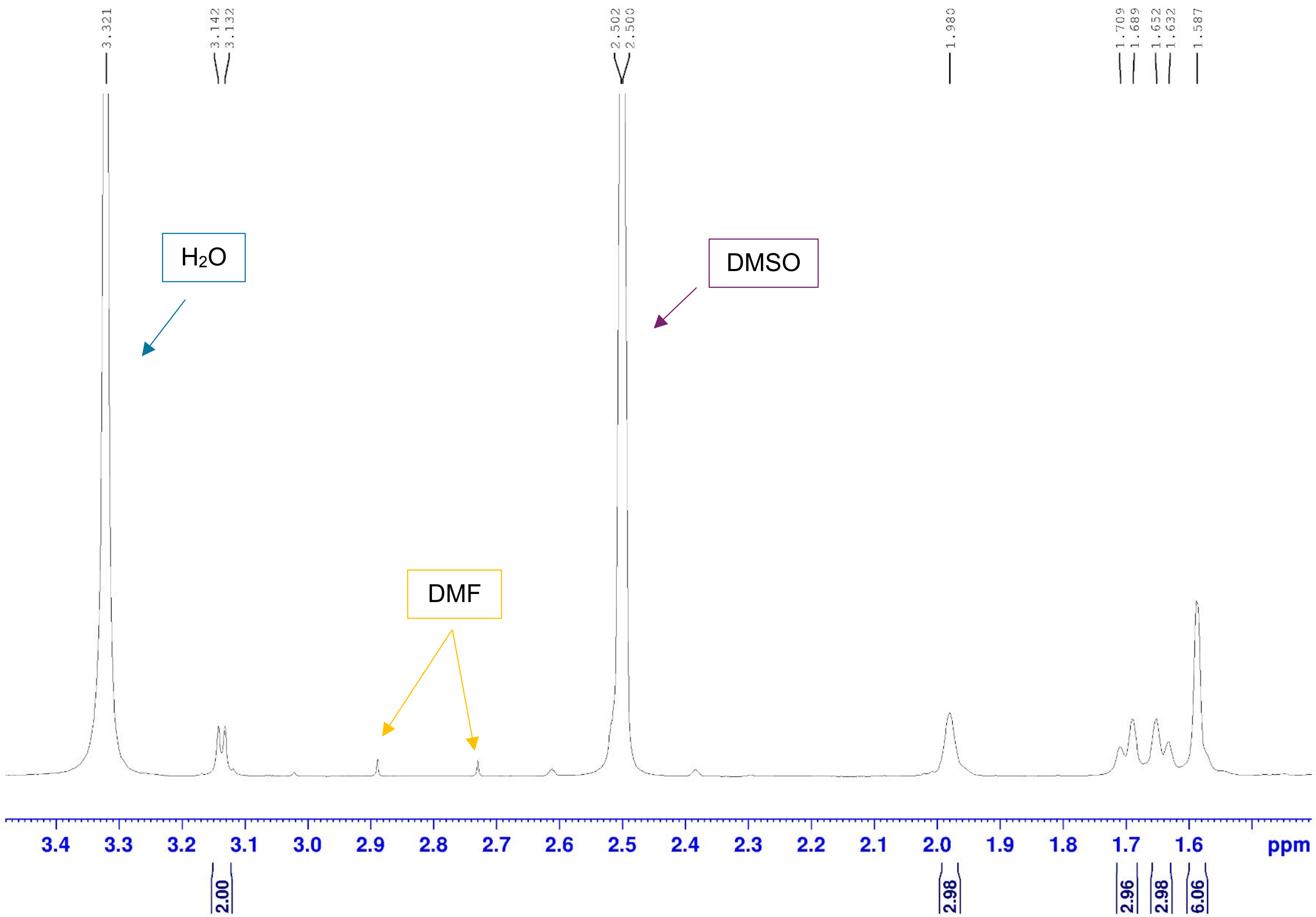

Fig. S13 ^{1}H-NMR spectrum of **[L2PtCl]PF$_6$** in DMSO-d$_6$ (600 MHz)

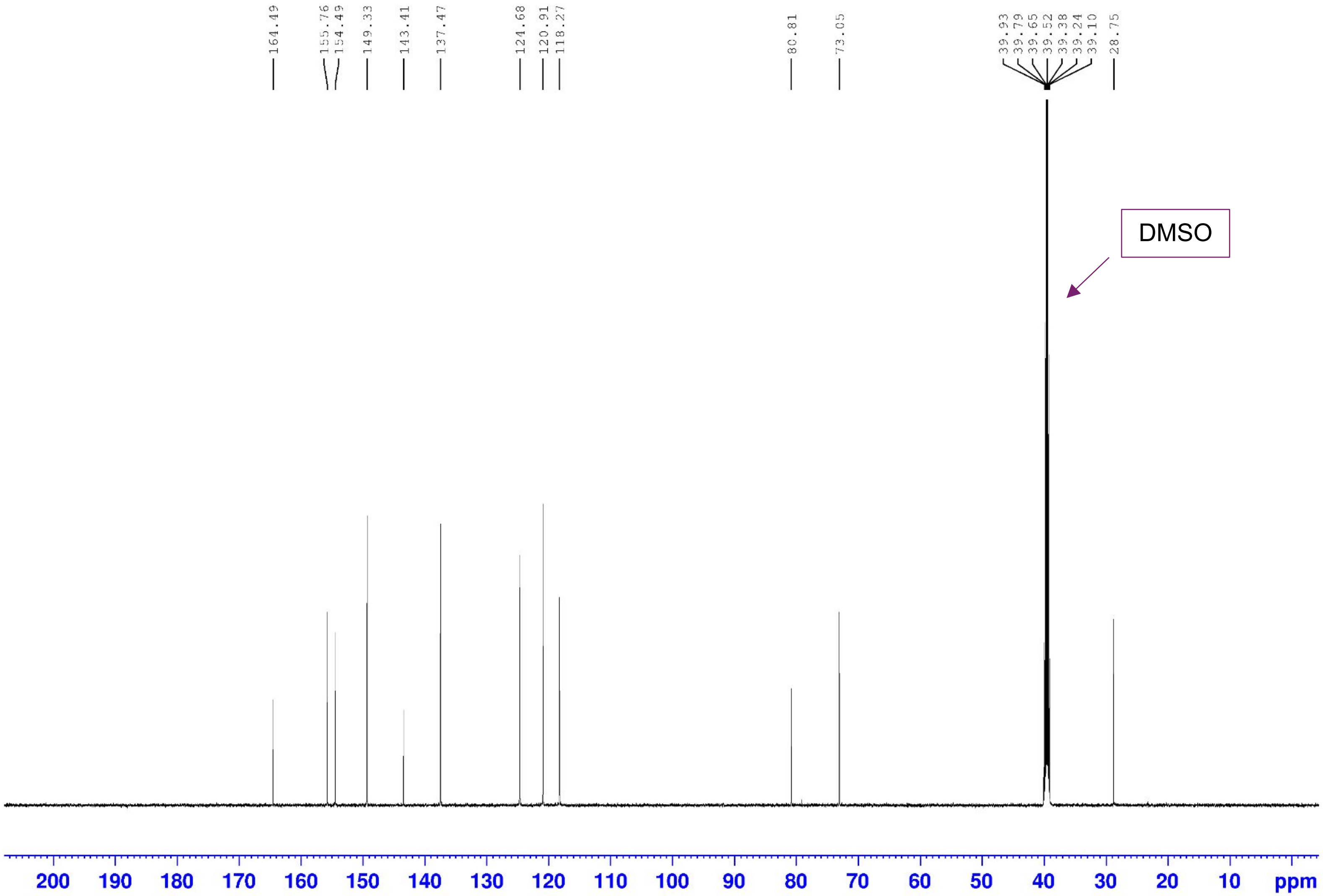

Fig. S14 ^{13}C-NMR spectrum of **L1** in DMSO-d6 (600 MHz)

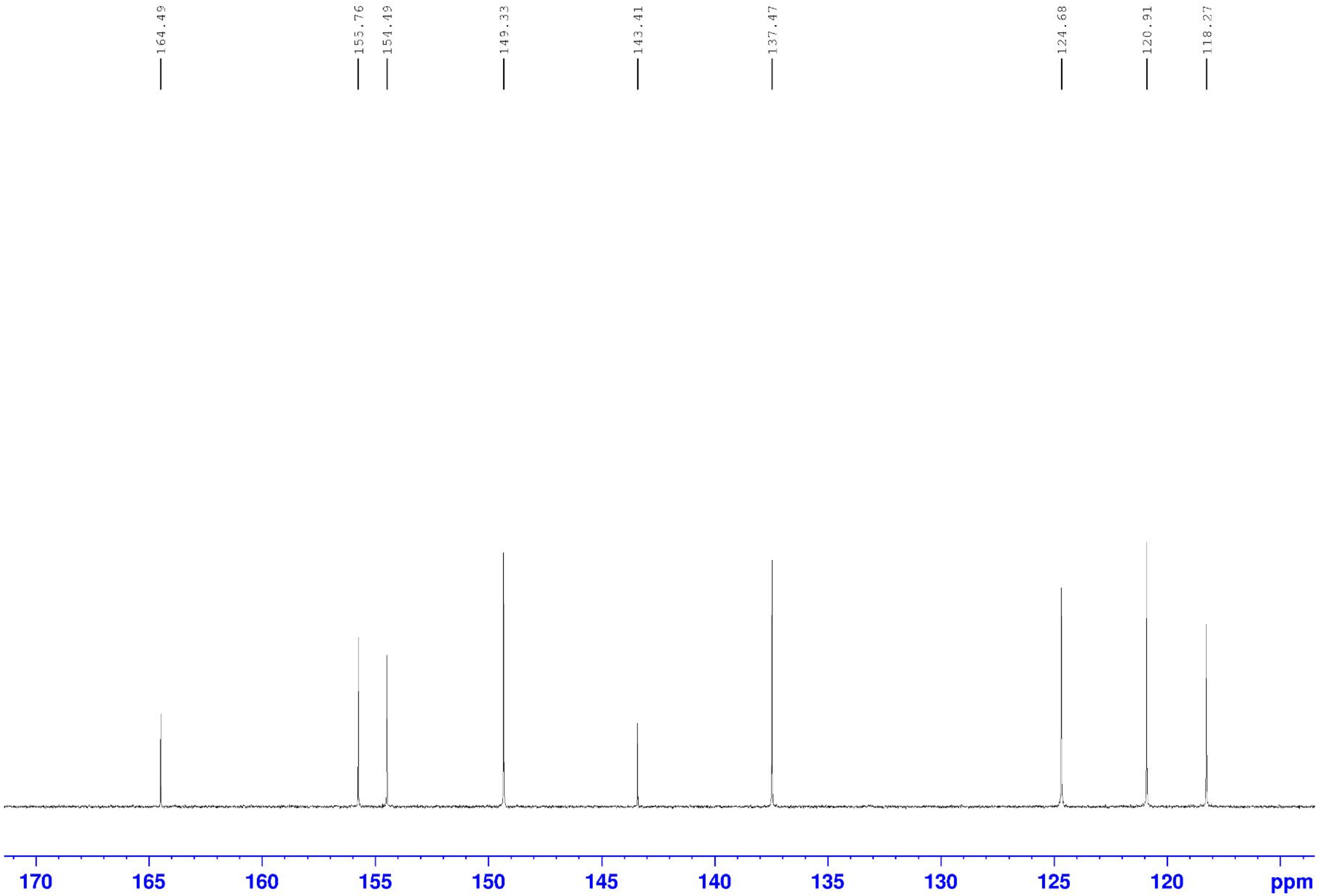

Fig. S15 ^{13}C-NMR spectrum of **L1** in DMSO-d6 (600 MHz)

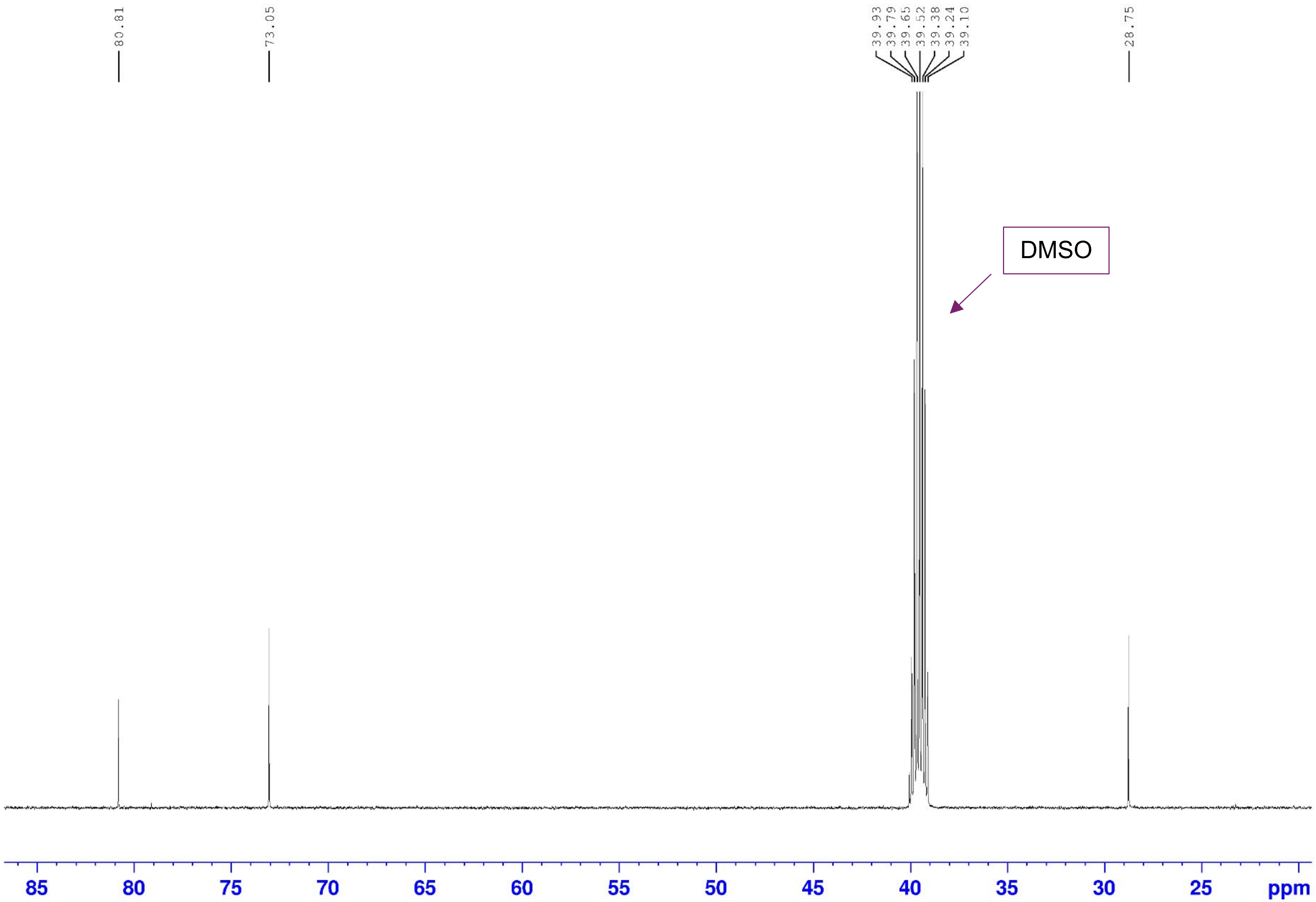

Fig. S16 ^{13}C -NMR spectrum of **L1** in DMSO-d6 (600 MHz)

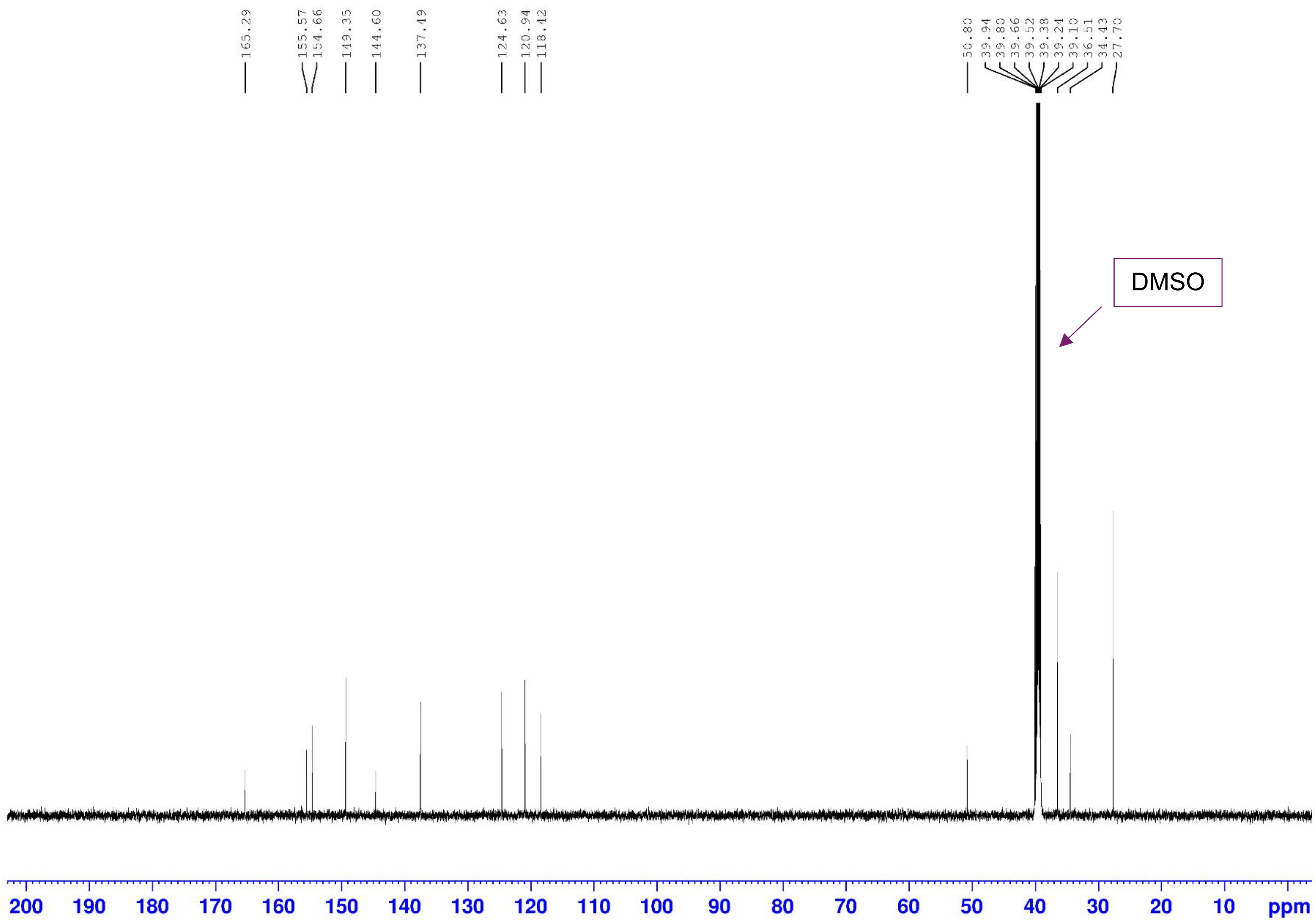

Fig. S17 ^{13}C-NMR spectrum of **L2** in DMSO-d6 (600 MHz)

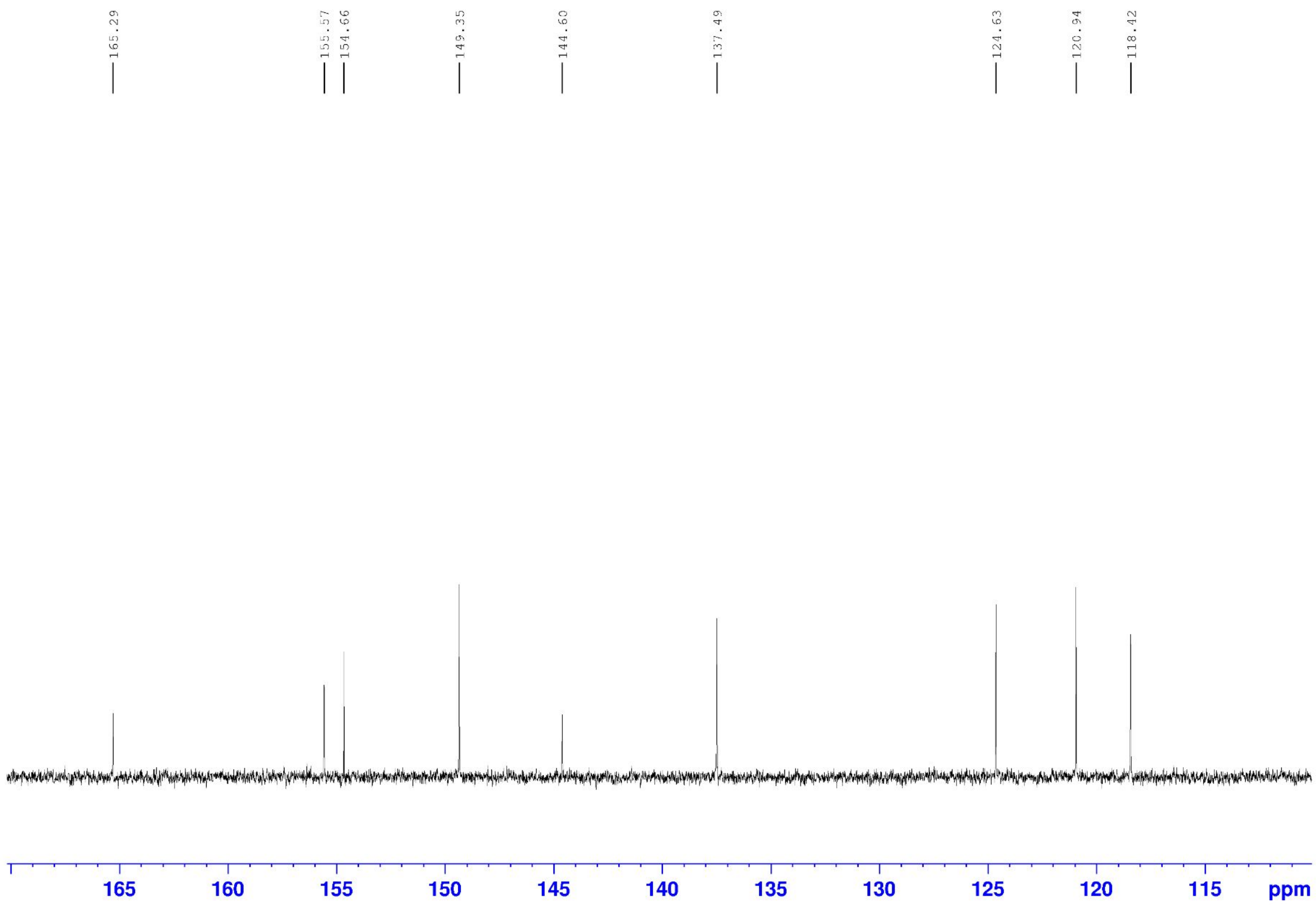

Fig. S18 ^{13}C-NMR spectrum of **L2** in DMSO-d6 (600 MHz)

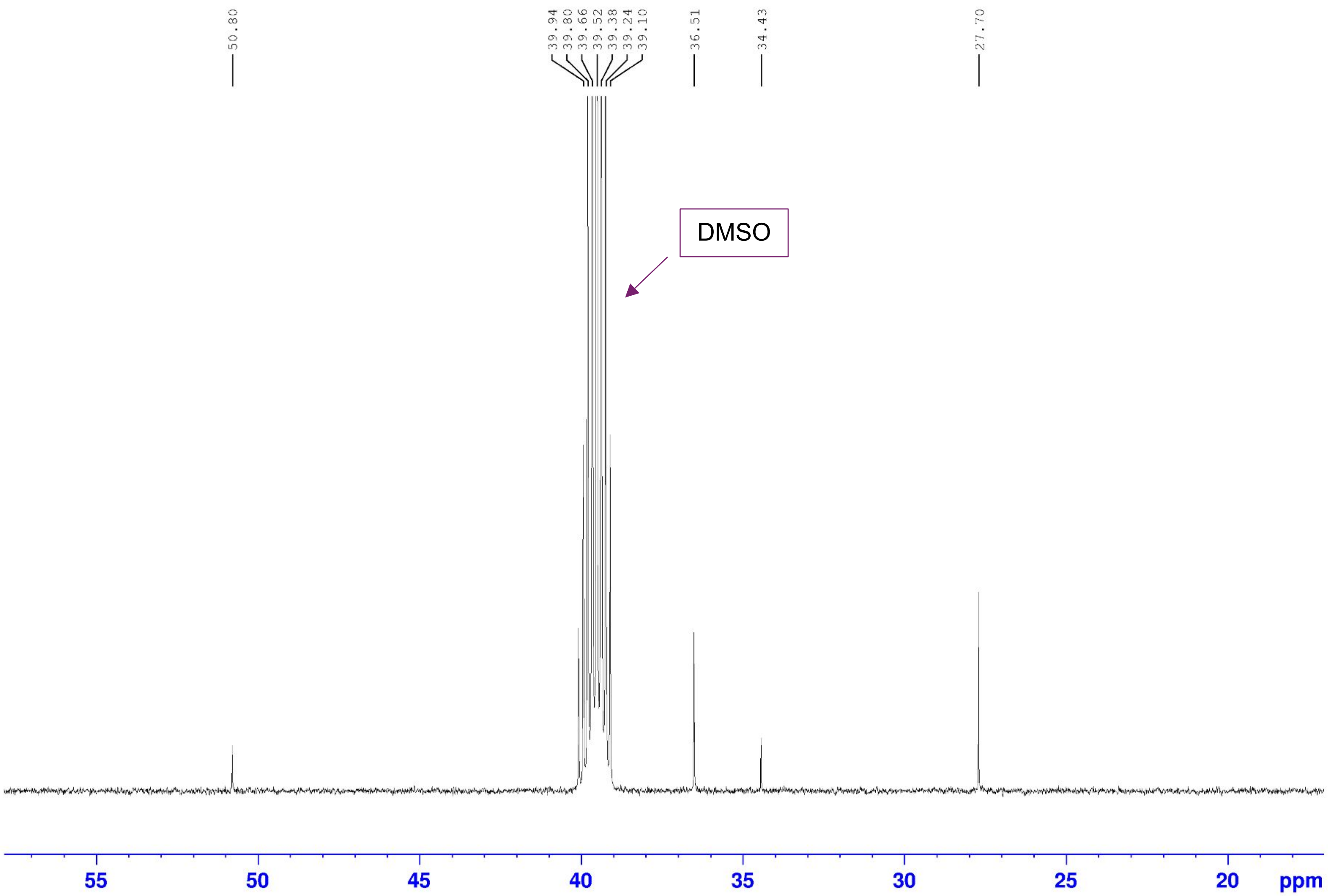

Fig. S19 ^{13}C -NMR spectrum of **L2** in DMSO-d6 (600 MHz)

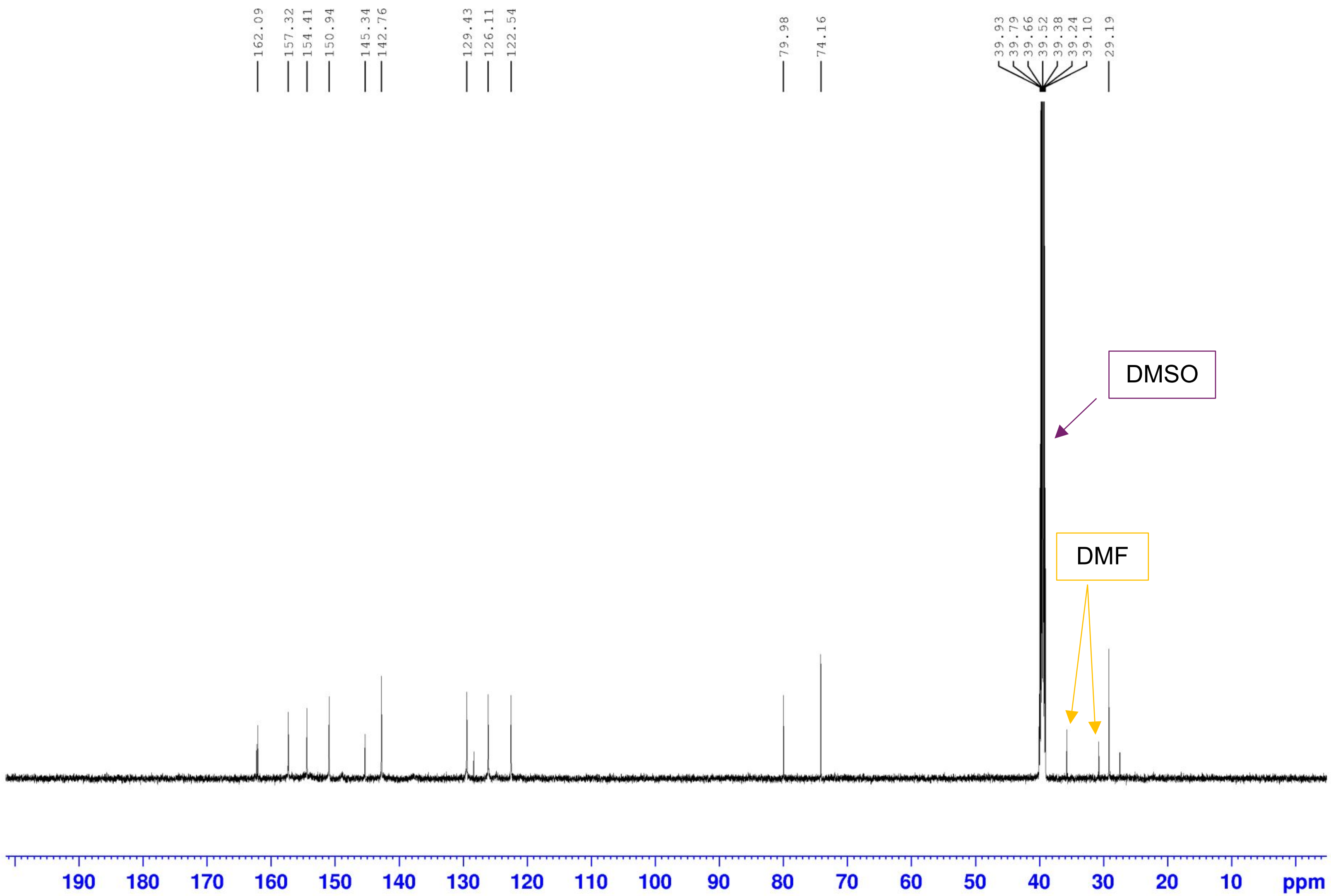

Fig. S20 ^{13}C -NMR spectrum of **[L1PtCl]PF$_6$** in DMSO-d6 (600 MHz)

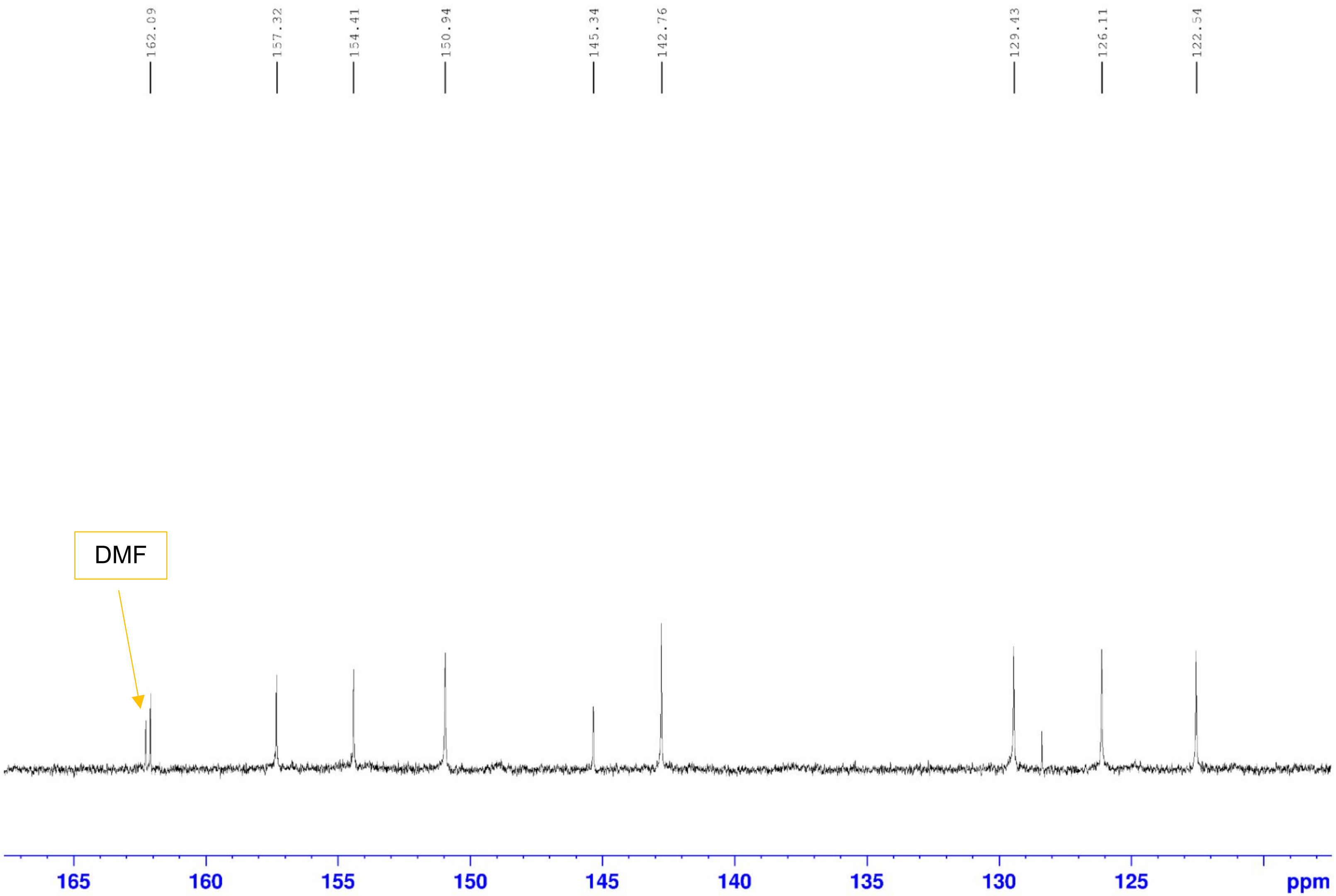

Fig. S21 ^{13}C -NMR spectrum of **[L1PtCl]PF$_6$** in DMSO-d6 (600 MHz)

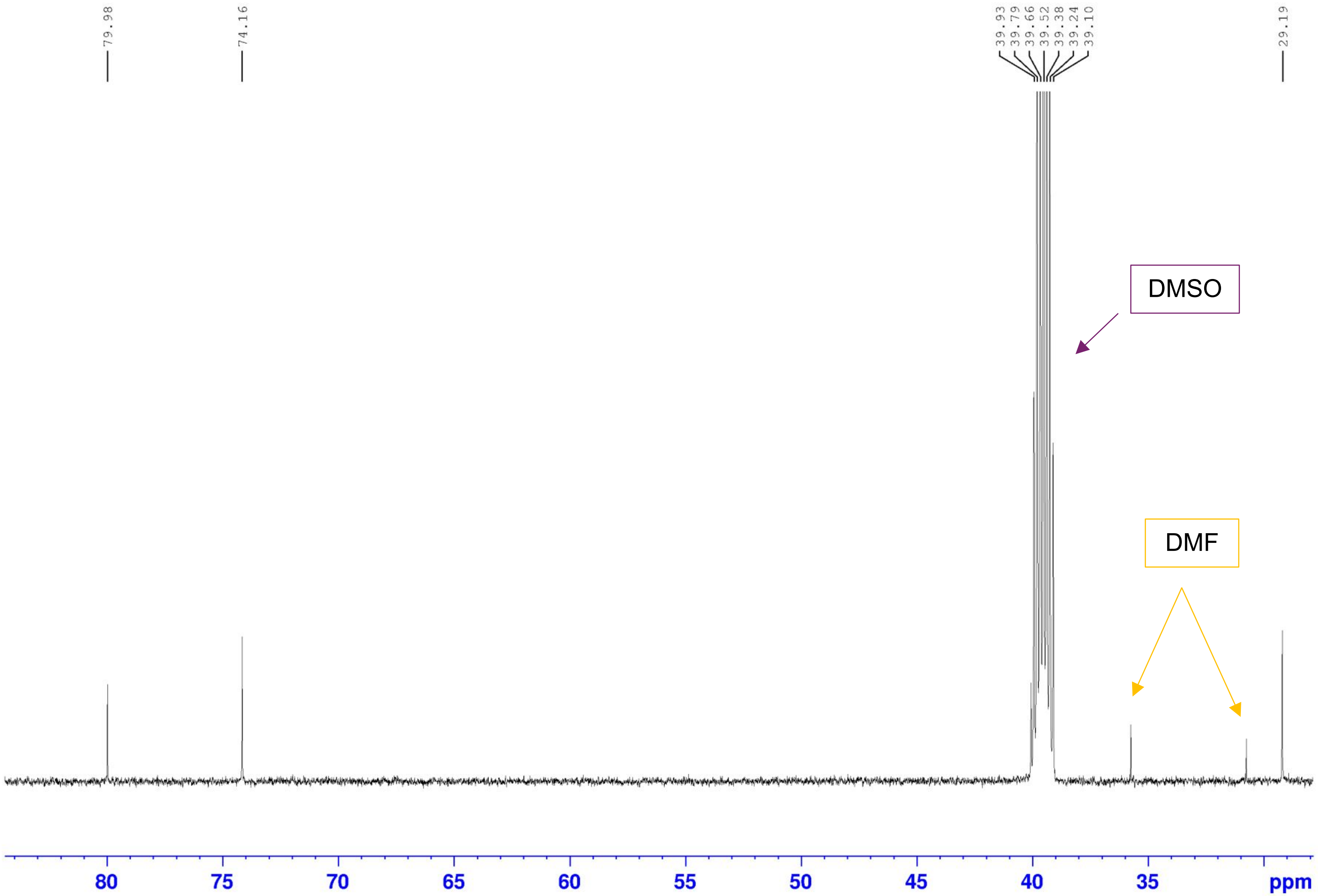

Fig. S22 ^{13}C -NMR spectrum of **[L1PtCl]PF$_6$** in DMSO-d6 (600 MHz)

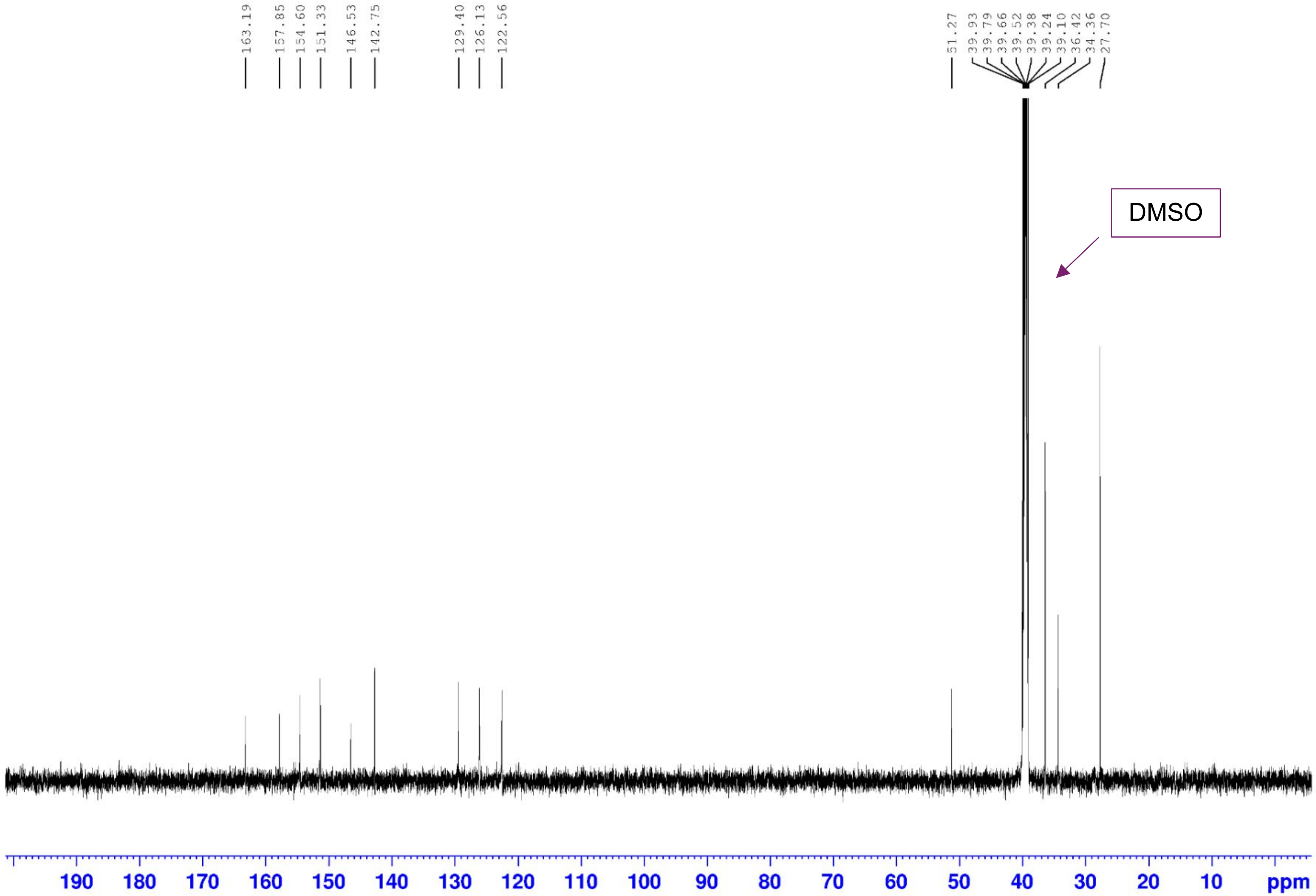

Fig. S23 ^{13}C -NMR spectrum of **[L2PtCl]PF$_6$** in DMSO-d6 (600 MHz)

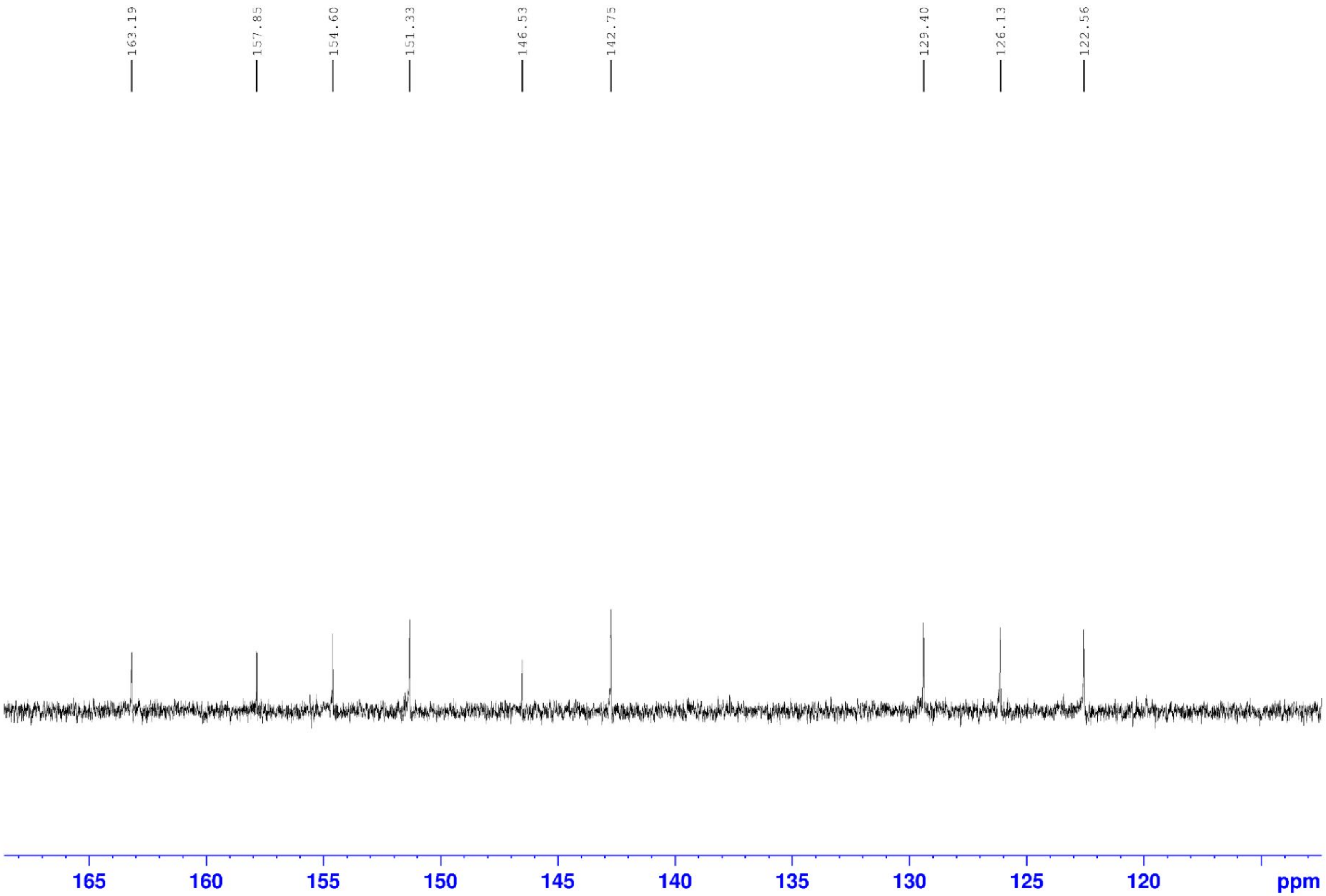

Fig. S24 ^{13}C -NMR spectrum of **[L2PtCl]PF$_6$** in DMSO-d6 (600 MHz)

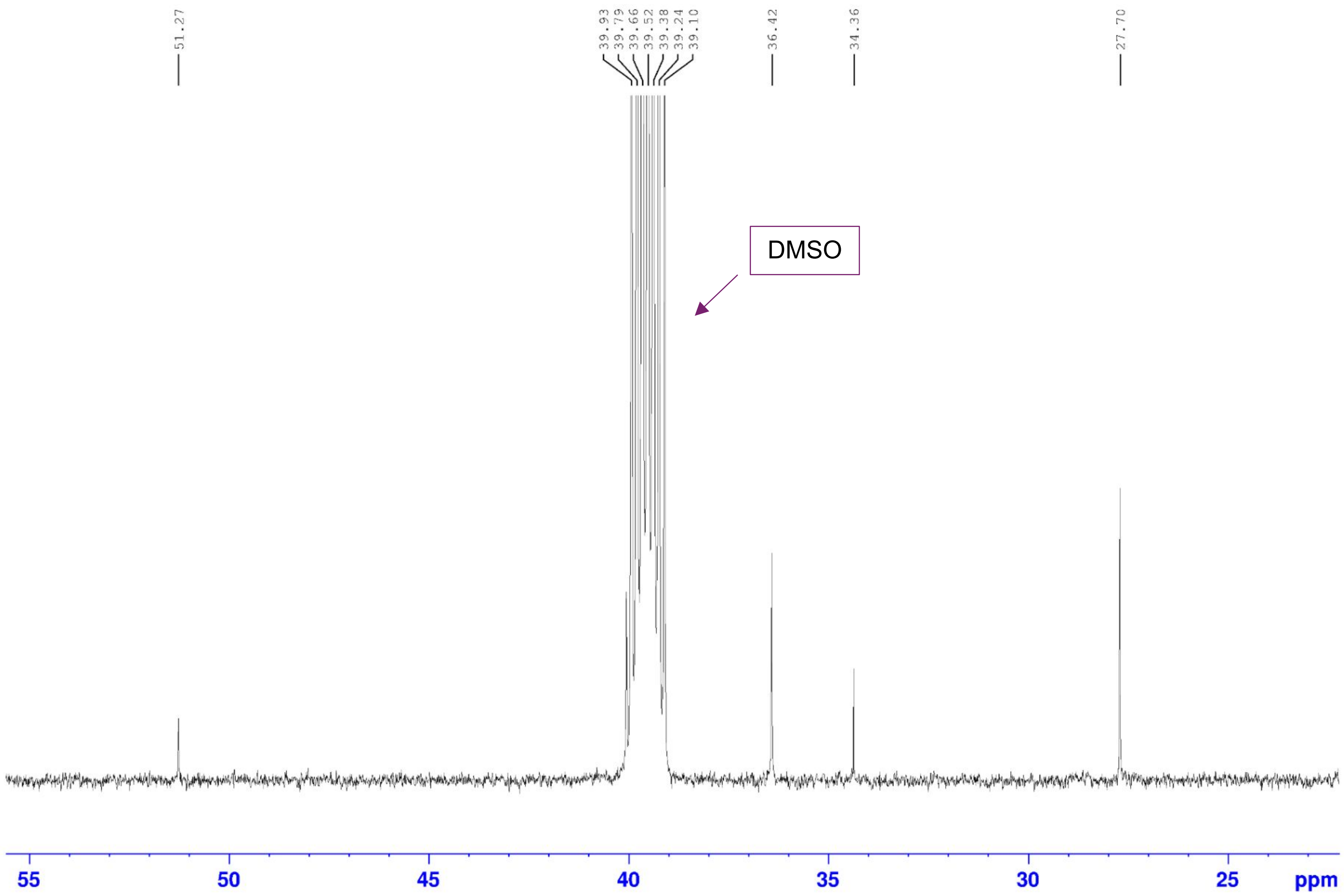

Fig. S25 ^{13}C -NMR spectrum of **[L2PtCl]PF$_6$** in DMSO-d6 (600 MHz)

Single Mass Analysis

Tolerance = 5.0 PPM / DBE: min = -1.5, max = 60.0
Element prediction: Off
Number of isotope peaks used for i-FIT = 9

Monoisotopic Mass, Even Electron Ions
99 formula(e) evaluated with 1 results within limits (all results (up to 1000) for each mass)
Elements Used:
C: 0-50 H: 0-50 N: 0-5 O: 0-3

250221_L1_A 31 (0.328) Cm (31:42-7:8) TOF MS ES+ 1.89e+006

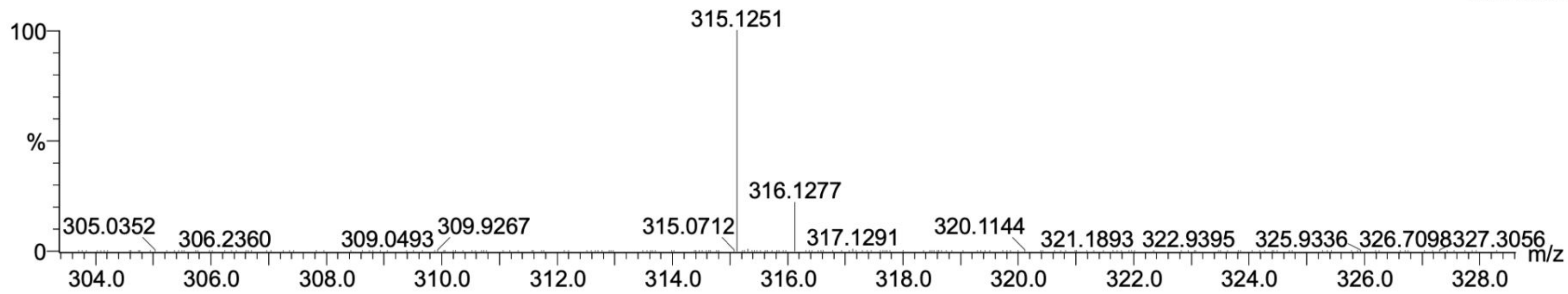

		mDa	PPM	DBE				
Minimum:				-1.5				
Maximum:		5.0	5.0	60.0				
Mass	Calc. Mass	mDa	PPM	DBE	i-FIT	Norm	Conf(%)	Formula
315.1251	315.1246	0.5	1.6	14.5	727.7	n/a	n/a	C19 H15 N4 O

Fig. S26 HRMS of **L1**

Single Mass Analysis
Tolerance = 5.0 PPM / DBE: min = -1.5, max = 60.0
Element prediction: Off
Number of isotope peaks used for i-FIT = 9

Monoisotopic Mass, Even Electron Ions
104 formula(e) evaluated with 1 results within limits (all results (up to 1000) for each mass)
Elements Used:
C: 0-50 H: 0-50 N: 0-5 O: 0-3

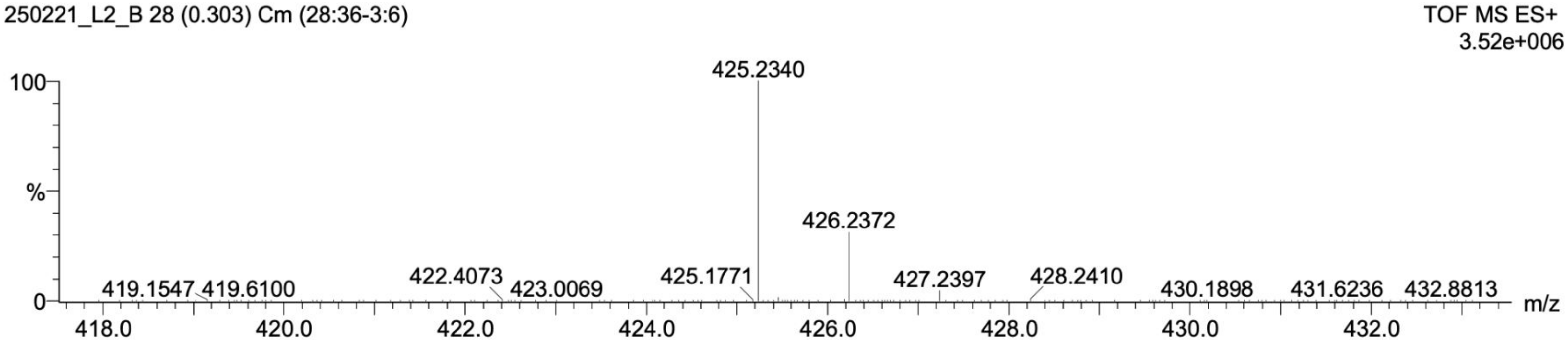

		mDa	PPM	DBE				
Minimum:				-1.5				
Maximum:		5.0	5.0	60.0				
Mass	Calc. Mass	mDa	PPM	DBE	i-FIT	Norm	Conf(%)	Formula
425.2340	425.2341	-0.1	-0.2	15.5	1001.6	n/a	n/a	C27 H29 N4 O

Fig. S27 HRMS of **L2**

Single Mass Analysis

Tolerance = 5.0 PPM / DBE: min = -1.5, max = 60.0
Element prediction: Off
Number of isotope peaks used for i-FIT = 9

Monoisotopic Mass, Even Electron Ions
119 formula(e) evaluated with 1 results within limits (all results (up to 1000) for each mass)
Elements Used:
C: 0-35 H: 0-30 N: 0-5 O: 0-2 Cl: 0-1 Pt: 0-1

250221_PtL1A 30 (0.320) Cm (30:50) TOF MS ES+ 4.26e+006

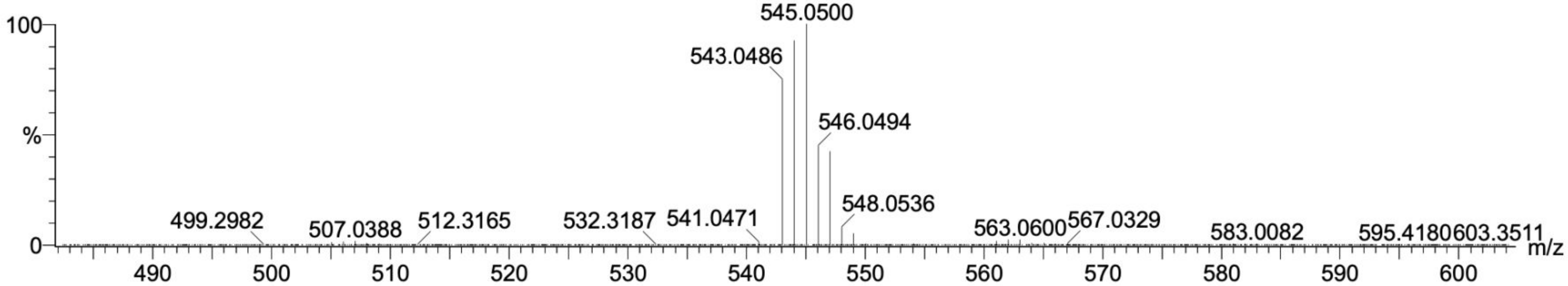

Minimum:				-1.5				
Maximum:		5.0	5.0	60.0				
Mass	Calc. Mass	mDa	PPM	DBE	i-FIT	Norm	Conf(%)	Formula
544.0507	544.0504	0.3	0.6	15.5	1092.1	n/a	n/a	C19 H14 N4 O Cl Pt

Fig. S28 HRMS of **[L1PtCl]PF$_6$**

Single Mass Analysis

Tolerance = 5.0 PPM / DBE: min = -1.5, max = 60.0
Element prediction: Off
Number of isotope peaks used for i-FIT = 9

Monoisotopic Mass, Even Electron Ions
124 formula(e) evaluated with 1 results within limits (all results (up to 1000) for each mass)
Elements Used:
C: 0-35 H: 0-30 N: 0-5 O: 0-2 Cl: 0-1 Pt: 0-1

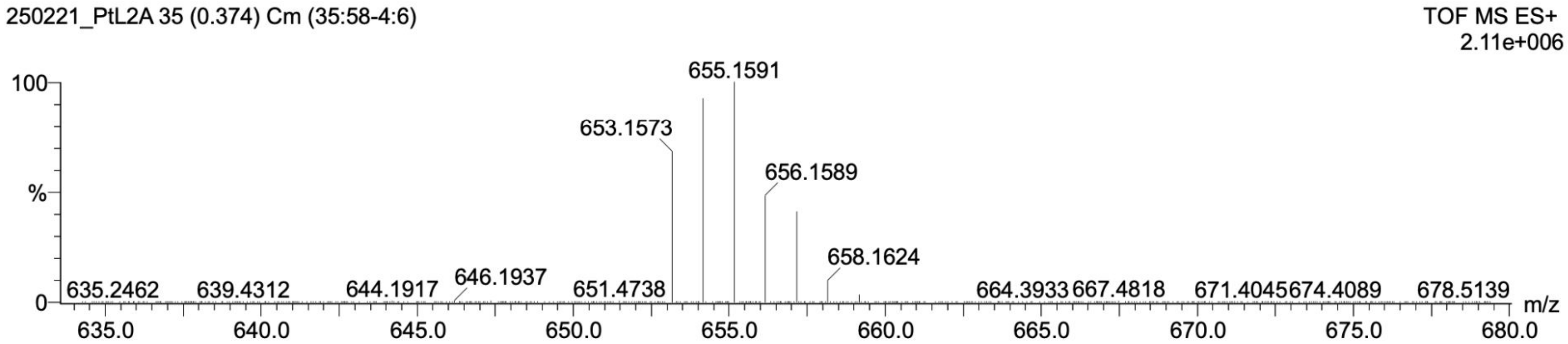

Minimum:				-1.5				
Maximum:		5.0	5.0	60.0				
Mass	Calc. Mass	mDa	PPM	DBE	i-FIT	Norm	Conf(%)	Formula
654.1598	654.1599	-0.1	-0.2	16.5	1224.6	n/a	n/a	C27 H28 N4 O Cl Pt

Fig. S29 HRMS of **[L2PtCl]PF_6**

Single Mass Analysis

Tolerance = 5.0 PPM / DBE: min = -1.5, max = 60.0
Element prediction: Off
Number of isotope peaks used for i-FIT = 9

Monoisotopic Mass, Even Electron Ions
124 formula(e) evaluated with 1 results within limits (all results (up to 1000) for each mass)
Elements Used:
C: 0-35 H: 0-30 N: 0-5 O: 0-2 Cl: 0-1 Pt: 0-1

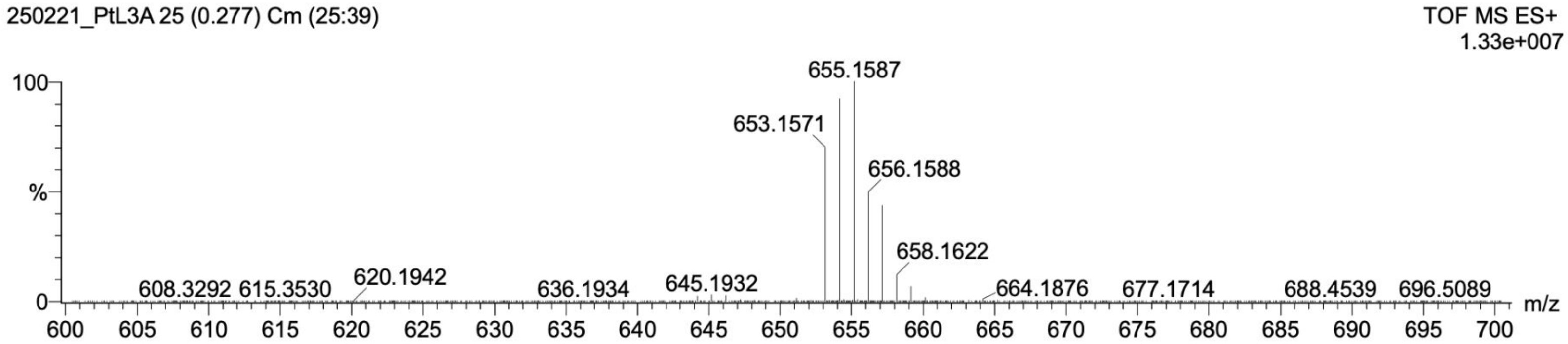

Minimum:				-1.5				
Maximum:		5.0	5.0	60.0				
Mass	Calc. Mass	mDa	PPM	DBE	i-FIT	Norm	Conf(%)	Formula
654.1594	654.1599	-0.5	-0.8	16.5	1543.9	n/a	n/a	C27 H28 N4 O Cl Pt

Fig. S30 HRMS of **[L2PtCl]$PF_6\cdot H_2O$**

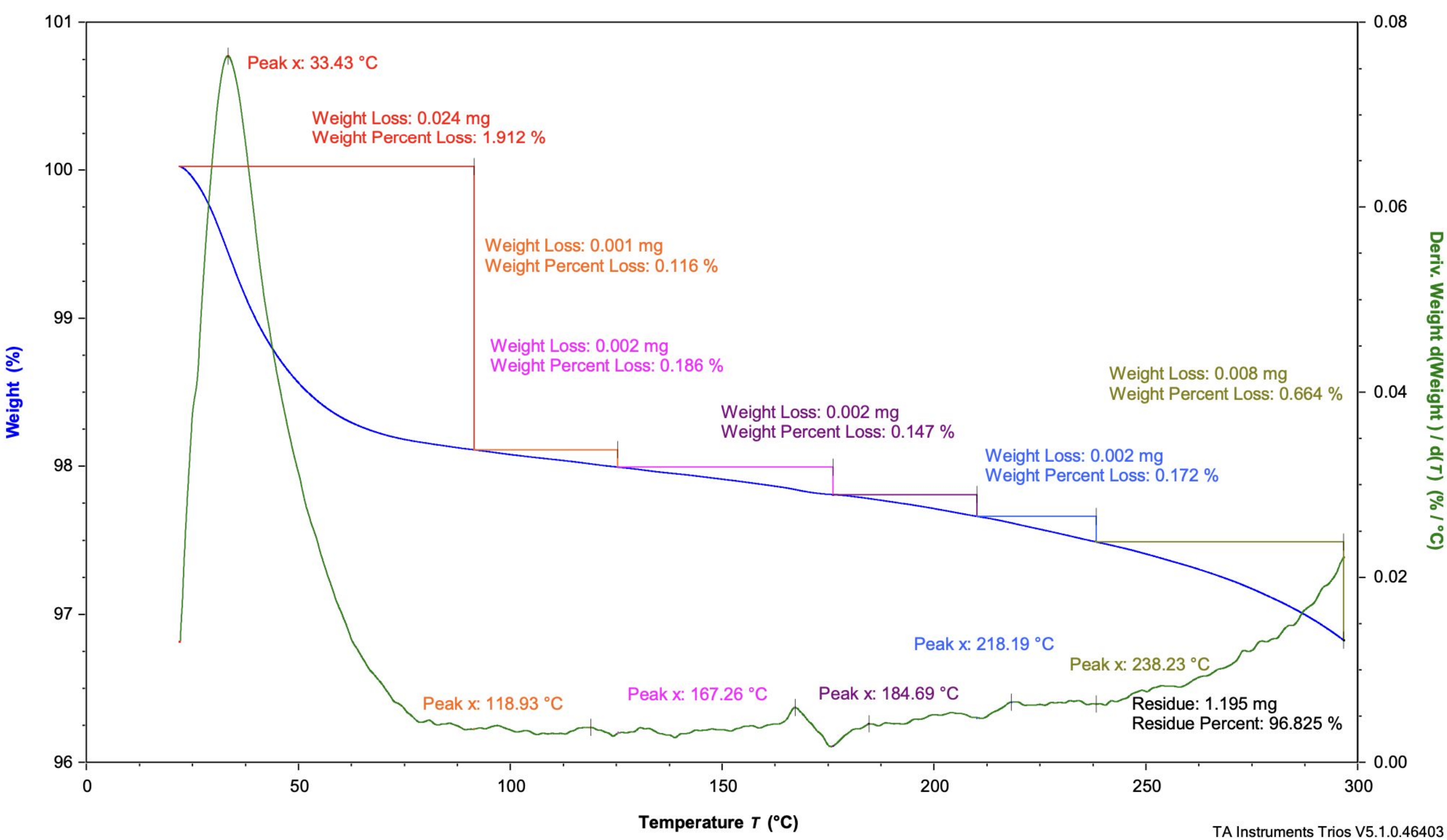

Fig. S31 TGA of **[L2PtCl]PF$_6$·H$_2$O** (10K/min to 300 ^{o}C; N_2 purging)

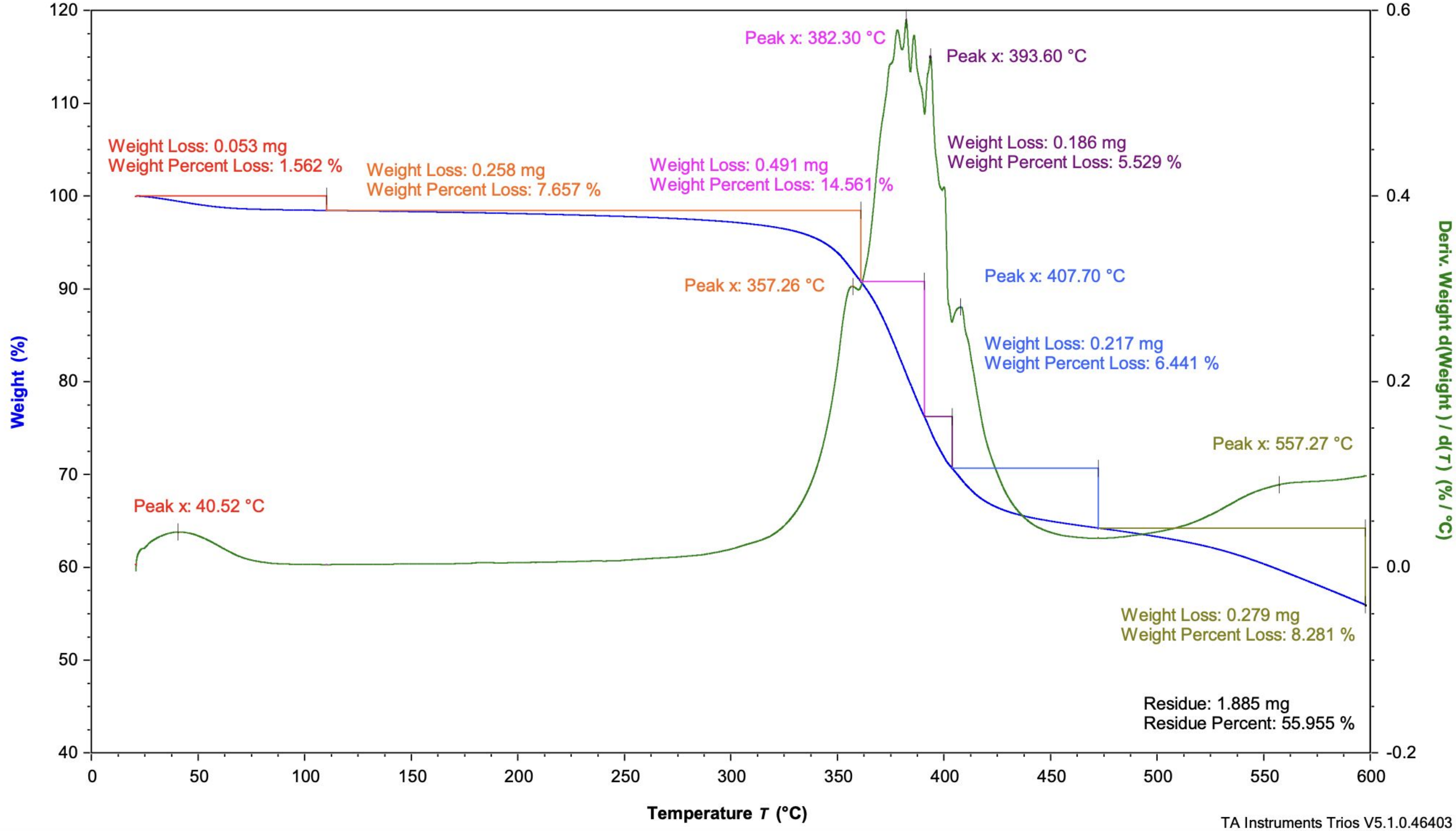

Fig. S32 TGA of **[L2PtCl]PF$_6$·H$_2$O** (10K/min to 600 ^{o}C; N_2 purging)

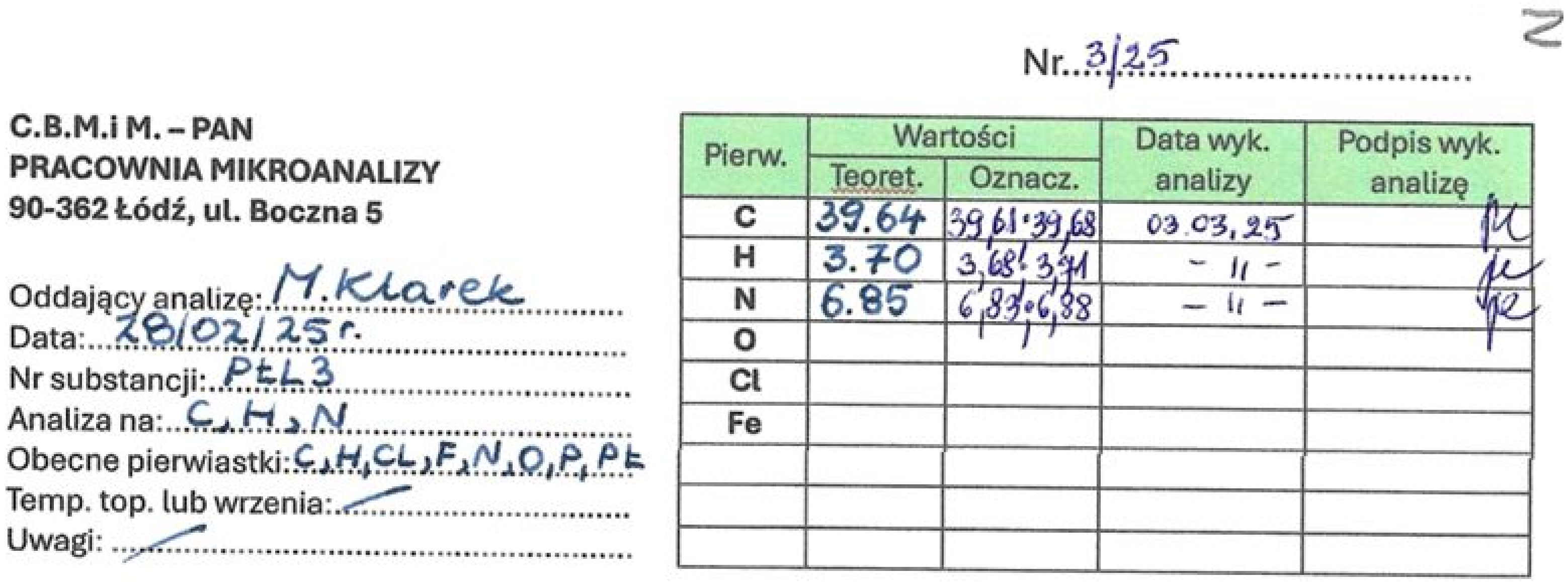

Nr. 3/25

C.B.M.i M. – PAN
PRACOWNIA MIKROANALIZY
90-362 Łódź, ul. Boczna 5

Oddający analizę: M. Klarek
Data: 28/02/25 r.
Nr substancji: PtL3
Analiza na: C, H, N
Obecne pierwiastki: C, H, Cl, F, N, O, P, Pt
Temp. top. lub wrzenia: —
Uwagi: —

Pierw.	Wartości		Data wyk. analizy	Podpis wyk. analizę
	Teoret.	Oznacz.		
C	39.64	39,61; 39,68	03.03.25	[illegible]
H	3.70	3,68; 3,71	– " –	[illegible]
N	6.85	6,83; 6,88	– " –	[illegible]
O				
Cl				
Fe				

Fig. S33 Elemental analysis results for **[L2PtCl]PF$_6$·H$_2$O** (symbol "PtL3" pertains to our laboratory numbering scheme)

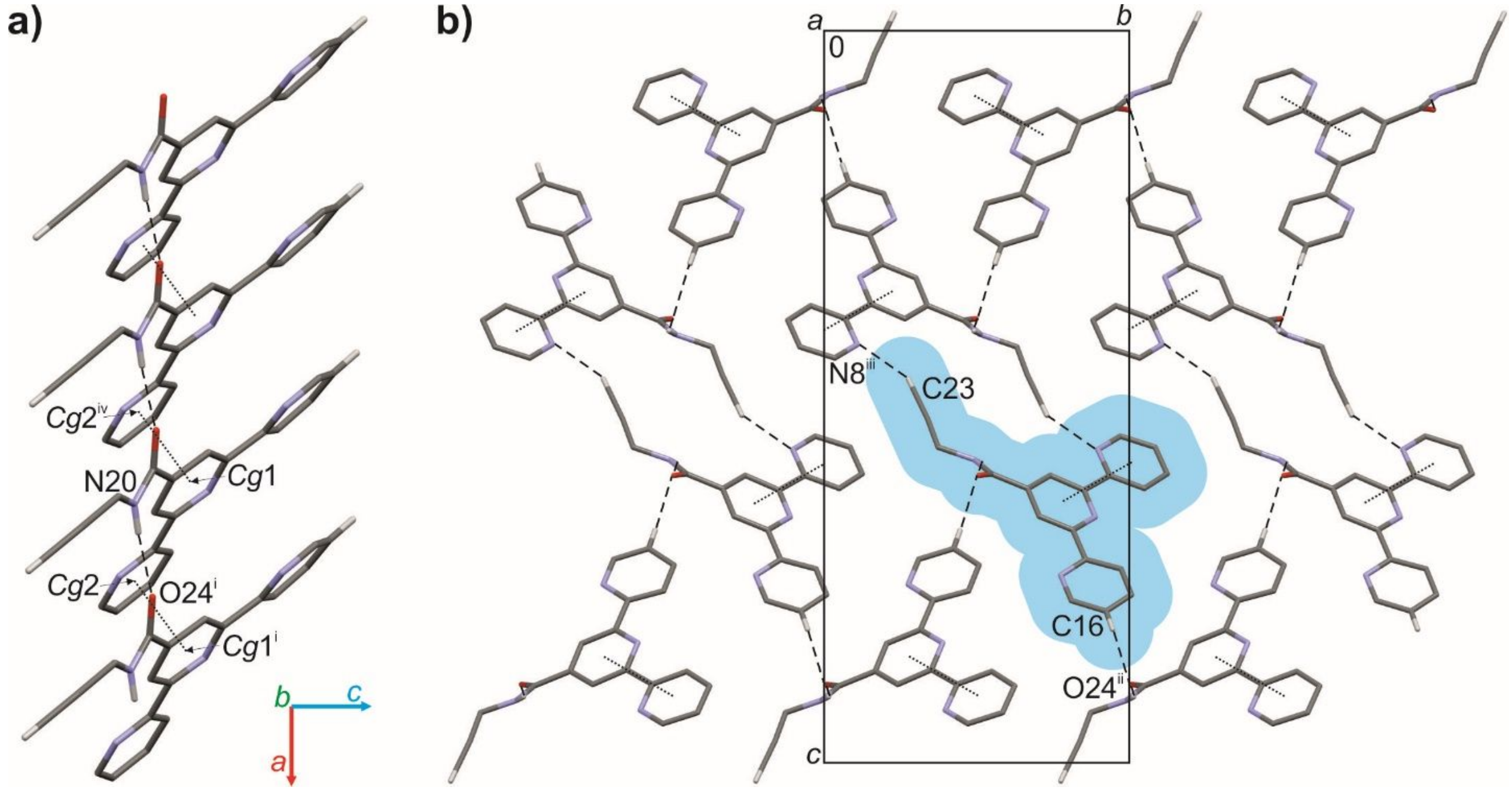

Fig. S34 Supramolecular architecture in the crystal of **L1**, where: (a) infinite chain of molecules interacting via the N−H···O hydrogen bonds and the π-π contacts; (b) general view on packing of molecules, shown along the *a*-direction (single chain of molecules is highlighted in blue). The N−H···O, C−H···O and C−H···O interactions are represented by dashed lines, while dotted lines represent the π-π contacts. The H-atoms not involved in intermolecular interactions were omitted for clarity. Symmetry codes: (i) $x - 1, y, z$;; (ii) $-x + 1/2, y + 1/2, -z + 3/2$; (iii) $-x + 2, -y + 1, -z + 1$; (iv) $x + 1, y, z$.

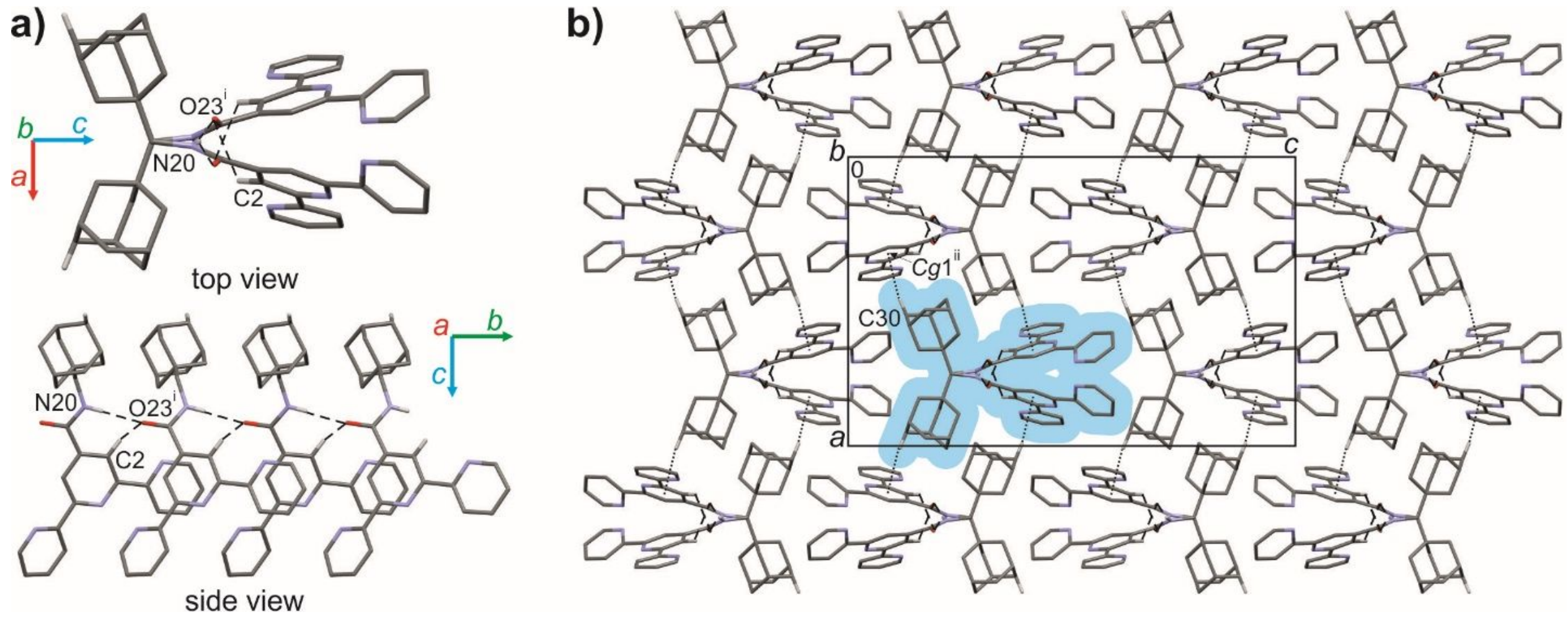

Fig. S35 Supramolecular architecture in the crystal of **L2**, where: (a) infinite chain of molecules interacting *via* the N−H···O and the C−H···O hydrogen bonds viewed from two different perspectives; (b) general view on packing of molecules, shown along the *b*-direction (single chain of molecules is highlighted in blue). The N−H···O and C−H···O interactions are represented by dashed lines, while dotted lines represent the C−H···π contacts. The H-atoms not involved in intermolecular interactions were omitted for clarity. Symmetry codes: (i) $-x + 3/2$, $y + 1/2$, z; (ii) $x - 1/2$, y, $-z + 1/2$.

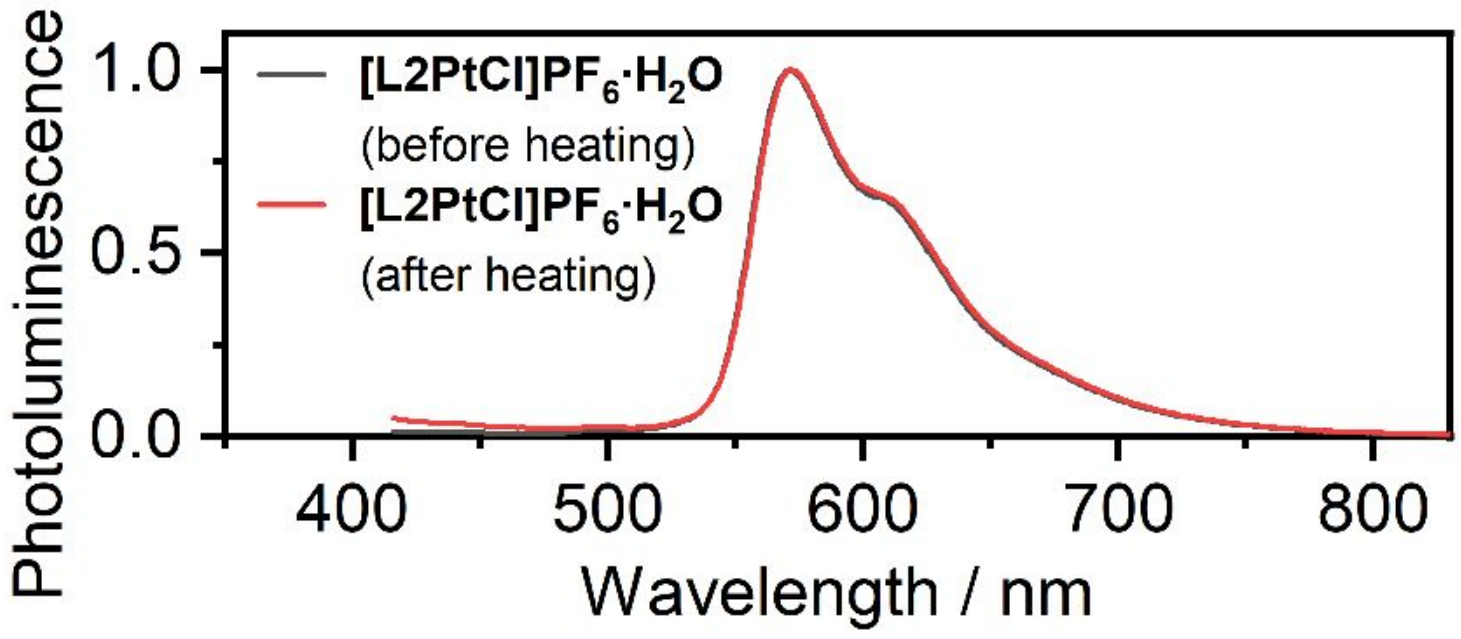

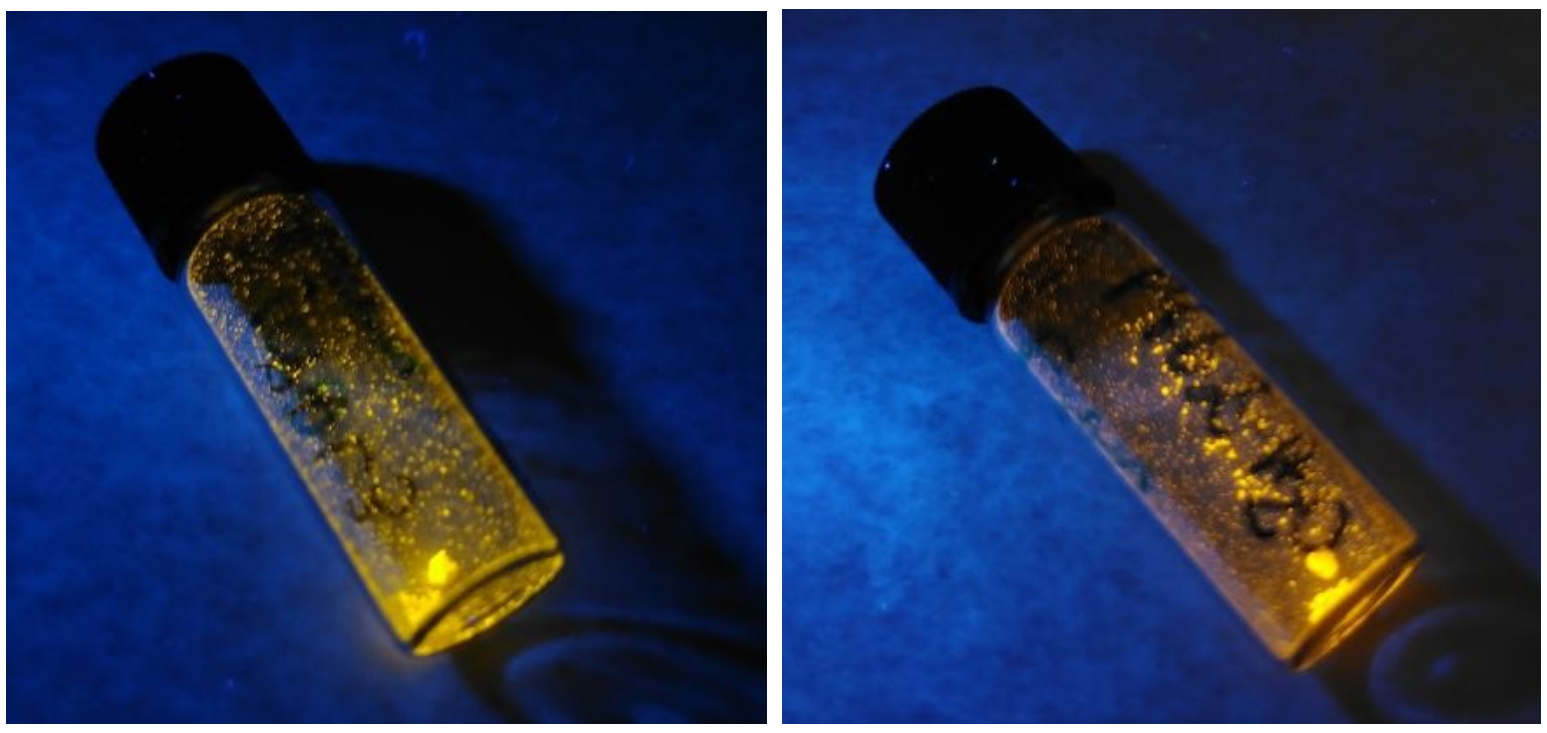

(*left*) **[L2PtCl]PF$_6$·H$_2$O** before heating

(*right*) **[L2PtCl]PF$_6$·H$_2$O** after heating with a heat gun > 100°C

Fig. S36 Photoluminescence before and after heating a sample of **[L2PtCl]PF$_6$·H$_2$O**

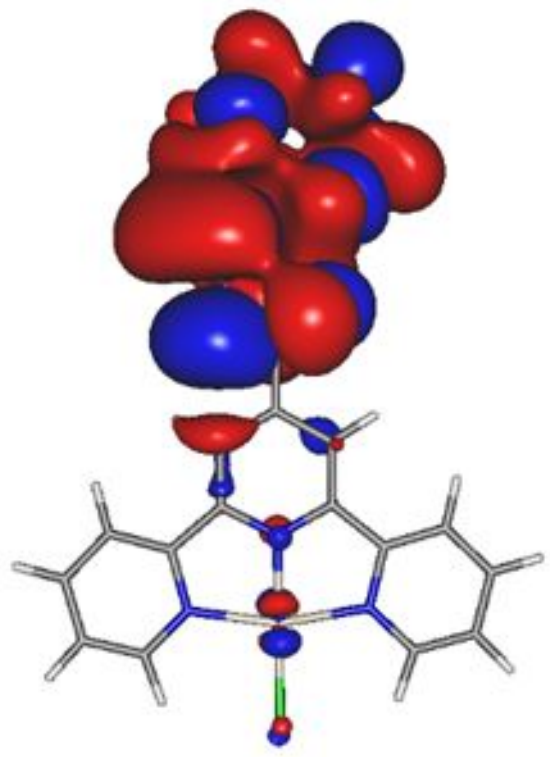

Fig. S37 HOMO of **[L2PtCl]PF$_6$** at the T_1 geometry with an iso-value of 0.01.

Table S1. Crystal data and structure refinement for **L1** and **L2**.

**Compound**	**L1**	**L2**
Empirical formula	$C_{19}H_{14}N_4O$	$C_{27}H_{28}N_4O$
Formula weight	314.34	424.53
Temperature/K	100(2)	100(2)
Crystal system	monoclinic	orthorhombic
Space group	$P2_1/n$	*Pbca*
a/Å	4.88635(9)	16.8412(6)
b/Å	11.5478(2)	9.7727(2)
c/Å	27.7053(5)	26.1141(5)
α/°	90	90
β/°	91.2885(16)	90
γ/°	90	90
Volume/Å^3	1562.92(5)	4298.0(2)
Z	4	8
ρ_{calc}g/cm^3	1.336	1.312
μ/mm^{-1}	0.694	0.641
F(000)	656.0	1808.0
Crystal size/mm^3	0.46 × 0.04 × 0.04	0.14 × 0.02 × 0.02
Radiation	Cu$K\alpha$ (λ = 1.54184)	Cu $K\alpha$ (λ = 1.54184)
2Θ range for data collection/°	8.296 to 134.14	6.77 to 134.154
Index ranges	$-5 \le h \le 5$, $-13 \le k \le 13$, $-33 \le l \le 33$	$-20 \le h \le 19$, $-11 \le k \le 10$, $-31 \le l \le 17$
Reflections collected	13070	18650
Independent reflections	2779 [R_{int} = 0.0362, R_{sigma} = 0.0291]	3833 [R_{int} = 0.0860, R_{sigma} = 0.0706]
Data/restraints/parameters	2779/0/220	3833/0/292
Goodness-of-fit on F^2	1.042	1.033
Final R indexes [I>=2σ (I)]	R_1 = 0.0349, wR_2 = 0.0924	R_1 = 0.0507, wR_2 = 0.1080
Final R indexes [all data]	R_1 = 0.0395, wR_2 = 0.0959	R_1 = 0.0886, wR_2 = 0.1223

Table S2. Bond lengths for **L1** [Å].

Atom	Atom	Length/Å	Atom	Atom	Length/Å
C1	C2	1.3898(16)	C10	C11	1.3837(18)
C1	C6	1.3869(16)	C11	C12	1.3866(18)
C1	C19	1.5020(16)	C13	C18	1.3928(17)
C2	C3	1.3978(17)	C13	N14	1.3468(16)
C3	C7	1.4944(16)	C15	C16	1.3826(19)
C3	N4	1.3400(15)	C15	N14	1.3374(17)
C5	C6	1.3929(17)	C16	C17	1.3867(19)
C5	C13	1.4882(16)	C17	C18	1.3843(18)
C5	N4	1.3469(16)	C19	N20	1.3343(15)
C7	C12	1.3947(17)	C19	O24	1.2381(14)
C7	N8	1.3445(15)	C21	C22	1.4633(17)
C9	C10	1.3861(18)	C21	N20	1.4559(16)
C9	N8	1.3391(16)	C22	C23	1.1865(19)

Table S3. Values of valence angles for **L1**.

**Atom**	**Atom**	**Atom**	**Angle/°**	**Atom**	**Atom**	**Atom**	**Angle/°**
C2	C1	C19	123.54(11)	C11	C12	C7	118.70(11)
C6	C1	C2	118.98(11)	C18	C13	C5	121.85(11)
C6	C1	C19	117.45(10)	N14	C13	C5	115.50(11)
C1	C2	C3	118.29(11)	N14	C13	C18	122.60(11)
C2	C3	C7	120.10(10)	N14	C15	C16	124.20(13)
N4	C3	C2	123.13(11)	C15	C16	C17	117.87(12)
N4	C3	C7	116.76(10)	C18	C17	C16	119.28(12)
C6	C5	C13	119.55(11)	C17	C18	C13	118.73(12)
N4	C5	C6	122.45(11)	N20	C19	C1	117.52(10)
N4	C5	C13	117.97(10)	O24	C19	C1	119.96(10)
C1	C6	C5	119.08(11)	O24	C19	N20	122.53(11)
C12	C7	C3	121.02(11)	N20	C21	C22	113.12(10)
N8	C7	C3	116.42(10)	C23	C22	C21	175.43(13)
N8	C7	C12	122.56(11)	C3	N4	C5	118.04(10)
N8	C9	C10	123.77(11)	C9	N8	C7	117.57(10)
C11	C10	C9	118.16(11)	C15	N14	C13	117.28(11)
C10	C11	C12	119.23(11)	C19	N20	C21	118.83(10)

Table S4. Values of torsion angles for **L1** [°].

A	B	C	D	Angle/°	A	B	C	D	Angle/°
C1	C2	C3	C7	-177.06(10)	C10	C11	C12	C7	-0.37(17)
C1	C2	C3	N4	1.52(17)	C12	C7	N8	C9	0.54(16)
C1	C19	N20	C21	175.85(10)	C13	C5	C6	C1	179.43(10)
C2	C1	C6	C5	-0.31(16)	C13	C5	N4	C3	-179.24(9)
C2	C1	C19	N20	30.57(16)	C15	C16	C17	C18	-0.66(18)
C2	C1	C19	O24	-149.61(11)	C16	C15	N14	C13	0.66(19)
C2	C3	C7	C12	175.82(10)	C16	C17	C18	C13	2.09(17)
C2	C3	C7	N8	-3.75(15)	C18	C13	N14	C15	0.90(17)
C2	C3	N4	C5	-0.11(16)	C19	C1	C2	C3	176.68(10)
C3	C7	C12	C11	-179.58(10)	C19	C1	C6	C5	-178.37(10)
C3	C7	N8	C9	-179.89(10)	C22	C21	N20	C19	154.28(11)
C5	C13	C18	C17	175.09(10)	N4	C3	C7	C12	-2.84(15)
C5	C13	N14	C15	-176.62(11)	N4	C3	C7	N8	177.58(9)
C6	C1	C2	C3	-1.25(16)	N4	C5	C6	C1	1.81(17)
C6	C1	C19	N20	-151.47(11)	N4	C5	C13	C18	12.48(16)
C6	C1	C19	O24	28.35(15)	N4	C5	C13	N14	-169.98(10)
C6	C5	C13	C18	-165.25(11)	N8	C7	C12	C11	-0.03(17)
C6	C5	C13	N14	12.30(15)	N8	C9	C10	C11	0.27(18)
C6	C5	N4	C3	-1.59(16)	N14	C13	C18	C17	-2.28(17)
C7	C3	N4	C5	178.51(9)	N14	C15	C16	C17	-0.8(2)
C9	C10	C11	C12	0.26(17)	O24	C19	N20	C21	-3.97(17)
C10	C9	N8	C7	-0.66(17)					

Table S5. Bond lengths for **L2** [Å].

Atom	Atom	Length/Å	Atom	Atom	Length/Å
C1	C2	1.383(3)	C16	C17	1.378(4)
C1	C6	1.393(3)	C17	C18	1.387(3)
C1	C19	1.508(3)	C19	N20	1.346(3)
C2	C3	1.393(3)	C19	O32	1.240(3)
C3	C7	1.488(3)	C21	C22	1.525(3)
C3	N4	1.346(3)	C21	N20	1.461(3)
C5	C6	1.396(3)	C22	C23	1.536(3)
C5	C13	1.491(3)	C22	C27	1.535(3)
C5	N4	1.344(3)	C22	C28	1.546(3)
C7	C12	1.390(3)	C23	C24	1.537(3)
C7	N8	1.346(3)	C24	C25	1.528(3)
C9	C10	1.383(3)	C24	C31	1.538(3)
C9	N8	1.336(3)	C25	C26	1.530(3)
C10	C11	1.388(3)	C26	C27	1.537(3)
C11	C12	1.383(3)	C26	C30	1.540(3)
C13	C18	1.395(3)	C28	C29	1.537(3)
C13	N14	1.337(3)	C29	C30	1.530(3)
C15	C16	1.383(4)	C29	C31	1.532(3)
C15	N14	1.345(3)			

Table S6. Values of valence angles for **L2**.

Atom	Atom	Atom	Angle/°	Atom	Atom	Atom	Angle/°
C2	C1	C6	118.7(2)	O32	C19	N20	123.5(2)
C2	C1	C19	122.9(2)	N20	C21	C22	115.61(19)
C6	C1	C19	118.3(2)	C21	C22	C23	111.79(18)
C1	C2	C3	119.3(2)	C21	C22	C27	111.3(2)
C2	C3	C7	120.7(2)	C21	C22	C28	107.85(19)
N4	C3	C2	122.6(2)	C23	C22	C28	107.71(18)
N4	C3	C7	116.71(19)	C27	C22	C23	109.7(2)
C6	C5	C13	120.5(2)	C27	C22	C28	108.36(18)
N4	C5	C6	123.2(2)	C22	C23	C24	110.23(18)
N4	C5	C13	116.21(19)	C23	C24	C31	109.5(2)
C1	C6	C5	118.4(2)	C25	C24	C23	109.8(2)
C12	C7	C3	121.6(2)	C25	C24	C31	109.20(18)
N8	C7	C3	115.85(19)	C24	C25	C26	109.9(2)
N8	C7	C12	122.6(2)	C25	C26	C27	108.77(19)
N8	C9	C10	124.1(2)	C25	C26	C30	109.77(19)
C9	C10	C11	117.8(2)	C27	C26	C30	109.2(2)
C12	C11	C10	119.3(2)	C22	C27	C26	110.6(2)
C11	C12	C7	118.8(2)	C29	C28	C22	110.4(2)
C18	C13	C5	120.5(2)	C30	C29	C28	109.3(2)
N14	C13	C5	116.0(2)	C30	C29	C31	109.2(2)
N14	C13	C18	123.5(2)	C31	C29	C28	110.02(19)
N14	C15	C16	123.3(2)	C29	C30	C26	109.46(18)
C17	C16	C15	119.1(2)	C29	C31	C24	109.19(19)
C16	C17	C18	118.8(2)	C5	N4	C3	117.67(19)
C17	C18	C13	118.2(2)	C9	N8	C7	117.43(19)
N20	C19	C1	116.3(2)	C13	N14	C15	117.1(2)
O32	C19	C1	120.1(2)	C19	N20	C21	121.31(19)

Table S7. Values of torsion angles for **L2** [°].

A	B	C	D	Angle/°	A	B	C	D	Angle/°
C1	C2	C3	C7	-178.8(2)	C22	C23	C24	C31	61.4(2)
C1	C2	C3	N4	0.4(4)	C22	C28	C29	C30	60.2(2)
C1	C19	N20	C21	168.3(2)	C22	C28	C29	C31	-59.7(3)
C2	C1	C6	C5	0.5(4)	C23	C22	C27	C26	-58.3(2)
C2	C1	C19	N20	-25.7(3)	C23	C22	C28	C29	59.6(2)
C2	C1	C19	O32	150.3(2)	C23	C24	C25	C26	60.3(2)
C2	C3	C7	C12	173.2(2)	C23	C24	C31	C29	-59.5(3)
C2	C3	C7	N8	-4.7(3)	C24	C25	C26	C27	-60.4(2)
C2	C3	N4	C5	-2.0(3)	C24	C25	C26	C30	59.0(3)
C3	C7	C12	C11	-177.8(2)	C25	C24	C31	C29	60.8(3)
C3	C7	N8	C9	177.6(2)	C25	C26	C27	C22	59.6(2)
C5	C13	C18	C17	-179.4(2)	C25	C26	C30	C29	-59.1(3)
C5	C13	N14	C15	179.9(2)	C27	C22	C23	C24	57.3(2)
C6	C1	C2	C3	0.3(4)	C27	C22	C28	C29	-59.0(3)
C6	C1	C19	N20	159.4(2)	C27	C26	C30	C29	60.1(3)
C6	C1	C19	O32	-24.6(3)	C28	C22	C23	C24	-60.4(2)
C6	C5	C13	C18	156.9(2)	C28	C22	C27	C26	59.0(2)
C6	C5	C13	N14	-23.5(3)	C28	C29	C30	C26	-60.2(3)
C6	C5	N4	C3	2.9(4)	C28	C29	C31	C24	58.8(3)
C7	C3	N4	C5	177.2(2)	C30	C26	C27	C22	-60.1(2)
C9	C10	C11	C12	-0.3(4)	C30	C29	C31	C24	-61.2(3)
C10	C9	N8	C7	0.4(4)	C31	C24	C25	C26	-59.8(3)
C10	C11	C12	C7	0.4(4)	C31	C29	C30	C26	60.2(3)
C12	C7	N8	C9	-0.3(4)	N4	C3	C7	C12	-6.0(3)
C13	C5	C6	C1	179.6(2)	N4	C3	C7	N8	176.1(2)
C13	C5	N4	C3	-178.9(2)	N4	C5	C6	C1	-2.2(4)
C15	C16	C17	C18	0.5(4)	N4	C5	C13	C18	-21.4(3)
C16	C15	N14	C13	0.0(4)	N4	C5	C13	N14	158.3(2)
C16	C17	C18	C13	-1.0(4)	N8	C7	C12	C11	0.0(4)
C18	C13	N14	C15	-0.5(4)	N8	C9	C10	C11	-0.1(4)
C19	C1	C2	C3	-174.5(2)	N14	C13	C18	C17	1.0(4)
C19	C1	C6	C5	175.6(2)	N14	C15	C16	C17	0.0(4)
C21	C22	C23	C24	-178.76(19)	N20	C21	C22	C23	-52.5(3)
C21	C22	C27	C26	177.49(17)	N20	C21	C22	C27	70.5(2)
C21	C22	C28	C29	-179.62(19)	N20	C21	C22	C28	-170.72(19)
C22	C21	N20	C19	112.4(3)	O32	C19	N20	C21	-7.5(4)
C22	C23	C24	C25	-58.5(2)					

Table S8. Intermolecular hydrogen-bond geometry in the crystal of **L1**.

D−H···A	d(D−H) (Å)	d(H···A) Å	d(D···A) Å	<D−H···A (°)
N20−H20···O24^{i}	0.867(15)	1.960(15)	2.7898(13)	159.8(14)
C16−H16···O24^{ii}	0.95	2.45	3.3750(16)	165
C23−H23···N8^{iii}	0.95	2.41	3.2325(17)	145

Symmetry codes: (i) *x* − 1, *y*, *z*;; (ii) −*x* + 1/2, *y* + 1/2, −*z* + 3/2; (iii) −*x* +2, −*y* + 1, −*z* + 1.

Table S9. The geometry of *π*–*π* interactions in the crystal of **L1**.

***Cg*I**	***Cg*J**	***Cg*I···*Cg*J (Å)**	**Dihedral angle (°)**	**Interplanar distance (Å)**
1	2^{iv}	3.7308(6)	4.28(5)	3.2302(4)
2	1^{i}	3.7309(6)	4.28(5)	−3.3607(5)

*Cg*1 and *Cg*2 denote geometric centres of gravity of the aromatic rings delineated by the C5−C6/C1−C3/N4 and C9−C12/C7/C8 atoms, respectively. Symmetry codes: (i) *x* − 1, *y*, *z*; (iv) *x* + 1, *y*, *z*.

Table S10. Intermolecular hydrogen-bond geometry in the crystal of **L2**.

D−H···A	d(D−H) (Å)	d(H···A) Å	d(D···A) Å	<D−H···A (°)
N20−H2O···O32^{i}	0.86(3)	2.23(3)	3.080(3)	172(3)
C2−H2···O32^{i}	0.95	2.56	3.191(3)	124

Symmetry codes: (i) −*x* + 3/2, *y* + 1/2, *z*.

Table S11. The geometry of the C−H···π contacts in the crystal of **L2**.

**D−H**	***Cg*I**	**d(X··· *Cg*I) (Å)**	**<Y−X···*Cg*I (°)**
C30−H30A	1^{ii}	3.757(3)	169

*Cg*1 denotes the geometric centre of gravity of the aromatic ring delineated by the C5/C6/C1−C3/N4 atoms. Symmetry code: (ii) *x* − 1/2, *y*, −*z*
